# Synthesis of Biobased Phloretin Analogues: An Access to Antioxidant and Anti-Tyrosinase Compounds for Cosmetic Applications

**DOI:** 10.3390/antiox10040512

**Published:** 2021-03-25

**Authors:** Laurène Minsat, Cédric Peyrot, Fanny Brunissen, Jean-Hugues Renault, Florent Allais

**Affiliations:** 1 URD Agro-Biotechnologies Industrielles (ABI), CEBB (Centre Européen de Biotechnologie et de Bioéconomie), AgroParisTech, 51110 Pomacle, France; laurene.minsat@agroparistech.fr (L.M.); fanny.brunissen@agroparistech.fr (F.B.); 2 Université de Reims Champagne-Ardenne, CNRS, ICMR 7312, 51097 Reims, France; Jean-hugues.renault@univ-reims.fr

**Keywords:** phloretin analogues, chalcones, dihydrochalcones, Claisen–Schmidt condensation, DPPH test, anti-tyrosinase, antioxidant

## Abstract

The current cosmetic and nutraceutical markets are characterized by a strong consumer demand for a return to natural products that are less harmful to both the consumers and the environment than current petrosourced products. Phloretin, a natural dihydrochalcone (DHC) found in apple, has been widely studied for many years and identified as a strong antioxidant and anti-tyrosinase ingredient for cosmetic formulations. Its low concentration in apples does not allow it to be obtained by direct extraction from biomass in large quantities to meet market volumes and prices. Moreover, its remarkable structure prevents its synthesis through a green process. To overcome these issues, the synthesis of phloretin analogs appears as an alternative to access valuable compounds that are potentially more active than phloretin itself. Under such considerations, 12 chalcones (CHs) and 12 dihydrochalcones (DHCs) were synthesized through a green Claisen–Schmidt condensation using bio-based reagents. In order to evaluate the potential of these molecules, radical scavenging DPPH and anti-tyrosinase tests have been conducted. Moreover, the UV filtering properties and the stability of these analogs towards UV-radiations have been evaluated. Some molecules showed competitive antioxidant and anti-tyrosinase activities regarding phloretin. Two compounds in particular showed EC_50_ lower than phloretin, one chalcone and one dihydrochalcone.

## 1. Introduction

**Figure 1:**
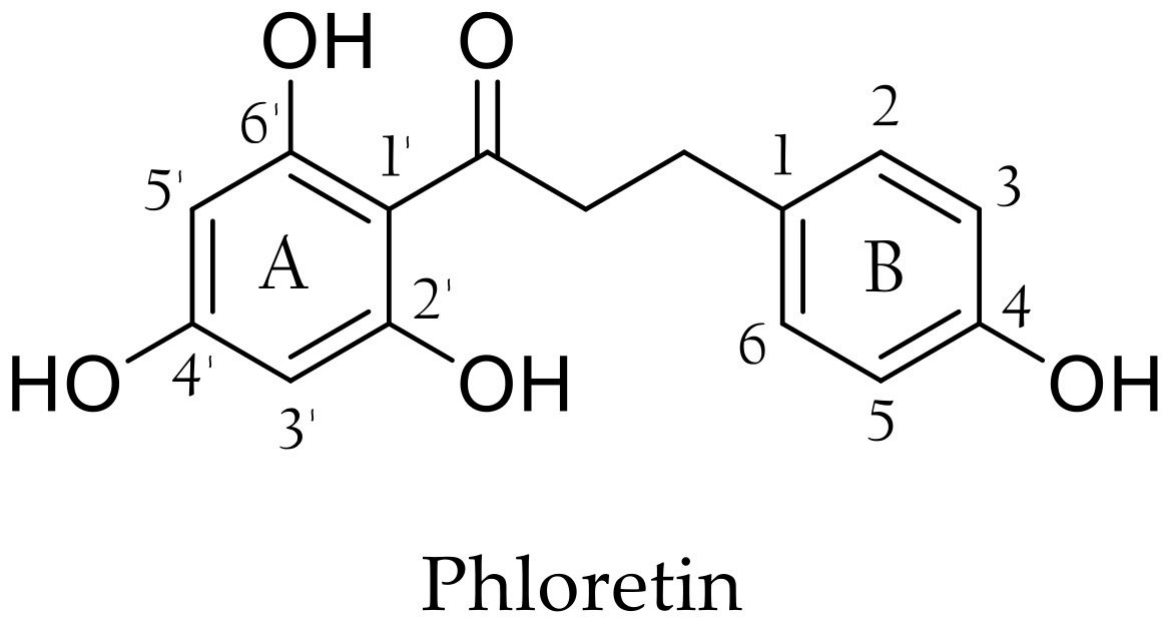
Structure of phloretin.

In the past ten years, special attention has been paid to phloretin, a naturally occurring flavonoid found in apple especially in peels [1] but more consistently in leaves [2], branches, barks and roots [3]. It can be recovered from processed apple products and by-products, such as apple pomace resulting from cider production [4]. Phloretin is a dihydrochalcone (DHC) composed of two aromatic rings, commonly named A and B, bearing hydroxy groups in positions 2′, 4, 4′ and 6′ connected by a three-carbon α,β-unsaturated carbonyl system (Figure 1). This remarkable structure provides phloretin with many interesting biological activities that have been widely studied [5,6,7,8,9,10,11]. For instance, Barreca et al. demonstrated strong antimicrobial activities for phloretin in particular against Gram-positive bacteria such as *Staphylococcus aureus* and *Listeria monocytogenes.* They also highlighted the importance of the free hydroxy moiety in position 2′ and the absence of glycosyl moiety (compared to phlorizin, the best-known phloretin glycoside) [12]. Antimicrobial properties are widely sought in view of the many criticisms against the main preservatives used in cosmetics, such as parabens and phenoxyethanol [13]. In addition, phloretin inhibits the formation of some bacterial biofilms, which is a serious human health issue due to these bacteria’s increasing resistance to antimicrobial treatments and their ubiquity in medical and engineering environments [14,15].

Several studies also reported strong antioxidant activities, such as that of Rezk et al., who linked it to the presence of the three hydroxyls groups on the A-ring and the carbonyl group [16]. In fact, phloretin is able to (1) scavenge hydroxyl radicals, stable free radicals such as 2,2-diphenyl-1-picrylhydrazyl (DPPH), peroxynitrite, and (2) prevent lipid peroxidation [16,17].

Additionally, as skin cancers are increasing considerably, linked to overexposure to the sun [18,19], phloretin acts also as a potential inhibitor of tyrosinase, the key enzyme in skin pigment disorders. Indeed, phloretin showed a significant inhibition of the activity of mushroom tyrosinase in vitro and tyrosinase from human melanocytes [20,21]. Therefore, it can be used as a topical lightening agent in cosmetics to treat skin pigmentary disorder. Recently, a study led by L’Oreal USA^®^ showed the UV protection for human skin provided by phloretin formulated with vitamin C and ferulic acid. Furthermore, it stabilizes the formulation and increases the skin availability of the other components [22]. Indeed, phloretin can be used as a skin penetration enhancer in formulations thanks to its ability to interact with bilayer membranes, increasing their fluidity [11,23].

**Scheme 1:**
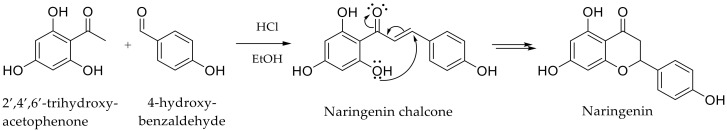
Intramolecular cyclization occurring during phloretin synthesis through a Claisen-Schmidt condensation catalyzed by HCl.

In this context, it is not surprising to find phloretin as an active ingredient in cosmetic formulations. However, the small amount of this compound present in the biomass pushes us to design chemical synthestic pathways, allowing us to meet the potential increase in demand. Therefore, it appears interesting to explore its chemical synthesis or hemi-synthesis through a green process to fulfill the current requirements of targeted markets. Nevertheless, only a few studies have reported such synthetic approaches. Siddaiah et al. proposed Friedel–Crafts acylation pathway between phenols and dihydrocinnamic acids using borontrifluoride etherate (BF_3_.Et_2_O), but phloretin was isolated in poor yields (30%) [24]. Another pathway was proposed, requiring protection steps for the phloretin derivatives synthesis. It used benzyl groups to prevent the intramolecular cyclization due to the hydroxyl group in the *ortho* position of phloretin precursor, the naringenin chalcone [25]. Indeed, this hydroxyl constitutes a real limitation as it readily leads to a Michael addition to form naringenin (Scheme 1).

To overcome these issues, the synthesis of phloretin analogs appears as an alternative to access valuable compounds potentially more active than phloretin itself. The easiest pathway to access DHCs consists of (1) synthesizing the corresponding chalcone, and (2) reducing it in presence of hydrogen with palladium on carbon [26]. Chalcones (CHs) are α,β-unsaturated analogs of DHCs, naturally occurring in various plants and widely studied for many years for their biological properties [27,28]. While the reduction step (2) is rather classic, there are many ways to synthesize CHs (1). Claisen–Schmidt condensation between substituted or unsubstituted acetophenone and benzaldehyde is the most frequently used method [27,29,30]. This reaction can be performed in acidic or basic conditions in polar solvents. In general, basic reagents are preferred, mainly NaOH [31], KOH [26], NaH [32], as they often lead to high yields (40–80%) while acidic reagents, such as HCl or boric acid, lead to a wider range of yields from 10% to 80% [28,33,34]. However, the use of an acid catalyst allows side reactions to be prevented, such as the Cannizzaro reaction, which occurs in strongly alkaline conditions [35]. Recently, greener conditions were developed using reusable catalysts such as Amberlyst-15 and Amberlite-200C, which are commercial acid-resins, or even solid base catalyst (e.g., poly(*N*-vinylimidazole)) coupled with ultrasonic irradiation under solvent-free conditions [36,37]. Although Claisen–Schmidt condensation is the most widely used method due to its simple procedure, other approaches have been described. Different coupling reactions, for instance, Sonogashira [38], Heck [39] or Suzuki couplings [40], were employed as novel strategies for molecule synthesis including chalcones. Xu et al. described an innovative microwave-assisted Wittig reaction leading to excellent yields, but only in small quantities and not quite sustainable for the industry [41].

The purpose of this work is to first synthesize a library of 24 phloretin analogs—including seven new compounds—with higher potential than phloretin itself by modulating the substituents on A and B rings to tune the properties for a potential valorization in the cosmetic or nutraceutical sector. In order to limit the use of petrosourced precursors, metal catalysts and multi-step synthesis, and to respect the green chemistry principles, we aimed at synthetizing phloretin analogs through a simple two-step process: an HCl-catalyzed Claisen–Schmidt condensation using bio-based reagents, followed by a catalytic palladium-catalyzed hydrogenation. This method not only offers various advantages such as simple and quick procedures and high reaction yields, but also avoids side reactions or protection steps. Moreover, the use of HCl in catalytic amount significantly limits the environmental impact of this approach. The antioxidant properties, the anti-tyrosinase activity and the anti-UV activity of the 24 analogs were then assessed to establish Structure–Activity Relationships. To the best of our knowledge, this is the first time that the anti-tyrosinase and anti-UV properties of phloretin analogs have been determined and discussed.

## 2. Materials and Methods

### 2.1. Materials

All reagents and solvents were purchased from Sigma Aldrich (St. Louis, MO, USA), TCI (Tokyo, Japan) or Fisher Scientific (Hampton, NH, USA). All chemicals were used directly without purification.

### 2.2. Purification and Characterization

The CH and DHC purifications were performed by flash chromatography on a flash-prep PuriFlash^®^ 4100 LC system from Interchim (Montluçon, France) with prepacked silica columns (30 µm, Interchim PF-Si30-HP) and dual-wavelength collection (λ = 254 and 280 nm). Mixtures of cyclohexane and ethyl acetate were used as eluant.

The CHs and DHCs characterizations were realized by NMR and mass spectrometry. ^1^H-NMR spectra were recorded on a Brucker Fourier 300 (300 MHz) (Billerica, MA, USA). Residual Acetone-*d*_6_ and DMSO-*d*_6_ protons signals at δ 2.05 and 2.5 ppm, respectively, were used for the calibration. Data are reported as follows: chemical shift (δ ppm), multiplicity (s = singlet, d = doublet, t = triplet, dd = doublet of doublets, and m = multiplet), coupling constant (Hz), integration and assignment. ^13^C-NMR spectra were recorded on a Bruker Fourier 300 (75 MHz) (Billerica, MA, USA). Residual Acetone-*d*_6_ and DMSO-*d*_6_ protons signals at δ 29.84 ppm and 39.52 ppm, respectively, were used for the calibration. Data are reported as follows: chemical shift (δ ppm), assignment. All NMR assignments were made using COSY, HMBC and HSQC spectra. 1D and 2D NMR spectra (^1^H, ^13^C, HSQC, HMBC, and COSY) were recorded using standard Bruker pulse programs.

High-resolution mass spectrometry was performed on an Agilent, 1290 system, equipped with a PDA UV detector and a 6545 Q-TOF mass spectrometer (Wilmington, DE, USA). The source was equipped with a JetStream ESI probe operating at atmospheric pressure. The electrospray interface operated with the following parameters: scanning range of 50–1000, nebulizer 35 psi, gas temperature 325 °C, gas flow 8 L/min, sheath da temperature 350 °C, sheath gas flow 11 L/min. Elution was performed using a Zorbax Eclipse plus C18 (1.8 μm, 50 × 2.1 mm^2^; Agilent), with the following mobile phases: 0.1% formic acid in water (solvent A) and acetonitrile (solvent B). The flow rate was set at 0.4 mL/min and according to the gradient 0−3 min at 5% of B, 3−4 min from 5% to 10% B, 4−13 min from 10% to 99% B, 13−16 min at 99% B, 16−18 min from 99% to 5% B. The column was heated at 40 °C and the sample injection volume was 1 μL.

FTIR spectra were recorded on a Cary 630 FTIR Spectrometer by Agilent (Wilmington, DE, USA).

Melting points were recorded on a Mettler Toledo MP50 Melting Point System (Greifensee, Switzerland) (heating at 3 °C/min).

### 2.3. General Procedure for Chalcones Synthesis

All CHs were synthesized by a Claisen–Schmidt condensation using EtOH as solvent and HCl as catalyst. The corresponding substituted acetophenone (1.0 equiv) and benzaldehyde (1.2 equiv) were dissolved in ethanol in a round-bottom flask topped by an air cooling column. A few drops of HCl (37%) were added, and the reaction mixture was refluxed overnight with continued stirring. The solvent was evaporated under reduced pressure, and the product was dry loaded onto Celite^®^ to be purified through flash chromatography on silica gel.

For each compound, the numbers for the NMR assignment are related to the labeling in the NMR spectra in the Supplementary Materials.

**4,4′-dihydroxy-chalcone (a1)**: purification (cyclohexane/ethyl acetate: 50/50) led to a yellow powder (76%). m.p. 202–203 °C, UV: λ_max_ (EtOH, nm) 350, ε (L·mol^−1^·cm^−1^) 87,739. FTIR (pure) ν (cm^−1^): 3465, 3428, 1639, 1586. ^1^H-NMR (300 MHz, Acetone-*d*_6_) δ: 8.07 (d, *J* = 8.8 Hz, 2H, H9-9′), 7.70 (d, *J* = 8.6 Hz, 2H, H5-5′), 7.69 (s, 2H, H2 and H3), 6.96 (d, *J* = 8.6 Hz, 2H, H10-10′), 6.91 (d, *J* = 8.7 Hz, 2H, H6-6′). ^13^C-NMR (75 MHz, Acetone-*d*_6_) δ: 188.0 (C1), 162.5 (C11), 160.6 (C7), 144.0 (C3), 131.7 (C9-9′), 131.4 (C8), 131.3 (C5-5′), 127.9 (C4), 119.7 (C2), 116.7 (C6-6′), 116.1 (C10-10′). HRMS: *m*/*z* [M+H]^+^ calculated for C_15_H_13_O_3_: 241.0864 found: 241.0864.

**3,4,4′-trihydroxy-chalcone (a2)**: purification (cyclohexane/ethyl acetate: 50/50) led to a yellow powder (80%). m.p. 189–190 °C, UV: λ_max_ (EtOH, nm) 365, ε (L·mol^−1^·cm^−1^) 29,139. FTIR (pure) ν (cm^−1^): 3476–3191, 1644, 1589. ^1^H-NMR (300 MHz, DMSO-*d*_6_) δ: 8.01 (d, *J* = 8.7 Hz, 2H, H11-11′), 7.59 (d, *J* = 15.5 Hz, 1H, H3), 7.52 (d, *J* = 15.4 Hz, 1H, H2), 7.22 (d, *J* = 1.6 Hz, 1H, H5), 7.15 (dd, *J* = 1.7 Hz, 8.2 Hz, 1H, H9), 6.87 (d, *J* = 8.6 Hz, 2H, H12-12′), 6.79 (d, *J* = 8.2 Hz, 1H, H8). ^13^C-NMR (75 MHz, DMSO-*d*_6_) δ: 187.1 (C1), 161.9 (C13), 148.4 (C7), 145.6 (C6), 143.7 (C3), 130.9 (C11-11′), 129.5 (C10), 126.5 (C4), 121.9 (C9), 118.4 (C2), 115.8 (C8), 115.4 (C5), 115.3 (C12-12′). HRMS: *m*/*z* [M+H]^+^ calculated for C_15_H_13_O_4_: 257.0814 found: 257.0815.

**3′,4,4′-trihydroxy-chalcone (a3)**: purification (cyclohexane/ethyl acetate: 50/50) led to a yellow powder (68%). m.p. 176–177 °C, UV: λ_max_ (EtOH, nm) 358, ε (L·mol^−1^·cm^−1^) 30,628. FTIR (pure) ν (cm^−1^): 3425–3198, 1631, 1597. ^1^H-NMR (300 MHz, DMSO-*d*_6_) δ: 7.68 (d, *J* = 8.6 Hz, 2H, H5-5′), 7.58 (m, 3H, H2, H3 and H9), 7.49 (d, *J* = 1.8 Hz, 1H, H13), 6.85 (d, *J* = 7.9 Hz, 1H, H10), 6.82 (d, *J* = 8.0 Hz, 2H, H6-6′). ^13^C-NMR (75 MHz, DMSO-*d*_6_) δ: 187.1 (C1), 159.8 (C7), 150.6 (C11), 145.4 (C12), 142.9 (C3), 130.7 (C5-5′), 129.9 (C8), 126.0 (C4), 121.8 (C9), 118.6 (C2), 115.8 (C6-6′), 115.3 (C10 or C13), 115.1 (C10 or C13). HRMS: *m*/*z* [M+H]^+^ calculated for C_15_H_13_O_4_: 257.0814 found: 257.0815.

**3,3′,4,4′-tetrahydroxy-chalcone (a4)**: purification (cyclohexane/ethyl acetate: 45/55) led to a yellow powder (62%). m.p. 209–210 °C, UV: λ_max_ (EtOH, nm) 371, ε (L·mol^−1^·cm^−1^) 47,295. FTIR (pure) ν (cm^−1^): 3472, 3267, 1635, 1592. ^1^H-NMR (300 MHz, DMSO-*d*_6_) δ: 7.56 (dd, *J* = 1.7 Hz, 8.25 Hz, 1H, H11), 7.50 (s, 2H, H2 and H3), 7.47 (d, *J* = 1.7 Hz, 1H, H15), 7.20 (s, 1H, H5), 7.13 (dd, *J* = 1.4 Hz, 8.2 Hz, 1H, H9), 6.84 (d, *J* = 8.2 Hz, 1H, H12), 6.79 (d, *J* = 8.1 Hz, 1H, H8). ^13^C-NMR (75 MHz, DMSO-*d*_6_) δ: 187.1 (C1), 150.6 (C13), 148.4 (C7), 145.6 (C6 or C14), 145.5 (C6 or C14), 143.4 (C3), 130.0 (C10), 126.5 (C4), 121.8 (C9 and C11), 118.5 (C2), 115.8 (C8), 115.3 (C5 and C15), 115.1 (C12). HRMS: *m*/*z* [M+H]^+^ calculated for C_15_H_13_O_5_: 273.0763 found: 273.0762.

**4,4′-dihydroxy-3-methoxy-chalcone (a5)**: purification (cyclohexane/ethyl acetate: 50/50) led to a yellow powder (80%). m.p. 224–227 °C, UV: λ_max_ (EtOH, nm) 362, ε (L·mol^−1^·cm^−1^) 33,032. FTIR (pure) ν (cm^−1^): 3395, 1638, 1587. ^1^H-NMR (300 MHz, DMSO-*d*_6_) δ: 8.06 (d, *J* = 8.7 Hz, 2H, H11-11′), 7.73 (d, *J* = 15.5 Hz, 1H, H2), 7.60 (d, *J* = 15.5 Hz, 1H, H3), 7.49 (d, *J* = 1.6 Hz, 1H, H5), 7.24 (dd, *J* = 1.7 Hz, 8.2 Hz, 1H, H9), 6.89 (d, *J* = 8.7 Hz, 2H, H12-12′), 6.82 (d, *J* = 8.1 Hz, 1H, H8), 3.87 (s, 3H, H14). ^13^C-NMR (75 MHz, DMSO-*d*_6_) δ: 187.0 (C1), 162.0 (C13), 149.4 (C7), 148.0 (C6), 143.7 (C3), 131.0 (C11-11′), 129.4 (C10), 126.5 (C4), 123.9 (C9), 118.7 (C2), 115.6 (C8), 115.3 (C12-12′), 111.5 (C5), 55.8 (C14). HRMS: *m*/*z* [M+H]^+^ calculated for C_16_H_15_O_4_: 271.0970 found: 271.0973.

**4,4′-dihydroxy-3′-methoxy-chalcone (a6)**: purification (cyclohexane/ethyl acetate: 45/55) led to a yellow powder (78%). m.p. 187–188 °C, UV: λ_max_ (EtOH, nm) 360, ε (L·mol^−1^·cm^−1^) 29,982. FTIR (pure) ν (cm^−1^): 3383, 3211, 1645, 1587. ^1^H-NMR (300 MHz, Acetone-*d*_6_) δ: 7.78 (dd, *J* = 2.0 Hz, 8.3 Hz, 1H, H9), 7.69 (m, 5H, H2, H3, H5-5′ and H13), 6.95 (d, *J* = 8.3 Hz, 1H, H10), 6.91 (d, *J* = 8.7 Hz, 2H, H6-6′) 3.94 (s, 3H, H14). ^13^C-NMR (75 MHz, Acetone-*d*_6_) δ: 187.9 (C1), 160.6 (C7), 152.1 (C11), 148.5 (C12), 143.9 (C3), 131.8 (C8), 131.3 (C5-5′), 127.9 (C4), 124.1 (C9), 119.6 (C2), 116.7 (C6-6′), 115.4 (C10), 112.0 (C13), 56.3 (C14). HRMS: *m*/*z* [M+H]^+^ calculated for C_16_H_15_O_4_: 271.0970 found: 271.0972.

**3,4,4′-trihydroxy-3-methoxy-chalcone (a7)**: purification (cyclohexane/ethyl acetate: 45/55) led to a yellow powder (72%). m.p. 172–173 °C, UV: λ_max_ (EtOH, nm) 372, ε (L·mol^−1^·cm^−1^) 37,725. FTIR (pure) ν (cm^−1^): 3450, 3125, 1640, 1588. ^1^H-NMR (300 MHz, Acetone-*d*_6_) δ: 7.77 (dd, *J* = 2.0 Hz, 8.28 Hz, 1H, H11), 7.69 (d, *J* = 1.9 Hz, 1H, H15), 7.64 (s, 2H, H2 and H3), 7.31 (d, *J* = 2.1 Hz, 1H, H5), 7.18 (dd, *J* = 2.1 Hz, 8.19 Hz, 1H, H9), 6.95 (d, *J* = 8.3 Hz, 1H, H12), 6.89 (d, *J* = 8.2 Hz, 1H, H8), 3.94 (s, 3H, H16). ^13^C-NMR (75 MHz, Acetone-*d*_6_) δ: 187.86 (C1), 152.11 (C13), 148.71 (C6), 148.48 (C14), 146.32 (C7), 144.32 (C3), 131.82 (C10), 128.55 (C4), 124.09 (C11), 122.97 (C9), 119.71 (C2), 116.36 (C8), 115.66 (C5), 115.42 (C12), 112.02 (C15), 56.31 (C16). HRMS: *m*/*z* [M+H]^+^ calculated for C_16_H_15_O_5_: 287.0919 found: 287.0917.

**3′,4,4′-trihydroxy-3-methoxy-chalcone (a8)**: purification (cyclohexane/ethyl acetate: 45/55) led to a yellow powder (78%). Degradation temperature 186–187 °C, UV: λ_max_ (EtOH, nm) 365, ε (L·mol^−1^·cm^−1^) 31,067. FTIR (pure) ν (cm^−1^): 3517–3234, 1647, 1591. ^1^H-NMR (300 MHz, DMSO-*d*_6_) δ: 7.68 (d, *J* = 15.5 Hz, 1H, H2), 7.63 (dd, *J* = 2.1 Hz, 8.4 Hz, 1H, H11), 7.57 (d, *J* = 15.4 Hz, 1H, H3), 7.5 (d, *J* = 2.1 Hz, 1H, H15), 7.47 (d, *J* = 1.8 Hz, 1H, H5), 7.22 (dd, *J* = 1.8 Hz, 8.3 Hz, 1H, H9), 6.85 (d, *J* = 8.3 Hz, 1H, H12), 6.81 (d, *J* = 8.1 Hz, 1H, H8), 3.86 (s, 3H, H16). ^13^C-NMR (75 MHz, DMSO-*d*_6_) δ: 187.1 (C1), 150.6 (C13), 149.3 (C7), 148.0 (C6), 145.4 (C14), 143.3 (C3), 130.0 (C10), 126.5 (C4), 123.8 (C9), 121.9 (C11), 118.8 (C2), 115.6 (C8), 115.4 (C15), 115.0 (C12), 111.4 (C5), 55.8 (C16). HRMS: *m*/*z* [M+H]^+^ calculated for C_16_H_15_O_5_: 287.0919 found: 287.0922.

**4,4′-dihydroxy-3′,5′-dimethoxy-chalcone (a9)**: purification (cyclohexane/ethyl acetate: 50/50) led to a yellow powder (65%). m.p. 170–171 °C, UV: λ_max_ (EtOH, nm) 363, ε (L·mol^−1^·cm^−1^) 27,390. FTIR (pure) ν (cm^−1^): 3508, 1637, 1591. ^1^H-NMR (300 MHz, Acetone-*d*_6_) δ: 7.73 (s, 2H, H2 and H3), 7.69 (d, *J* = 8.6 Hz, 2H, H5-5′), 7.49 (s, 2H, H9-9′), 6.91 (d, *J* = 8.6 Hz, 2H, H6-6′), 3.93 (s, 6H, H12-12′). ^13^C-NMR (75 MHz, Acetone-*d*_6_) δ: 187.9 (C1), 160.6 (C7), 148.6 (C10-10′), 144.1 (C3), 141.8 (C11), 131.4 (C5-5′), 130.3 (C8), 127.9 (C4), 119.6 (C2), 116.7 (C6-6′), 107.3 (C9-9′), 56.8 (C12-12′). HRMS: *m*/*z* [M+H]^+^ calculated for C_17_H_17_O_5_: 301.1076 found: 301.1078.

**4,4′-dihydroxy-3,3′-dimethoxy-chalcone (a10)**: purification (cyclohexane/ethyl acetate: 50/50) led to a yellow powder (69%). m.p. 126–127 °C, UV: λ_max_ (EtOH, nm) 369, ε (L·mol^−1^·cm^−1^) 29,411. FTIR (pure) ν (cm^−1^): 3368, 1641, 1589. ^1^H-NMR (300 MHz, Acetone-*d*_6_) δ: 7.70 (dd, *J* = 2.0 Hz, 8.3 Hz, 1H, H11), 7.72 (d, *J* = 3.5 Hz, 2H, H2 and H3), 7.68 (d, *J* = 2.0 Hz, 1H, H15), 7.48 (d, *J* = 2.0 Hz, 1H, H5), 7.30 (dd, *J* = 2.0 Hz, 8.2 Hz, 1H, H9), 6.94 (d, *J* = 8.3 Hz, 1H, H12), 6.89 (d, *J* = 8.2 Hz, 1H, H8), 3.94 (s, 3H, H16 or H17), 3.93 (s, 3H, H16 or H17). ^13^C-NMR (75 MHz, Acetone-*d*_6_) δ: 187.8 (C1), 152.1 (C13), 150.1 (C7), 148.7 (C6), 148.5 (C14), 144.4 (C3), 131.8 (C10), 128.3 (C4), 124.2 (C9), 124.1 (C11), 119.8 (C2), 116.1 (C8), 115.3 (C12), 112 (C15), 111.9 (C5), 56.4 (C16 or C17), 56.3 (C16 or C17). HRMS: *m*/*z* [M+H]^+^ calculated for C_17_H_17_O_5_: 301.1076 found: 301.1077.

**4,4′-dihydroxy-3,3′,5-trimethoxy-chalcone (a11)**: purification (cyclohexane/ethyl acetate: 50/50) led to a yellow powder (47%). m.p. 159–160 °C, UV: λ_max_ (EtOH, nm) 375, ε (L·mol^−1^·cm^−1^) 27,889. FTIR (pure) ν (cm^−1^): 3514, 3366, 1643, 1590. ^1^H-NMR (300 MHz, DMSO-*d*_6_) δ: 7.82 (dd, *J* = 2.0 Hz, 8.4 Hz, 1H, H9), 7.77 (d, *J* = 15.5 Hz, 1H, H2), 7.62 (d, *J* = 15.3 Hz, 1H, H3),7.60 (d, *J* = 1.9 Hz, 1H, H13), 7.18 (s, 2H, H5-5′), 6.91 (d, *J* = 8.3 Hz, 1H, H10), 3.86 (s, 3H, H15), 3.84 (s, 6H, H14-14′). ^13^C-NMR (75 MHz, DMSO-*d*_6_) δ: 186.95 (C1), 151.81 (C11), 148.09 (C6-6′), 147.85 (C12), 143.97 (C3), 138.44 (C7), 129.77 (C8), 125.29 (C4), 123.70 (C9), 119.02 (C2), 114.87 (C10), 111.53 (C13), 106.78 (C5-5′), 56.22 (C14-14′), 55.71 (C15). HRMS: *m*/*z* [M+H]^+^ calculated for C_18_H_19_O_6_: 331.1181 found: 331.1181.

**4,4′-dihydroxy-3,3′,5,5′-tetramethoxy-chalcone (a12)**: purification (cyclohexane/ethyl acetate: 50/50) led to a yellow powder (52%). m.p. 187–188 °C, UV: λ_max_ (EtOH, nm) 377, ε (L·mol^−1^·cm^−1^) 30,047. FTIR (pure) ν (cm^−1^): 3304, 1749, 1633, 1582. ^1^H-NMR (300 MHz, DMSO-*d*_6_) δ: 7.77 (d, *J* = 15.5 Hz, 1H, H2), 7.65 (d, *J*=15.3 Hz, 1H, H3), 7.44 (s, 2H, H9-9′), 7.20 (s, 2H, H5-5′), 3.88 (s, 6H, H13-13′), 3.85 (s, 6H, H12-12′). ^13^C-NMR (75 MHz, DMSO-*d*_6_) δ: 187.1 (C1), 148.09 (C6-6′), 147.34 (C10-10′), 144.26 (C3), 141.07 (C11), 138.57 (C7), 128.44 (C8), 125.27 (C4), 119.12 (C2), 107.05 (C5-5′), 106.83 (C9-9′), 56.39 (C13-13′), 56.28 (C12-12′). HRMS: *m*/*z* [M+H]^+^ calculated for C_19_H_21_O_7_: 361.1287 found: 361.1290.

### 2.4. General Procedure for Dihydrochalcones Syntheses

All DHCs were obtained by catalytic hydrogenation of the corresponding CH. The CH (400 mg) was solubilized in EtOH (100 mL), 10 wt.% of palladium on carbon (Pd/C), was added and the mixture was stirred for a few minutes under nitrogen to remove air before being placed under a continuous flow of H_2_ with vigorous stirring (to form a vortex) for 45 min. Then, the reaction was replaced under nitrogen to remove residual H_2_ for a few minutes. The reaction mixture was filtered on a sinter filter with Celite^®^ to remove the Pd/C. The solvent was evaporated under reduced pressure and the product was dry-loaded onto Celite^®^ to be purified through flash chromatography on silica gel.

For each compound, the numbers for the NMR assignment are related to the labeling in the NMR spectra in the Supplementary Materials.

**4,4′-dihydroxy-dihydrochalcone (b1)**: purification led to a white powder (61%). m.p. 161–163 °C, UV: λ_max_ (EtOH, nm) 279, ε (L·mol^−1^·cm^−1^) 22,800. FTIR (pure) ν (cm^−1^): 3417, 3121, 1652. ^1^H-NMR (300 MHz, Acetone-*d*_6_) δ: 7.91 (d, *J* = 8.8 Hz, 2H, H9-9′), 7.10 (d, *J* = 8.4 Hz, 2H, H5-5′), 6.91 (d, *J* = 8.8 Hz, 2H, H10-10′), 6.74 (d, *J* = 8.4 Hz, 2H, H6-6′), 3.20 (t, *J* = 7.6 Hz, 2H, H2), 2.89 (t, *J* = 7.5 Hz, 2H, H3). ^13^C-NMR (75 MHz, Acetone-*d*_6_) δ: 197.8 (C1), 162.6 (C11), 156.4 (C7), 133.3 (C4), 131.3 (C9-9′), 130.3 (C8), 130.2 (C5-5′), 115.99 (C6-6′), 159.96 (C10-10′), 40.8 (C2), 30.2 (C3). HRMS: *m*/*z* [M+H]^+^ calculated for C_15_H_15_O_3_: 243.1021 found: 243.1024.

**3,4,4′-trihydroxy-dihydrochalcone (b2)**: purification (cyclohexane/ethyl acetate: 55/45) led to a brownish solid (69%). m.p. 135–136 °C, UV: λ_max_ (EtOH, nm) 281, ε (L·mol^−1^·cm^−1^) 21,157. FTIR (pure) ν (cm^−1^): 3350, 1639. ^1^H-NMR (300 MHz, Acetone-*d*_6_) δ: 7.92 (d, *J* = 8.7 Hz, 2H, H11-11′), 6.91 (d, *J =* 8.7 Hz, 2H, H12-12′), 6.76 (d, *J* = 1.7 Hz, 1H, H5), 6.72 (d, *J* = 8.0 Hz, 1H, H8), 6.60 (dd, *J* = 1.6 Hz, 8.0 Hz, 1H, H9), 3.18 (t, *J* = 7.6 Hz, 2H, H2), 2.84 (t, *J* = 7.6 Hz, 2H, H3). ^13^C-NMR (75 MHz, Acetone-*d*_6_) δ: 197.8 (C1), 162.5 (C13), 145.8 (C6), 144.0 (C7), 134.3 (C4), 131.3 (C11-11′), 130.3 (C10), 120.4 (C9), 116.4 (C5), 116.0 (C12-12′ and C8), 40.7 (C2), 30.4 (C3). HRMS: *m*/*z* [M+H]^+^ calculated for C_15_H_15_O_4_: 259.0970 found: 259.0965.

**3′,4,4′-trihydroxy-dihydrochalcone (b3)**: purification (cyclohexane/ethyl acetate: 65/35) led to a white powder (58%). m.p. 159–160 °C, UV: λ_max_ (EtOH, nm) 278, ε (L·mol^−1^·cm^−1^) 15,921. FTIR (pure) ν (cm^−1^): 3233, 3133, 1650. ^1^H-NMR (300 MHz, Acetone-*d*_6_) δ: 7.47 (m, 2H, H13 and H9), 7.10 (d, *J* = 8.4 Hz, 2H, H5-5), 6.89 (d, *J* = 8.1 Hz, 1H, H10), 6.74 (d, *J* = 8.4 Hz, 2H, H6-6′), 3.17 (t, *J* = 7.6 Hz, 2H, H2), 2.88 (t, *J* = 7.6 Hz, 2H, H3). ^13^C-NMR (75 MHz, Acetone-*d*_6_) δ: 197.9 (C1), 156.4 (C7), 150.9 (C11), 145.8 (C12), 133.3 (C4), 130.8 (C8), 130.2 (C5-5′), 122.5 (C9), 116.0 (C6-6′), 115.6 (C10 and C13), 40.7 (C2), 30.3 (C3). HRMS: *m*/*z* [M+H]^+^ calculated for C_15_H_15_O_4_: 259.0970 found: 259.0972.

**3,3′,4,4′-tetrahydroxy-dihydrochalcone (b4)**: purification (cyclohexane/ethyl acetate: 60/40) led to a white powder (69%). m.p. 186–188 °C, UV: λ_max_ (EtOH, nm) 280, ε (L·mol^−1^·cm^−1^) 15,752. FTIR (pure) ν (cm^−1^): 3422, 3280, 3220, 1638. ^1^H-NMR (300 MHz, Acetone-*d*_6_) δ: 7.47 (m, 2H, H15 and H11), 6.89 (d, *J* = 8.1 Hz, 1H, H12), 6.75 (d, *J* = 1.7 Hz, 1H, H5), 6.72 (d, *J* = 8.0 Hz, 1H, H8), 6.59 (dd, *J* = 1.7 Hz, 8.0 Hz, 1H, H9), 3.15 (t, *J* = 7.6 Hz, 2H, H2), 2.83 (t, *J* = 7.6 Hz, 2H, H3). ^13^C-NMR (75 MHz, Acetone-*d*_6_) δ: 197.9 (C1), 150.9 (C13), 145.8 (C14), 145.7 (C16), 144.0 (C7), 134.3 (C4), 130.8 (C10), 122.5 (C11), 120.4 (C9), 116.4 (C5), 116.0 (C8), 115.64 (C12 or C15), 115.62 (C12 or C15), 40.7 (C2), 30.5 (C3). HRMS: *m*/*z* [M+H]^+^ calculated for C_15_H_15_O_5_: 275.0919 found: 275.0918.

**4,4′-dihydroxy-3-methoxy-dihydrochalcone (b5)**: purification (cyclohexane/ethyl acetate: 55/45) led to a white powder (64%). m.p. 142–143 °C, UV: λ_max_ (EtOH, nm) 280, ε (L·mol^−1^·cm^−1^) 17,997. FTIR (pure) ν (cm^−1^): 3317, 1656. ^1^H-NMR (300 MHz, Acetone-*d*_6_) δ: 7.79 (d, *J* = 8.7 Hz, 2H, H11-11′), 6.77 (m, 3H, H5 and H12-12′), 6.59 (m, 2H; H8 and H9), 3.69 (s, 3H, H14), 3.10 (t, *J* = 7.7 Hz, 2H, H2), 2.77 (t, *J* = 7.6 Hz, 2H, H3). ^13^C-NMR (75 MHz, Acetone-*d*_6_) δ: 197.9 (C1), 162.6 (C13), 148.2 (C6), 145.6 (C7), 133.9 (C4), 131.3 (C11-11′), 130.3 (C10), 121.6 (C9), 116.0 (C12-12′), 115.6 (C8), 122.9 (C5), 56.2 (C14), 40.8 (C2), 30.7 (C3). HRMS: *m*/*z* [M+H]^+^ calculated for C_16_H_17_O_4_: 273.1127 found: 273.1126.

**4,4′-dihydroxy-3′-methoxy-dihydrochalcone (b6)**: purification (cyclohexane/ethyl acetate: 70/30) led to a white powder (56%). m.p. 150–151 °C, UV: λ_max_ (EtOH, nm) 277, ε (L·mol^−1^·cm^−1^) 14,793. FTIR (pure) ν (cm^−1^): 3320, 1658. ^1^H-NMR (300 MHz, Acetone-*d*_6_) δ: 7.60 (dd, *J* = 1.9 Hz, 8.3 Hz, 1H, H9), 7.55 (d, *J* = 1.7 Hz, 1H, H13), 7.11 (d, *J* = 8.4 Hz, 2H, H5-5′), 6.90 (d, *J* = 8.2 Hz, 1H, H10), 6.74 (d, *J* = 8.5 Hz, 2H, H6-6′), 3.90 (s, 3H, H14), 3.22 (t, *J* = 7.6 Hz, 2H, H2), 2.90 (t, *J* = 7.6 Hz, 2H, H3). ^13^C-NMR (75 MHz, Acetone-*d*_6_) δ: 197.8 (C1), 156.4 (C7), 152.2 (C11), 148.3 (C12), 133.3 (C4), 130.5 (C8), 130.2 (C5-5′), 123.8 (C9), 116.0 (C6-6′), 115.4 (C10), 111.5 (C13), 56.3 (C14), 40.7 (C2), 30.3 (C3). HRMS: *m*/*z* [M+H]^+^ calculated for C_16_H_17_O_4_: 273.1127 found: 273.1124.

**3,4,4′-trihydroxy-3-methoxy-dihydrochalcone (b7)**: purification (cyclohexane/ethyl acetate: 70/30) led to a white powder (75%). m.p. 128–130 °C, UV: λ_max_ (EtOH, nm) 280, ε (L·mol^−1^·cm^−1^) 15,611. FTIR (pure) ν (cm^−1^): 3484, 3356, 1651. ^1^H-NMR (300 MHz, Acetone-*d*_6_) δ: 7.60 (dd, *J* = 1.9 Hz, 8.3 Hz, 1H, H11), 7.56 (d, *J* = 1.7 Hz, 1H, H15), 6.90 (d, *J* = 8.2 Hz, 1H, H12), 6.76 (d, *J* = 1.7 Hz, 1H, H5), 6.72 (d, *J* = 8.0 Hz, 1H, H8), 6.60 (dd, *J* = 1.8 Hz, 8.0 Hz, 1H, H9), 3.90 (s, 3H, H16), 3.20 (t, *J* = 7.6 Hz, 2H, H2), 2.84 (t, *J* = 7.6 Hz, 2H, H3). ^13^C-NMR (75 MHz, Acetone-*d*_6_) δ: 197.9 (C11), 152.2 (C13), 148.3 (C14), 145.8 (C6), 144.0 (C7), 134.3 (C4), 130.6 (C10), 123.8 (C11), 120.5 (C9), 116.4 (C5), 116.0 (C8), 115.4 (C12), 111.6 (C15), 56.3 (C16), 40.7 (C2), 30.5 (C3). HRMS: *m*/*z* [M+H]^+^ calculated for C_16_H_17_O_5_: 289.1076 found: 289.1075.

**3′,4,4′-trihydroxy-3-methoxy-dihydrochalcone (b8)**: purification (cyclohexane/ethyl acetate: 70/30) led to a white powder (46%). m.p. 139–141 °C, UV: λ_max_ (EtOH, nm) 276, ε (L·mol^−1^·cm^−1^) 13,187. FTIR (pure) ν (cm^−1^): 3507, 3326, 1658. ^1^H-NMR (300 MHz, Acetone-*d*_6_) δ: 7.53 (d, *J* = 1.9 Hz, 1H, H15), 7.47 (dd, *J* = 2.0 Hz, 8.3 Hz, 1H, H11), 6.90 (d, *J* = 8.4 Hz, 1H, H12), 6.88 (d, *J* = 1.2 Hz, 1H, H5), 6.74 (d, *J* = 8.0 Hz, 1H, H8), 6.70 (dd, *J* = 1.4 Hz, 8.0 Hz, 1H, H9), 3.80 (s, 3H, H16), 3.20 (t, *J* = 7.6 Hz, 2H, H2), 2.89 (t, *J* = 7.6 Hz, 2H, H3). ^13^C-NMR (75 MHz, Acetone-*d*_6_) δ: 198.2 (C1), 150.8 (C12), 148.1 (C6), 145.7 (C14), 145.5 (C7), 133.9 (C4), 130.7 (C10), 122.5 (C11), 121.5 (C9), 115.6 (C12 and C8), 115.5 (C15), 112.8 (C5), 56.1 (C16), 40.7 (C2), 30.6 (C3). HRMS: *m*/*z* [M+H]^+^ calculated for C_16_H_17_O_5_: 289.1076 found: 289.1073.

**4,4′-dihydroxy-3′,5′-dimethoxy-dihydrochalcone (b9)**: purification (cyclohexane/ethyl acetate: 60/40) led to a white powder (47%). m.p. 149–150 °C, UV: λ_max_ (EtOH, nm) 300, ε (L·mol^−1^·cm^−1^) 19,279. FTIR (pure) ν (cm^−1^): 3372, 1650. ^1^H-NMR (300 MHz, Acetone-*d*_6_) δ: 7.34 (s, 2H, H9-9′), 7.11 (d, *J* = 8.4 Hz, 2H, H5-5′), 6.75 (d, *J* = 8.5 Hz, 2H, H6-6′), 3.89 (s, 6H, H12-12′), 3.25 (t, *J* = 7.6 Hz, 2H, H2), 2.90 (t, *J* = 7.6 Hz, 2H, H3). ^13^C-NMR (75 MHz, Acetone-*d*_6_) δ: 197.1 (C1), 155.6 (C7), 147.5 (C10-10′), 140.9 (C11), 132.4 (C4), 129.4 (C5-5′), 128.1 (C10), 115.1 (C6-6′), 105.9 (C9-9′), 55.8 (C12-12′), 39.8 (C2), 29.7 (C3). HRMS: *m*/*z* [M+H]^+^ calculated for C_17_H_19_O_5_: 303.1232 found: 303.1232.

**4,4′-dihydroxy-3,3′-dimethoxydihydrochalcone (b10)**: purification (cyclohexane/ethyl acetate: 80/20) led to a white powder (59%). m.p. 108–109 °C, UV: λ_max_ (EtOH, nm) 280, ε (L·mol^−1^·cm^−1^) 12,962. FTIR (pure) ν (cm^−1^): 3457, 3269, 1646. ^1^H-NMR (300 MHz, Acetone-*d*_6_) δ: 7.60 (dd, *J* = 2.0 Hz, 8.2 Hz, 1H, H11), 7.55 (d, *J* = 2.0 Hz, 1H, H15), 6.90 (s, 1H, H5), 6.89 (d, *J* = 8.2 Hz, 1H, H12), 6.72 (m, 2H, H8 and H9), 3.90 (s, 3H, H17), 3.82 (s, 3H, H16), 3.24 (t, *J* = 7.6 Hz, 2H, H2), 2.90 (t, *J* = 7.7 Hz, 2H, H3). ^13^C-NMR (75 MHz, Acetone-*d*_6_) δ: 197.9 (C1), 152.2 (C13), 148.3 (C14), 148.2 (C6), 145.6 (C7), 133.9 (C4), 130.5 (C10), 123.8 (C11), 121.6 (C9), 115.6 (C8), 115.4 (C12), 112.9 (C5), 111.5 (C15), 56.3 (C17), 56.2 (C16), 40.7 (C2), 30.8 (C3). HRMS: *m*/*z* [M+H]^+^ calculated for C_17_H_19_O_5_: 303.1232 found: 303.1231.

**4,4′-dihydroxy-3,3′,5-trimethoxy-dihydrochalcone (b11)**: purification (cyclohexane/ethyl acetate: 60/40) led to a white powder (59%). m.p. 127–128 °C, UV: λ_max_ (EtOH, nm) 276, ε (L·mol^−1^·cm^−1^) 13,271. FTIR (pure) ν (cm^−1^): 3538, 3451, 1673. ^1^H-NMR (300 MHz, Acetone-*d*_6_) δ: 7.60 (dd, *J* = 2.0 Hz, 8.3 Hz, 1H, H9), 7.55 (d, *J* = 2.0 Hz, 1H, H13), 6.90 (d, *J* = 8.6 Hz, 1H, H10), 6.58 (s, 2H, H5-5′), 3.90 (s, 3H, H15), 3.79 (s, 6H, H14-14′), 3.25 (t, *J* = 7.7 Hz, 2H, H2), 2.90 (t, *J* = 7.7 Hz, 2H, H3). ^13^C-NMR (75 MHz, Acetone-*d*_6_) δ: 198.0 (C1), 152.2 (C11), 148.6 (C6-6′), 148.3 (C12), 135.1 (C7), 132.9 (C4), 130.6 (C8), 123.8 (C9), 115.4 (C10), 111.6 (C13), 106.8 (C5-5′), 56.6 (C14-14′), 56.3 (C15), 40.8 (C2), 31.4 (C3). HRMS: *m*/*z* [M+H]^+^ calculated for C_18_H_21_O_6_: 333.1338 found: 333.1334.

**4,4′-dihydroxy-3,3′,5,5′-tetramethoxy-dihydrochalcone (b12)**: purification (cyclohexane/ethyl acetate: 60/40) led to a light brown powder (38%). m.p. 168–172 °C, UV: λ_max_ (EtOH, nm) 302, ε (L·mol^−1^·cm^−1^) 12,072. FTIR (pure) ν (cm^−1^): 3323, 1646. ^1^H-NMR (300 MHz, Acetone-*d*_6_) δ: 7.32 (s, 2H, H9-9′), 6.58 (s, 2H, H5-5′), 3.86 (s, 6H, H13-13′), 3.79 (s, 6H, H12-12′), 3.27 (t, *J* = 7.6 Hz, 2H, H2), 2.90 (t, *J* = 7.6 Hz, 2H, H3). ^13^C-NMR (75 MHz, Acetone-*d*_6_) δ: 198.1 (C1), 148.6 (C6-6′), 148.4 (C10-10′), 141.8 (C11), 135.1 (C7), 132.9 (C4), 129.0 (C8), 106.9 (C5-5′), 106.8 (C9-9′), 59.7 (C13-13′), 59.6 (C12-12′), 40.8 (C2), 31.4 (C3). HRMS: *m*/*z* [M+H]^+^ calculated for C_19_H_23_O_7_: 363.1444 found: 363.1446.

### 2.5. DPPH Assays

The radical scavenging activity of all compounds was tested through 2,2-diphenyl-1-picryhydrazyl (DPPH) assay which determines the EC_50_ values as previously described [42]. The test consists of adding 190 µL of DPPH solution (200 µM) in ethanol to 10 µL of potential antiradical molecule solution at different concentrations (from 800 to 12.5 µM). The reaction was carried out in 96-well microplates and the disappearance of DPPH radicals was monitored at 520 nm every 5 min during 7.5 h in a multiplate Biotek (Winooski, VT, USA), Epoch 2 spectrophotometer. The EC_50_ values, corresponding to the concentration needed to reduce half of the initial population of DPPH radicals, were provided by the crossing point of the curves of %DPPH and %reduced DPPH. Curves dots were obtained using an average of the last six measurements for each concentration in Regressi^®^ software version 3.99.

### 2.6. Anti-Tyrosinase Assays

Tyrosinase inhibition activity was evaluated using the method used by Peyrot et al. [43]. In a 96-well microplate, 60 µL of ammonium formate buffer (50 mM, pH 6.4) were mixed with 10 µL of inhibitor solution at different concentrations: 10,000, 5000, 1000, 500, 100, 50, 10, 5, 1 and 0.5 µM in DMSO. Then, 20 µL of tyrosine at 4.42 mM in ammonium formate buffer was added. After 10 s of shaking, 10 µL of mushroom tyrosinase (5000 U.mL^−1^ in ammonium formate buffer) were added and the mixture was incubated for 10 min at 37 °C. The amount of dopachrome produced during incubation was determined by absorbance readings at 420 nm every 15 s in a microplate Biotek (Winooski, VT, USA), Epoch 2 spectrophotometer. IC_50_ values, corresponding to the concentration required to inhibit 50% of tyrosinase activity, were obtained using GraphPad Prism^®^ software version 6.01. Kojic acid was used as a reference and all measurements were performed at least in duplicate.

### 2.7. UV Analysis and Photostability

UV-Vis spectra were recorded at 10 µM in ethanol on a Cary 60 UV-Vis spectrophotometer by Agilent (Wilmington, DE, USA) in a 1 cm quartz cuvette. The absorbance loss was evaluated by comparison of absorbances at the λ_max_ before and after 1 h of irradiation into a Rayonet^®^ RPR-200 (λ = 300 nm, P = 8.32 W.m^−2^, stirring, T = 35 °C) (SNE Ultraviolet Co. Branford, CT, USA) using 14 RPR-3000A lamps.

## 3. Results and Discussion

### 3.1. Synthesis of Chalcones and Dihydrochalcones

CHs substituted by phenolic or aromatic methoxy moieties are common in nature and exhibit numerous biological activities [44]. Therefore, we first oriented our choice towards the synthesis of CHs substituted by OH in *ortho* position on the A ring as is the case for phloretin. However, in accordance with previous studies and our tests, under acid conditions, the formation of the cyclized forms is largely favored. Indeed, in the absence of protection, *ortho*-OH readily reacted with the double bond of the α,β-unsaturated ketone system of the CH resulting from the condensation between the aldehyde and the ketone [45]. Given these preliminary results and our desire to offer a green synthetic pathway, ketones with OH in *ortho* position were ruled out. The same observation was done with aldehydes substituted with OH in *ortho,* and so they were also ruled out [46].

We then explored the synthesis of CHs substituted by OH in *para*-position on A-ring and B-ring. Indeed, such a substituent in *para* has already demonstrated diverse biological activities for other molecules [47,48]. Furthermore, our preliminary results seemed to show that *para*-OH promoted the synthesis by increasing electron delocalization and thus making the aldehyde more electrophilic [49]. On the contrary, it was observed that a single OH in *meta* position on aldehydes or ketones adversely affects CHs synthesis. Indeed, these OH does not allow the delocalization of electrons to the ketone and aldehyde moieties, thus limiting their reactivity. Nevertheless, an additional OH in the *para* position allowed the reaction.

**Scheme 2:**
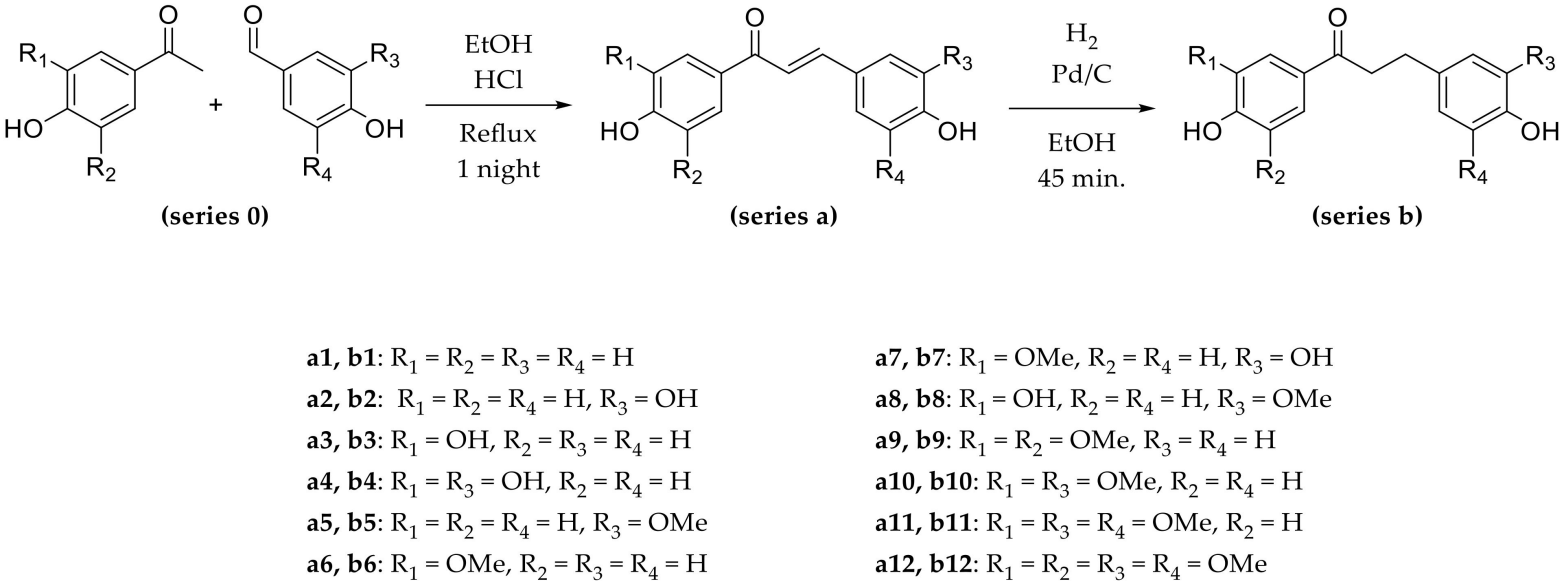
Two-step synthesis of chalcones and dihydrochalcones.

Considering the aforementioned observations, in order to offer biobased products and the greenest possible synthetic pathway, we oriented our choice towards aldehydes and ketones substituted by OH in *para*-position and simple substituents (-OH or -OCH_3_) in *meta*-position (Scheme 2, series 0). This way, the ketones chosen were piceol, 3,4-dihydroxyacetopehnone (i.e., acetopyrocatechol), acetovanillone, and acetosyringone, and aldehydes chosen were 4-hydroxybenzaldehyde, 3,4-dihydroxybenzaldehyde (i.e., protocatechualdehyde), vanillin and syringaldehyde (Scheme 2, series 0).

Various combinations of these ketones and aldehydes were performed through a green synthesis using ethanol as solvent and a catalytic amount of HCl to initiate the Claisen–Schmidt reaction. They led to 12 CHs (**a1** to **a12**) in yields between 47% and 80% after purification (Scheme 2, series a).

As previously mentioned, OH groups in *para* position promoted the condensation, in particular when they were on the ketone. In fact, the best yields were obtained with the *p*-hydroxyacetophenone (**a2**, **a5**: 80%, **a1**: 76%). *p*-Hydroxybenzaldehyde also led to significant yields **(a6**: 78%, **a3**: 68%, **a9**: 65%). Moreover, the use of vanillin was conducive to excellent yields (**a5**: 80%, **a8**: 78% and **a10**: 69%), while an additional -OCH_3_ in *meta* position (syringol groups) resulted in lower yields (**a12**: 52%, **a11**: 47%).

**Scheme 3:**
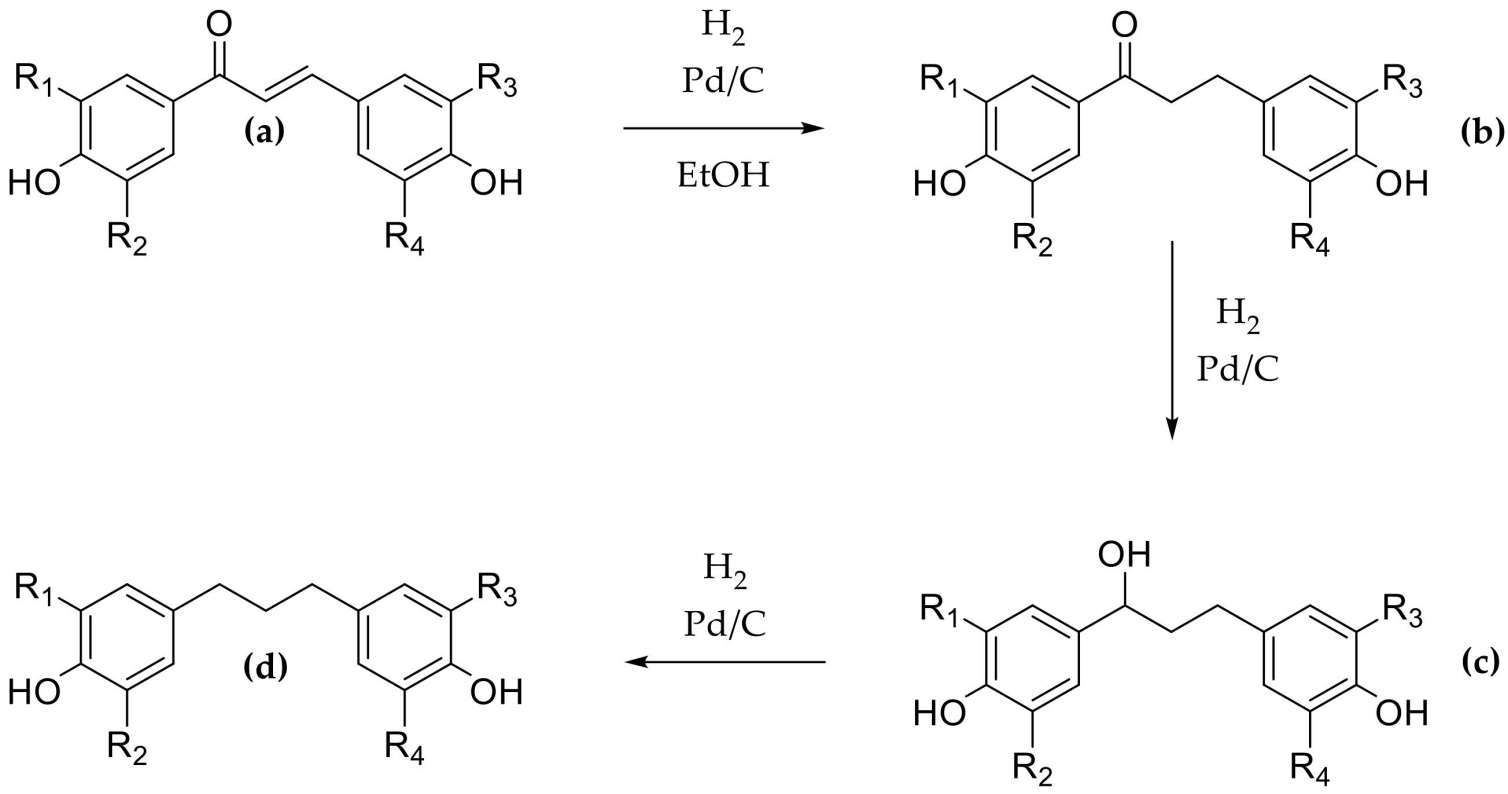
Hydrogenation reaction steps ((**a**) chalcone, (**b**) dihydrochalcone, (**c**) alcohol intermediate, and (**d**) fully hydrogenated compound).

Then DHCs were prepared by hydrogenation of the corresponding CHs using ethanol as solvent and palladium on carbon as catalyst. The reaction was performed for 45 min and led to the twelve DHCs **b1** to **b12** (Scheme 2, series **b**) in moderate to good yields between 38% and 75% after purification. Two other compounds were observed during each reaction that were identified by ^1^H-NMR as the alcohol intermediate (Scheme 3, compound **c**) and the fully hydrogenated compound (Scheme 3, compound **d**). The hydrogenation kinetics were monitored in order to minimize the formation of these two compounds and showed that the optimal time was 45 min. Moreover, polar solvent, such as ethanol, being usually used since they speed up hydrogenation [50], assays were performed using ethyl acetate to slow down the hydrogenation and limit the formation of compounds **c** and **d**. Unfortunately, the results were similar, and ethanol was preferred due to its greener character.

Given the obtained yields, the presence of catechol moiety on B-ring seemed to improve hydrogenation and provide better yields (**b7**: 75%, **b2**, **b4**: 69%). Then, good yields were observed when CH was only 4′-OH substituted (**b2**: 69%, **b5**: 64%, **b1**: 61%). Once again, the syringol groups on CHs limited their hydrogenation and led to poor yields of 47% and 38% for **b9** and **b12**, respectively.

Thanks to this simple two-step synthesis, we constituted a library of 24 molecules presenting various substitutions allowing different activities for the cosmetic sector. To the best of our knowledge, seven of them have never been described. All the chemical structures were confirmed by ^1^H-NMR, ^13^C-NRM (Spectra in Supplementary Materials) and HRMS analyses.

### 3.2. Antiradical Activities

All synthesized compounds, CHs and DHCs, were evaluated as potential antioxidants to be used in cosmetics through DPPH assay. Indeed, antioxidants are present in most of the formulations, and they protect the skin against damages from reactive oxygen species (ROS) but also stabilize formulations [51]. While ROS are naturally produced by biochemical reactions in human skin surface or from environmental stimulation, they can be harmful in high concentrations by inducing irreversible cellular damages. They result from molecular oxygen reduction and can lead to oxidative stress, premature skin aging or carcinogenesis [52]. Currently, two petro-sourced compounds are commonly used as antiradical agents, BHA (butylated hydroxyanisol) and BHT (butylated hydroxytoluene) but they are more and more criticized and suspected to be endocrine disruptor and carcinogenic [53,54]. Therefore, they need to be quickly replaced by natural, biobased and safe alternatives.

**Figure 2:**
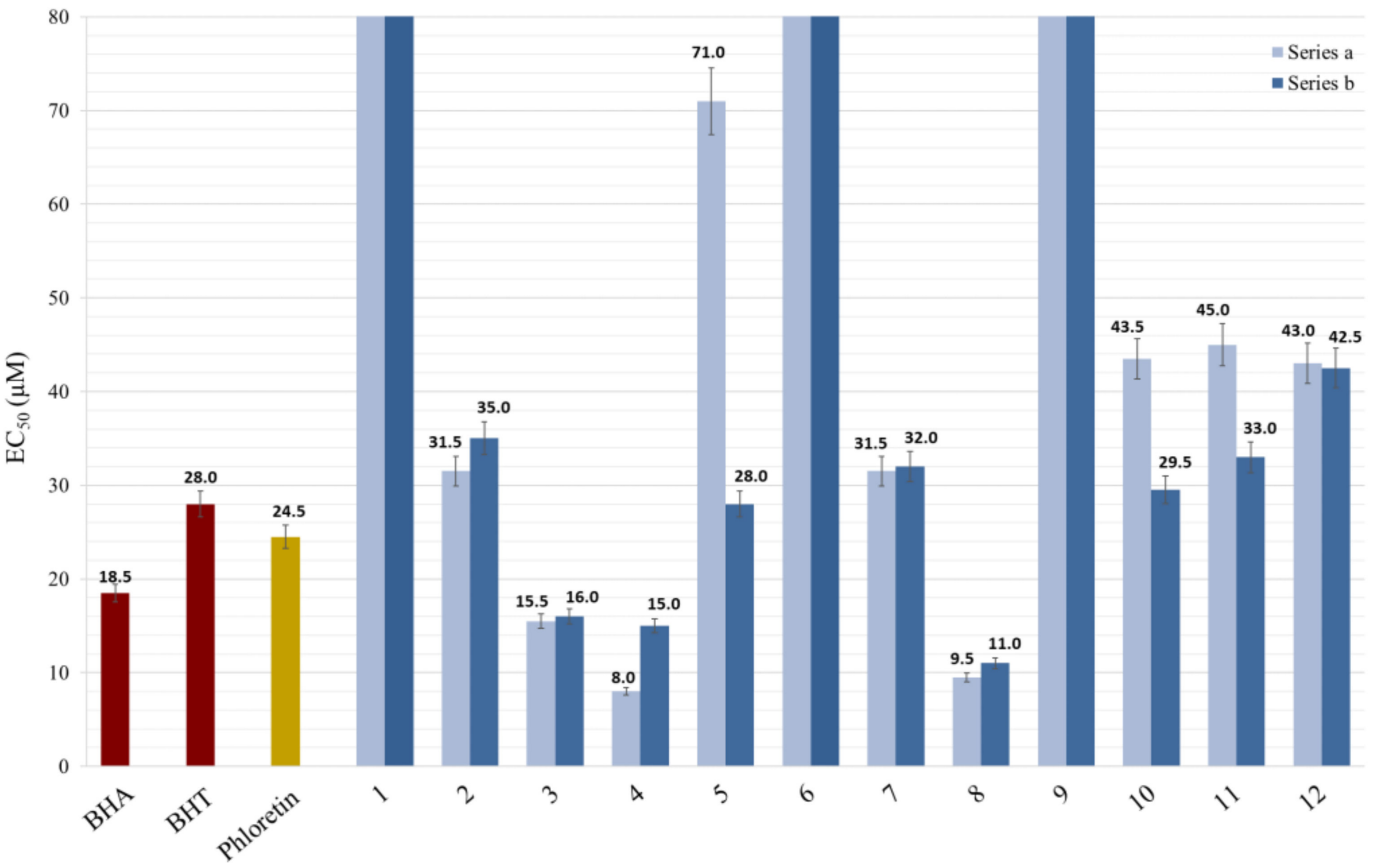
EC_50_ values (in µM) of synthetized molecules for DPPH inhibition in ethanol.

To evaluate the CHs and DHCs potential as radical scavengers, their EC_50_ values were determined and compared with that of BHA, BHT and phloretin. The lower the EC_50_, the better the antiradical activity was. Results are summarized in Figure 2.

As expected, phloretin exhibited a great antioxidant activity (EC_50_ = 24.5 µM), lower than BHA (EC_50_ = 18.5 µM), but competitive regarding BHT (EC_50_ = 28 µM).

From a global point of view, not all compounds showed antioxidant activity: **a1**, **a6** and their corresponding DHCs (**b1**, **b6**), as well as **a9** and **b9**, exhibited EC_50_ higher than 80 µM, which means an absence of activity due to the presence of only one hydroxy group on the B-ring in *para*-position. These results were expected. Indeed, compounds with similar structures, such as *p*-coumaric acid and derivatives, do not present antiradical activity [42,55]. However, **a3** and **b3,** which were also B-ring-substituted by only one hydroxy group, showed very competitive EC_50_ values regarding that of phloretin (15.5 and 16.0 µM, respectively). This difference was clearly due to the catechol moiety present at the A-ring level, well described for its antioxidant activity. This observation was confirmed by the low EC_50_ of other compounds with these patterns (**a4**, **b4**, **a8** and **b8**) presenting values of 8, 15, 9.5 and 11 µM, respectively. Catechol moiety on B-ring also provided great antiradical activities as observed for compounds **a2**, **b2**, **a7** and **b7,** which showed interesting values ranging between 31 and 35 µM. These groups were strongly involved in the antiradical activity, whether they were on A- or B-ring, as has been already demonstrated by Kozlowski et al. [56]. These authors also highlighted the guaiacol moiety contribution (i.e., OH and OCH_3_ groups in *ortho* configuration), especially on the B-ring, which confirmed our observations for compounds **a5**, **b5**, **a10** and **b10**. B-ring syringol moieties seemed to also influence the antioxidant activity, but to a lesser extent, as evidenced by the EC_50_ values of the compounds **a11**, **b11**, **a12** and **b12.** As was already observed by Mouterde et al., by comparing the wholeness of EC_50_ values, an additional hydroxy group impacted more strongly the antioxidant activity than a methoxy [57].

Finally, contrary to what we expected, some DHCs (series b) showed better antioxidant activity than their corresponding CHs (**b5**, **b10** and **b11**), even though it was previously observed that the α,β-double bond is important for the activity [56]. This phenomenon was probably due to the presence of a common guaiacol moiety.

Overall, for both series, the most effective molecules were substituted by catechol groups, and their EC_50_ values were highly competitive with the currently used references and phloretin. These results may be confirmed in vivo.

### 3.3. Anti-Tyrosinase Activities

Tyrosinase is the key enzyme of skin and hair pigments biosynthesis. Indeed, it intervenes in the two first steps of melanin production. First, it hydroxylates tyrosine into L-DOPA (3,4-dihydroxyphenylalanine); then, it oxidizes L-DOPA into L-DOPAquinone. The latter spontaneously rearranges to form L-DOPAchrome. Melanin protects the skin against UV radiation, but overexposure to the sun can lead to abnormal production and thus to hyperpigmentation on exposed areas. Tyrosinase inhibition is one more common way to reduce pigment spots. For this reason, tyrosinase inhibitors are widely used as lightening agents in cosmetics.

Tests have been performed mimicking human tyrosinase activity using a fungal tyrosinase. Through these tests, the IC_50_ values of all the synthesized molecules were determined and compared to that of Kojic acid, which is used as a reference for in tubo tyrosinase inhibition [58]. The lower the IC_50_ value, the better the anti-tyrosinase effect is. Results are summarized in Table 1.

First, contrary to many studies [20,59], in our experimental conditions, phloretin did not exhibit tyrosinase inhibition activity against tyrosinase from mushrooms.

Concerning the synthesized molecules, five CHs showed lower IC_50_ values than Kojic acid (IC_50_ = 0.42 mM). In terms of structure–activity relationship, a *para*-OH substituent on B-ring led to higher tyrosinase inhibition. Indeed, the CHs **a1**, **a9** and **a6** presented the greater activity, with IC_50_ values of 0.1, 0.13 and 0.14 mM, respectively. Surprisingly, the compound **a3** did not show any activity, although it was also 4-OH-substituted. Moreover, a catechol group on B-ring also provided enzyme inhibition, such as **a2** and **a4**, which exhibited IC_50_ of 0.23 and 0.50 mM, respectively, competitive with kojic acid value. Finally, **a10**, **a11** and **a7**, substituted by a guaiacol group on A-ring, displayed a relatively poor inhibition, indicating that this moiety may decrease the bioactivity.

CHs were overall more efficient than DCHs. Indeed, only three DHCs, **b5**, **b8** and **b10,** revealed inhibition activity. However, their IC_50_ values were better than those of Kojic acid, and all had the same B-ring (guaiacol moiety).

Considering these observations, the tyrosinase inhibition activity appeared to be mainly related to the B-ring substitution according to the following order for CHs: hydroxy > catechol > guaiacol. Concerning DHCs, only guaiacol groups induced an activity. Syringol moiety did not show any tyrosinase inhibition. Although some compounds are very promising for cosmetics uses, these results should be confirmed subsequently by tests on cells.

### 3.4. UV Analysis and Molecules Photostability

Currently, the sunscreen market is characterized by a strong demand for new biobased UV filters, especially since petrosourced filters such as avobenzone and octinoxate are increasingly criticized due to their environmental and health impact [60,61,62]. It is well known that conjugated molecules can absorb ultraviolet wavelengths, so we undertook to examine the potential of our molecules for this application.

**Figure 3:**
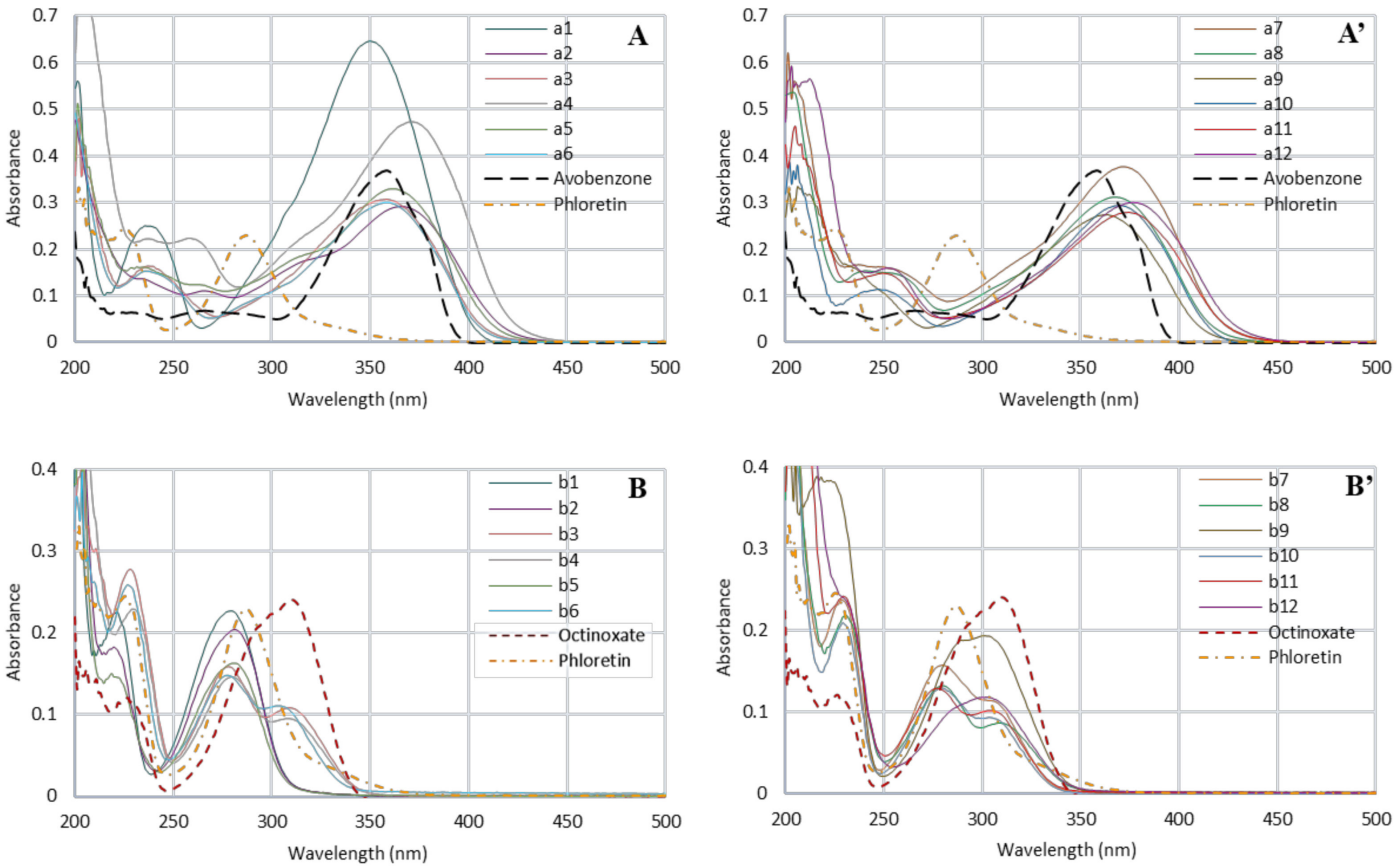
UV-Vis spectrum of chalcones series (**A**,**A’**), dihydrochalcones series (**B**,**B’**), phloretin (dotted orange line), avobenzone (dotted black line) and octinoxate (dotted red line) at 10 µM in ethanol. For each series, spectra have been split into two graphics and the scale has been adapted for clarity.

To this aim, we first evaluated UV properties by recording UV-Vis spectra at 10 µM in ethanol. Regarding the maximum absorption wavelengths obtained for CHs, **a1**–**a12** (350–393 nm), spectra were compared with avobenzone (UV-A reference, λ_max_= 357 nm) (Figure 3A,A’). Concerning DHCs **b1**–**b12** and phloretin, the maximum absorption wavelengths (278–302 nm) were preferentially compared with octinoxate (UV-B reference, λ_max_= 310 nm) (Figure 3B,B’).

CHs presented similar spectra with two major absorption bands. The first in the range 200–250 nm, generally noted as band II, was due to the A-ring benzoyl system. The second one, band I, in the range 340–400 nm originated from the B-ring cinnamoyl system [28] and was close to the avobenzone wavelength coverage (dotted black line, Figure 3A,A’). Only three compounds were competitive in regard to avobenzone (0.37): **a1**, **a4** and **a7** with absorbance values of 0.65, 0.47 and 0.38, respectively. Concerning other CHs, their absorbances were equivalent, around 0.29–0.30, for whom the lowest was 0.27 for compound **a11**, and thus were far from being insignificant.

Regarding the DHCs (i.e., phloretin and the twelve analogs), the presence of the two bands was also observed, although as expected, the absence of the double bond resulted in a hypsochromic shift of the band I at around 250–350 nm covering a part of UV-B like with octinoxate (dotted red line, Figure 3B,B’). DHCs absorbances were lower than those of CHs. However, phloretin showed a maximal absorbance (0.23), competitive with octinoxate (0.24), as did compound **b1** (0.23). Finally, **b2** and **b9** also revealed significant absorbances (0.21 and 0.19, respectively), while the other DHCs were not efficient enough. It may be noted that some compounds presented two absorption maxima for the band I (**b3**, **b4**, **b6**, **b7**, **b8**, and **b11**).

**Figure 4:**
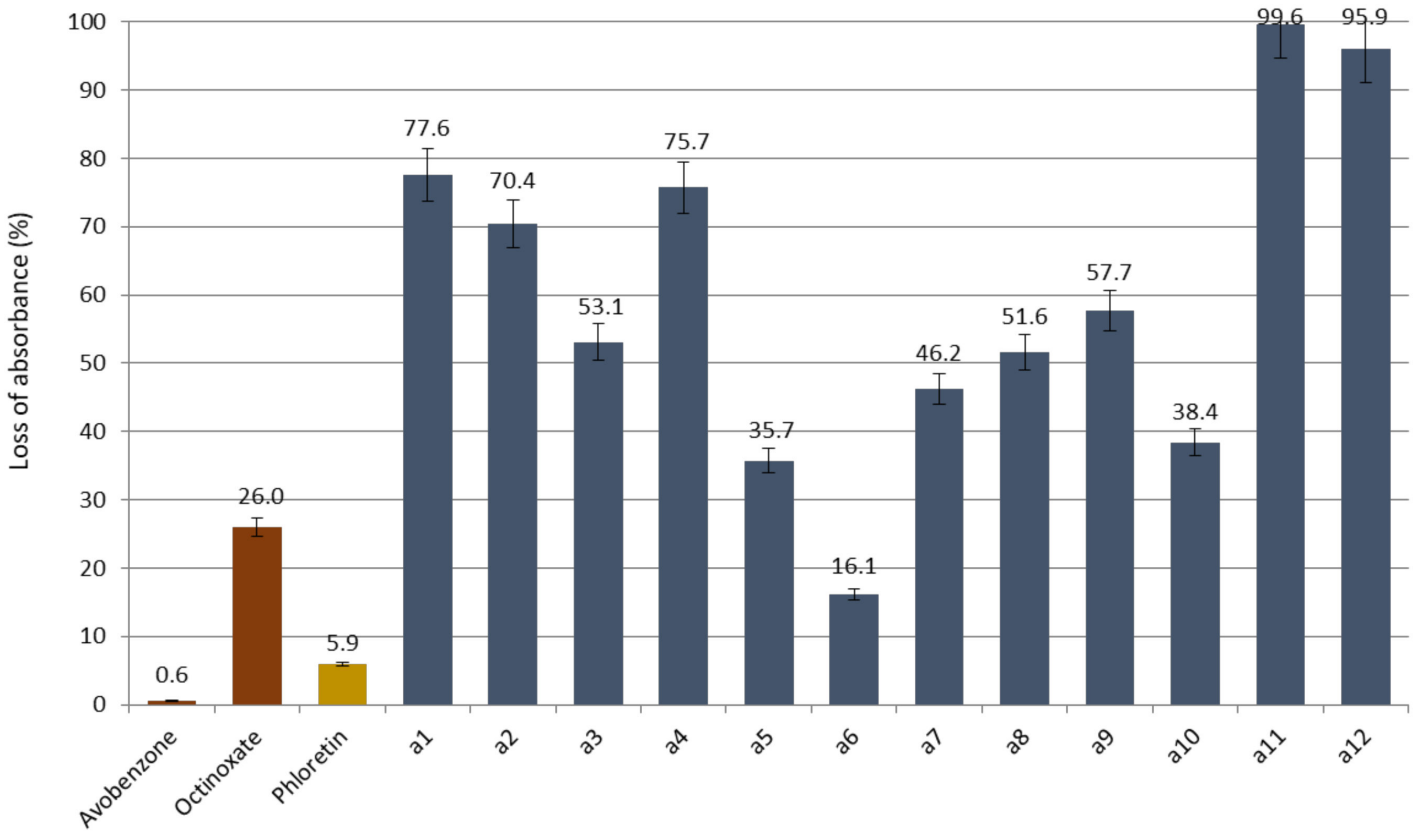
Loss of absorbance (in %) for chalcone series, phloretin and references avobenzone and octinoxate.

**Figure 5:**
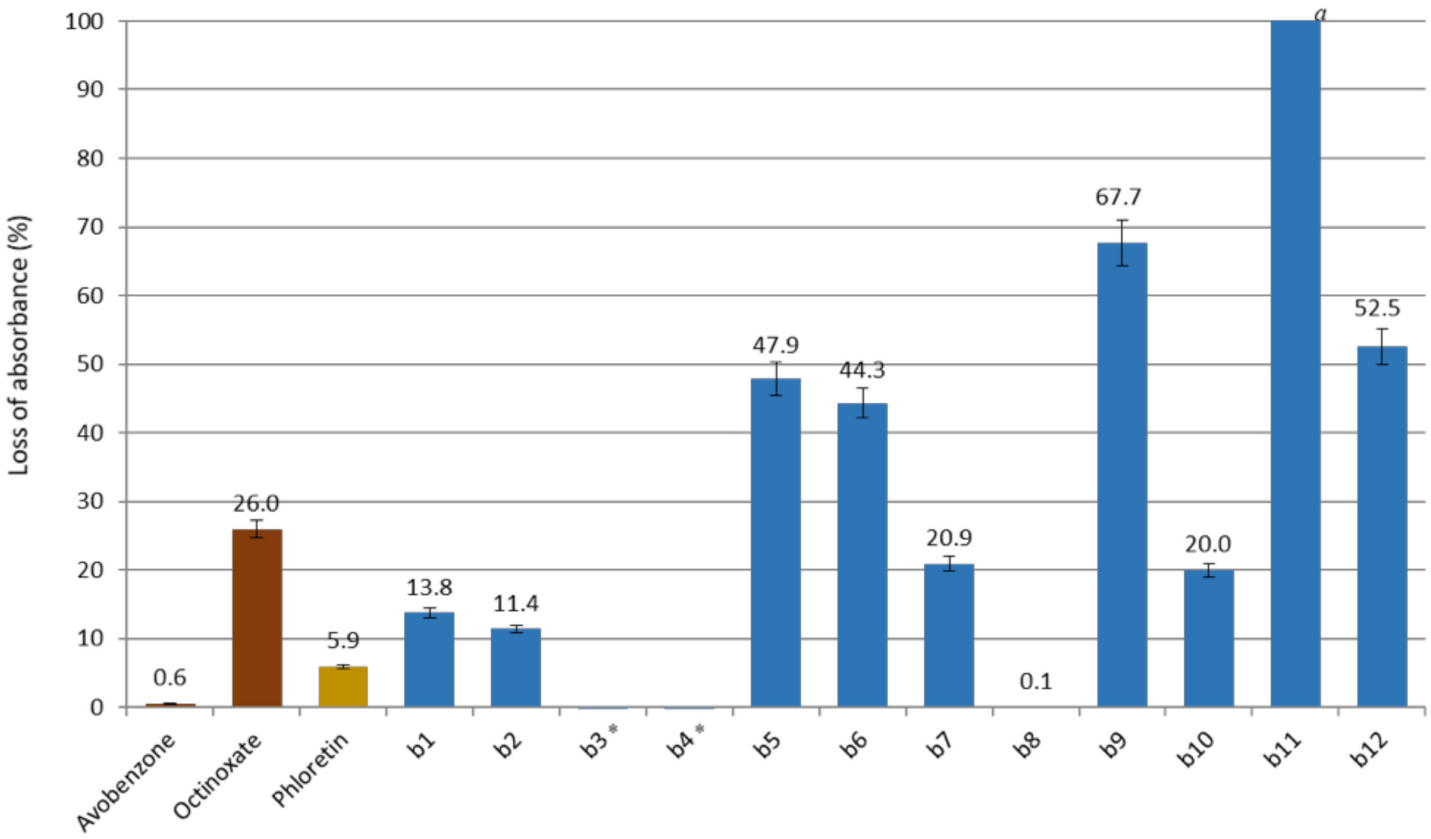
Loss of absorbance (in %) for dihydrochalcones series, phloretin and references avobenzone and octinoxate. ^a^ Full loss of absorbance. * Negative loss of absorbance (after 1 h of irradiation the absorbance was higher than at t0).

Studying wavelength coverage and absorbance intensity was not enough to evaluate the potential of CHs and DHCs as UV filters. Indeed, it is important to assess their photostability, correlated to their loss of absorbance upon UV irradiations. For this, compounds in ethanol were irradiated (10 µM, 1 h, 300 nm). UV-Vis spectra of the resulting solutions were recorded, and the maximal absorbance were compared to the non-irradiated solutions for each compound. CHs and phloretin absorbance losses are presented in Figure 4 and those of DHCs in Figure 5 (Spectra in Supplementary Materials).

First, concerning phloretin, its loss of absorbance was 5.9% against 26.0% for octinoxate and only 0.6% for avobenzone. Peyrot et al. mentioned in their study that the acceptable absorbance loss to match the commercial UV filters specifications should be less than 5% [43]. Phloretin’s result was very close to these specifications and revealed itself more photostable than octinoxate. Thus, this natural compound constitutes an alternative to petrosourced UV-B filters.

Regarding the synthesized CHs, promising compounds in terms of absorption intensity, **a1**, **a4** and **a7** showed excessively high absorbance losses of 77.6%, 75.7% and 46.2%, respectively. Only **a6** displayed a loss of absorbance of 16.1% that could compete with UV-A filters.

It is noteworthy to mention that, unexpectedly, DHCs **b3** and **b4** showed a higher absorbance higher after 1 h of irradiation. Indeed, for these compounds, the exposure to UV induced the disappearance of the second maximum absorption and the increase of the first (see spectra in Supplementary Materials). This was also the case for **b8**, which presented a very poor loss of absorbance of 0.1%. However, it was not the case for all compounds having two maximum absorption at t0, such as **b6** and **b11**. There does not seem to be any correlation between the structure of the molecule and the presence of these two maximum absorbances. Nonetheless, compounds **b3**, **b4** and **b8** were all A-ring-substituted by a catechol group. A catechol B-ring substitution also provided a relatively low loss of absorbance, as shown by **b2** and **b7** data, which were lower than those of octinoxate.

Unfortunately, **b9**, which was far from being insignificant in terms of absorbance, showed after irradiation 67.7% of loss of absorbance. On the contrary, **b10**, presenting a low loss of absorbance (20%), had a maximum absorbance at t0 of 0.13, too low to compete with the current UV filters.

Finally, **b1** and **b2** were the only two synthesized analogs potentially competing with octinoxate. Indeed, in addition to displaying a maximum absorbance close to that of the reference, they showed a relatively low loss of absorbance (13.8% and 11.4%, respectively).

To conclude, CHs appeared to be non-competitive as UV filters, especially compared to avobenzone which absorbs at the same wavelengths. Concerning DHCs, **b1** and **b2** appeared as potential alternatives to octinoxate. However, as with any in vitro test, these results remain to be confirmed in vivo.

## 4. Conclusions

In light of the high potential of phloretin in terms of biological activities and considering its low availability and its tedious and non-sustainable chemical synthesis, twenty-four analogs (chalcones and dihydrochalcones) were synthesized in good yields through a green HCl-catalyzed Claisen–Schmidt condensation between biobased ketones and aldehydes in ethanol followed by palladium-catalyzed hydrogenation. Antioxidant, anti-tyrosinase and UV filter properties were investigated for all compounds. Structure-activity relationships revealed the importance of the number and positions of hydroxyl substituents. This study confirmed the potent activity of catechol groups on CHs and DHCs. Finally, even if only two analogs (**b1** and **b2**) showed interesting anti-UV properties, several compounds exhibited promising antioxidant and/or anti-tyrosinase activity, especially **a4** and **b8**, which appeared as competitive biobased antioxidants and tyrosinase inhibitors and thus sustainable alternatives to petrosourced counterparts. However, they must be more investigated further before a potential application in cosmetics; the results of these investigations will be reported in due course.

## Figures and Tables

**Table 1:** IC_50_ of synthesized compounds for mushroom tyrosinase inhibition.

Series	Compound	IC_50_ (mM)
Chalcones series	**a1**	0.10 ± 0.01
	**a2**	0.50 ± 0.03
	**a3**	- ^a^
	**a4**	0.23 ± 0.01
	**a5**	2.3 ± 0.1
	**a6**	0.14 ± 0.01
	**a7**	2.3 ± 0.1
	**a8**	- ^a^
	**a9**	0.13 ± 0.01
	**a10**	1.80 ± 0.09
	**a11**	7.9 ± 0.4
	**a12**	- ^a^
Dihydrochalcones series	**b1**	- ^a^
	**b2**	- ^a^
	**b3**	- ^a^
	**b4**	- ^a^
	**b5**	0.50 ± 0.03
	**b6**	- ^a^
	**b7**	- ^a^
	**b8**	0.07 ± 0.01
	**b9**	- ^a^
	**b10**	0.27 ± 0.01
	**b11**	- ^a^
	**b12**	- ^a^
References	**Phloretin**	- ^a^
	**Kojic acid**	0.42 ± 0.02

^a^ No activity observed.

## Data Availability

Data is contained within the article and supplementary material.

## Supplementary Materials

The following are available online at https://www.mdpi.com/article/10.3390/antiox10040512/s1, the ^1^H and ^13^C-NMR spectra, tyrosinase inhibitor assay, DPPH assay, UV spectrum and loss of absorbance study are provided for every single molecule synthesized in this work.

## Abbreviations

CH(s): chalcone(s); DHC(s), dihydrochalcone(s).
