# Supplementary material for: Synthesis of Biobased Phloretin Analogues: An Access to Antioxidant and Anti-Tyrosinase Compounds for Cosmetic Applications
Source: Antioxidants (Basel). 2021 Mar 25;10(4):512. doi: 10.3390/antiox10040512 (PMC8064475; doi:10.3390/antiox10040512)
Supplement: Supplementary file 1 [file antioxidants-10-00512-s001.pdf]

Synthesis of biobased phloretin analogues: an access to antioxidant and anti-tyrosinase compounds for cosmetic applications

Laurène Minsat[1,2], Cédric Peyrot[1]*, Fanny Brunissen[1], Jean-Hugues Renault[2], Florent Allais[1]*

[1]URD Agro-Biotechnologies Industrielles (ABI), CEBB, AgroParisTech, 51110, Pomacle, France
[2]Université de Reims Champagne-Ardenne, CNRS, ICMR 7312, 51097, Reims, France
*Corresponding Author: florent.allais@agroparistech.fr; cedric.peyrot@agroparistech.fr

Table of contents

1. **^{1}H & ^{13}C NMR spectra unsaturated molecules (series a)** 2
2. **^{1}H & ^{13}C NMR spectra saturated molecules (series b)** 14
3. **Tyrosinase inhibition** 26
4. **Antioxidant activity** 28
5. **UV Filter activity** 31
6. **FT-IR spectra** 34

1. ^{1}H & ^{13}C NMR spectra unsaturated molecules (series a)

a1

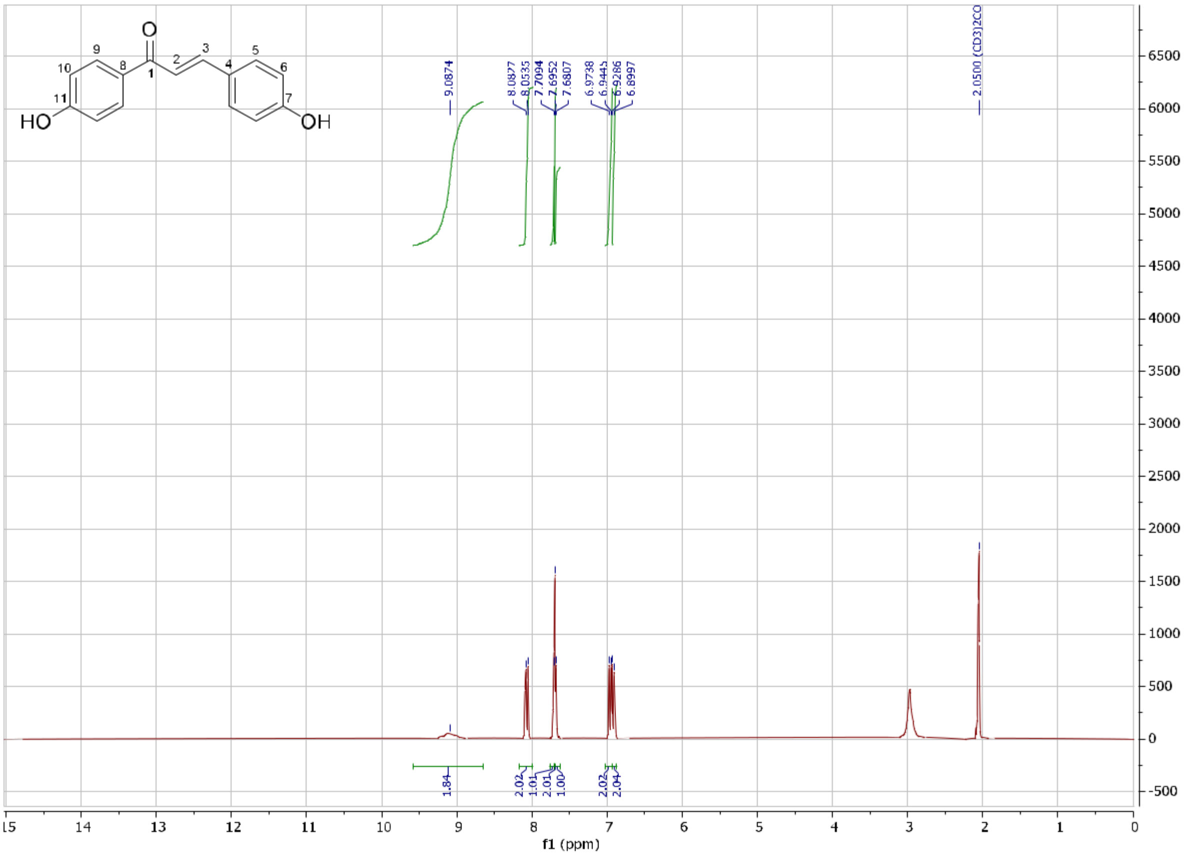

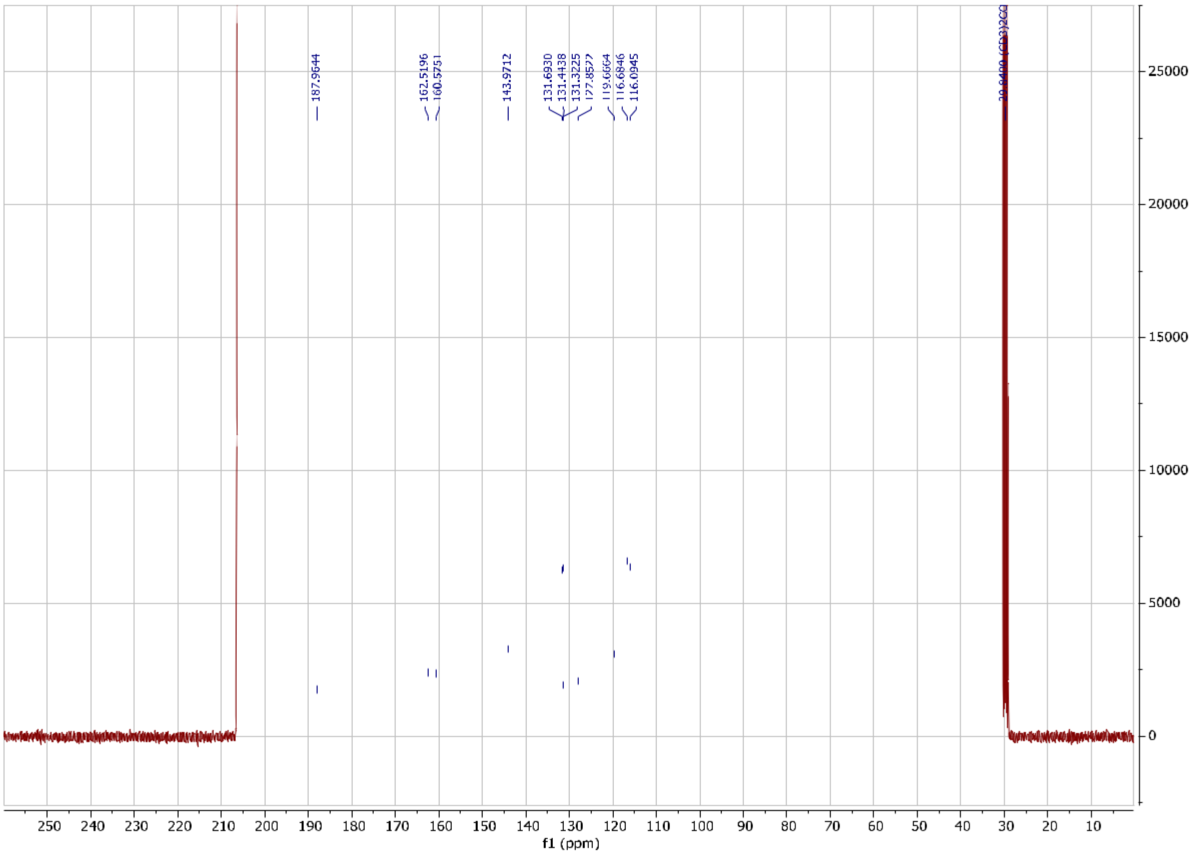

a2

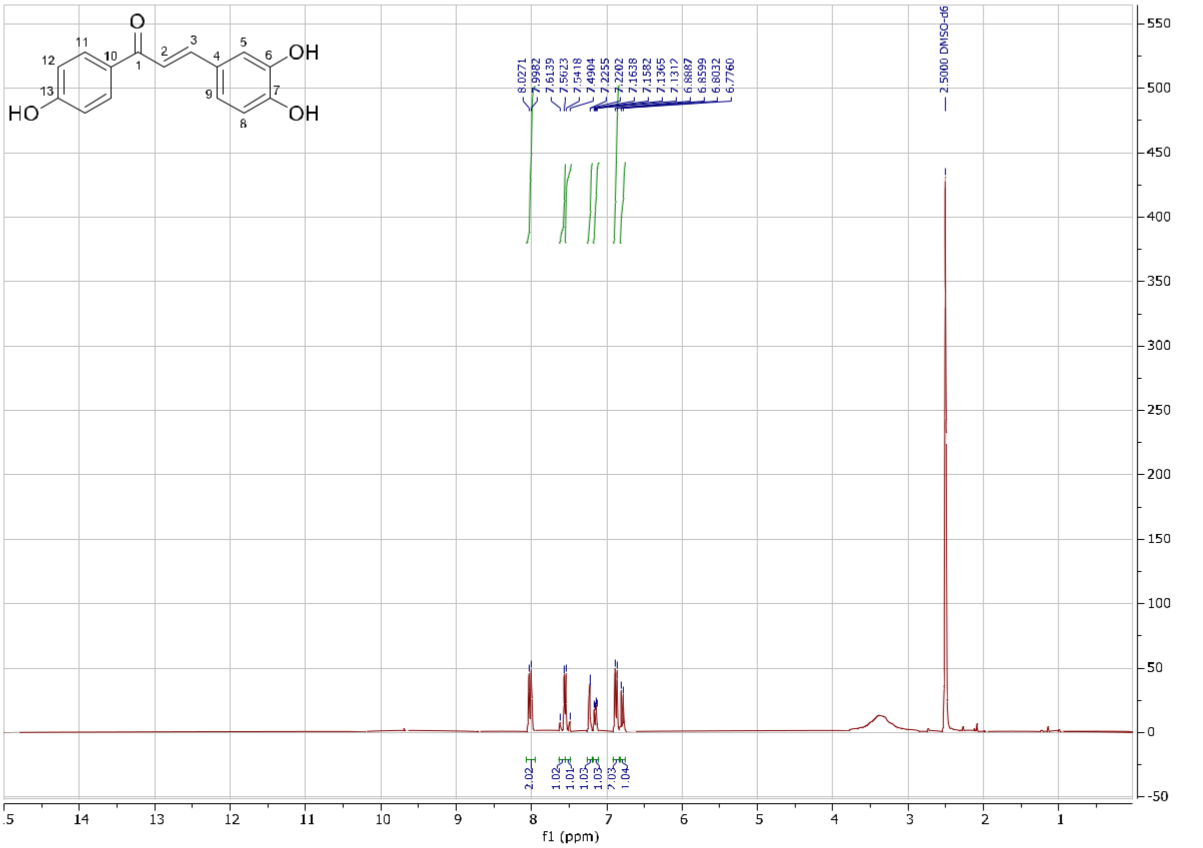

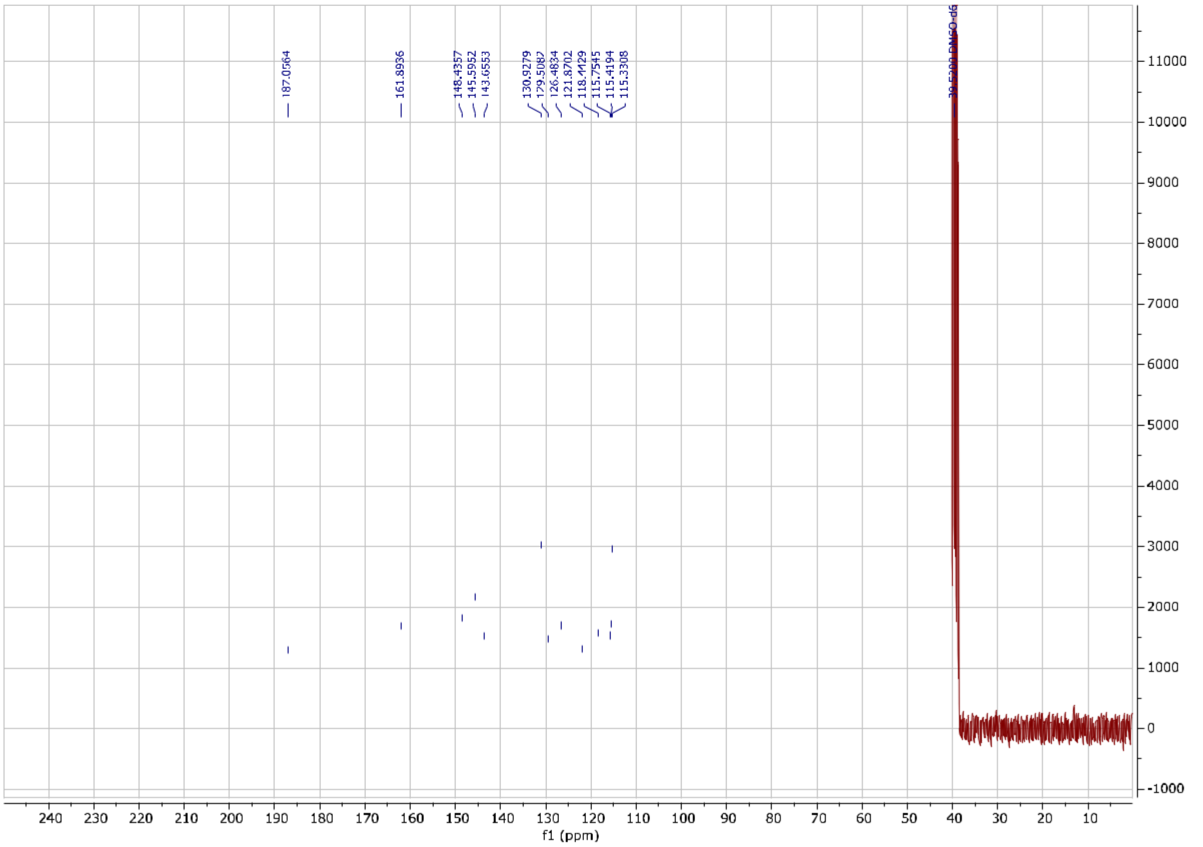

a3

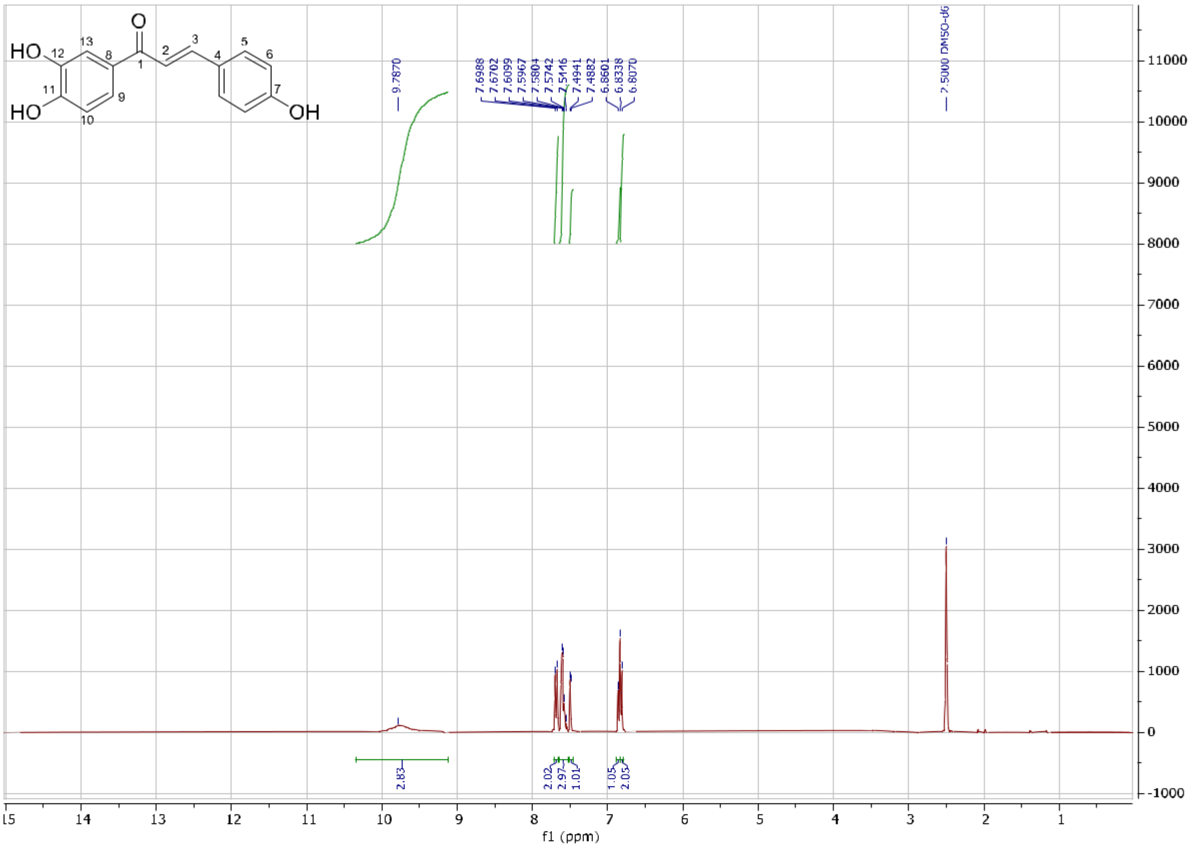

HO
HO
OH
O
9.7870
7.6988
7.6702
7.6099
7.5967
7.5804
7.5742
7.5446
7.4941
7.4882
6.8601
6.8338
6.8070
2.5000 DMSO-d6
2.83
2.02
2.97
1.01
1.05
2.03
f1 (ppm)

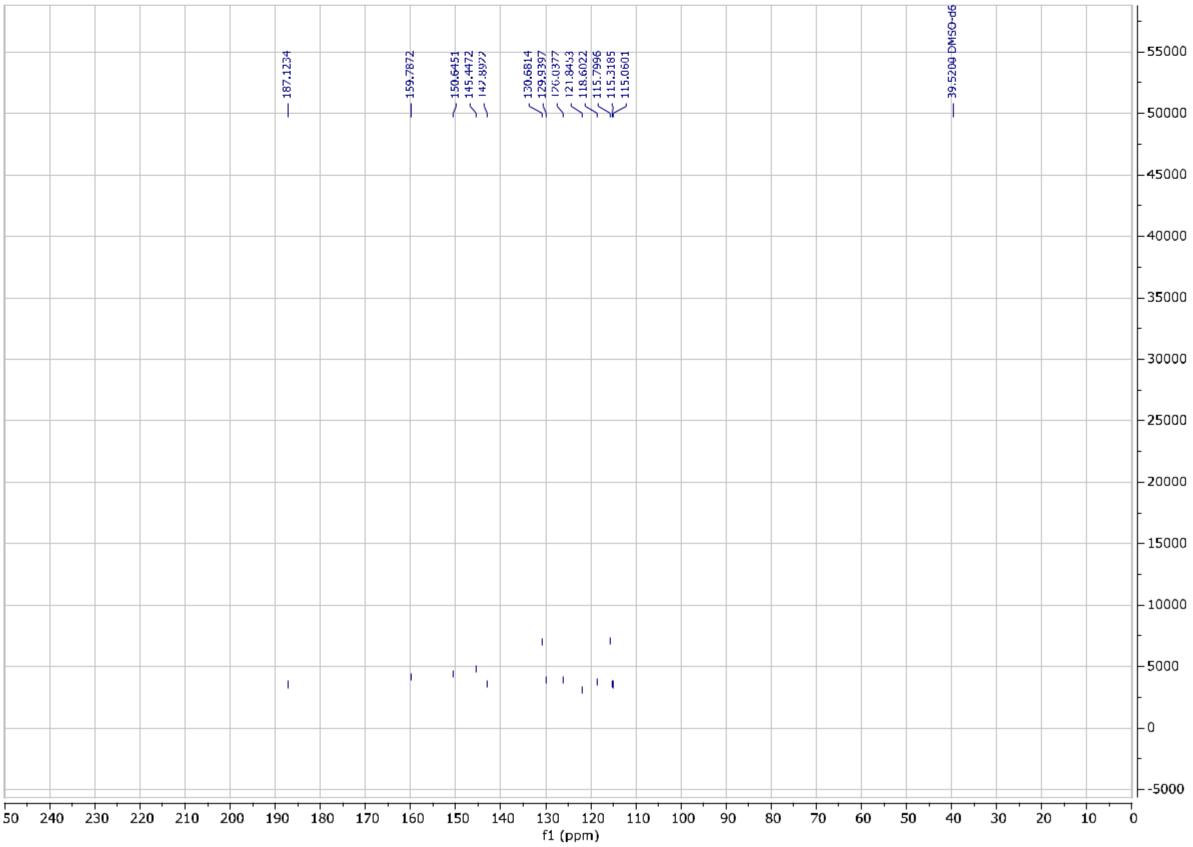

187.1234
159.7872
150.6451
145.4472
142.8922
130.6814
129.9397
126.0377
121.8453
118.6022
115.7996
115.3185
115.0601
39.5200 DMSO-d6
f1 (ppm)

a4

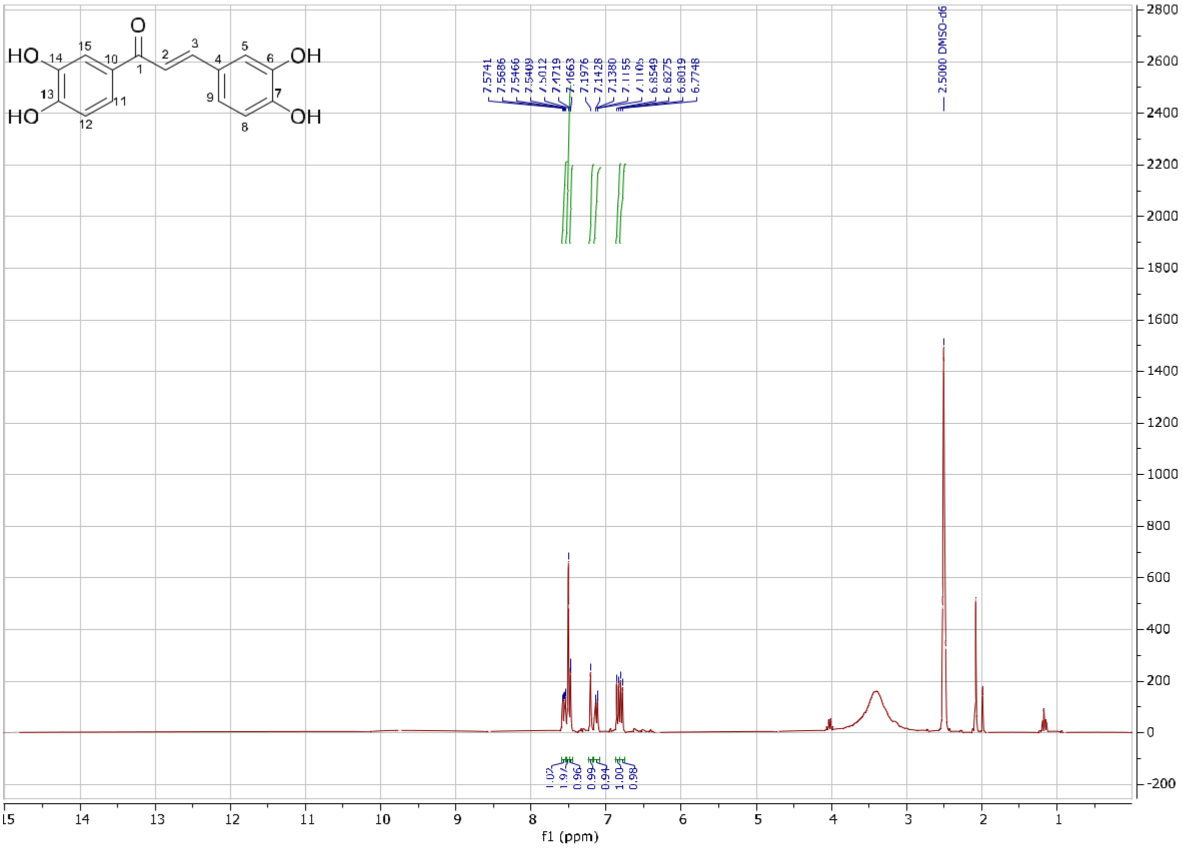
HO
HO
OH
OH
O
7.5741
7.5686
7.5466
7.5409
7.4719
7.4663
7.1976
7.1428
7.1380
6.8549
6.8275
6.8019
6.7748
2.5000 DMSO-d6
f1 (ppm)

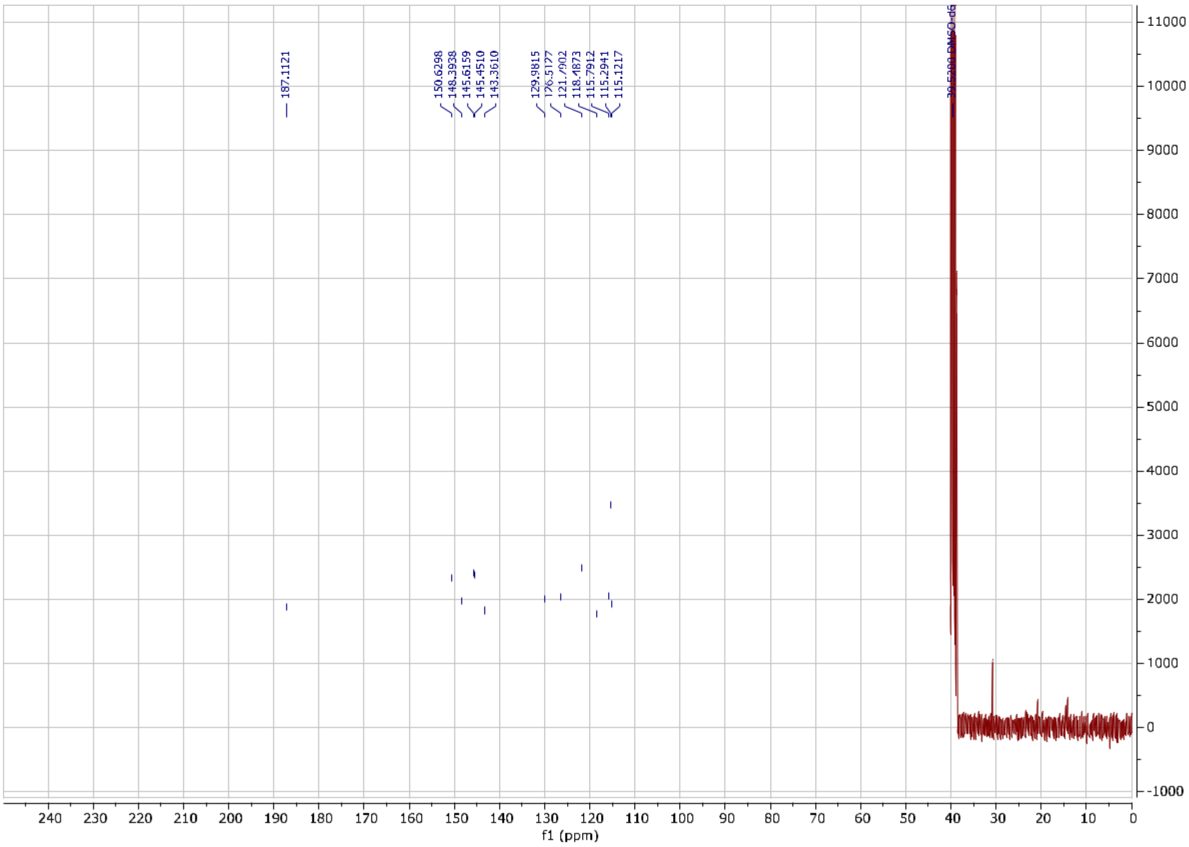
187.1121
150.6298
148.3938
145.6159
145.4510
143.3610
129.9815
126.5127
121.7902
118.4873
115.7912
115.2941
115.1217
f1 (ppm)

a5

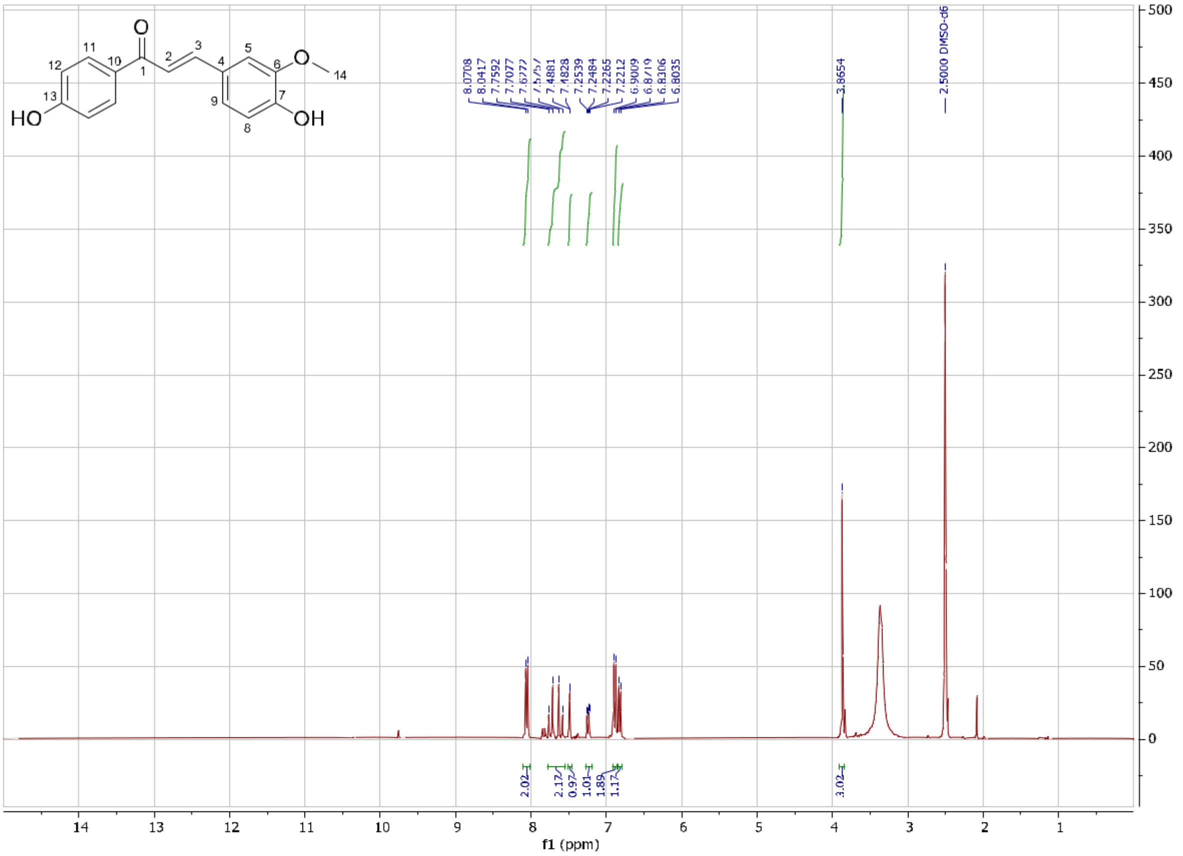

HO
OH
O
8.0708
8.0417
7.7592
7.7077
7.6272
7.4881
7.4828
7.2539
7.2484
7.2265
7.2212
6.9009
6.8719
6.8306
6.8035
3.8654
2.5000 DMSO-d6
2.02
2.17
0.92
1.01
1.89
1.17
3.02
f1 (ppm)

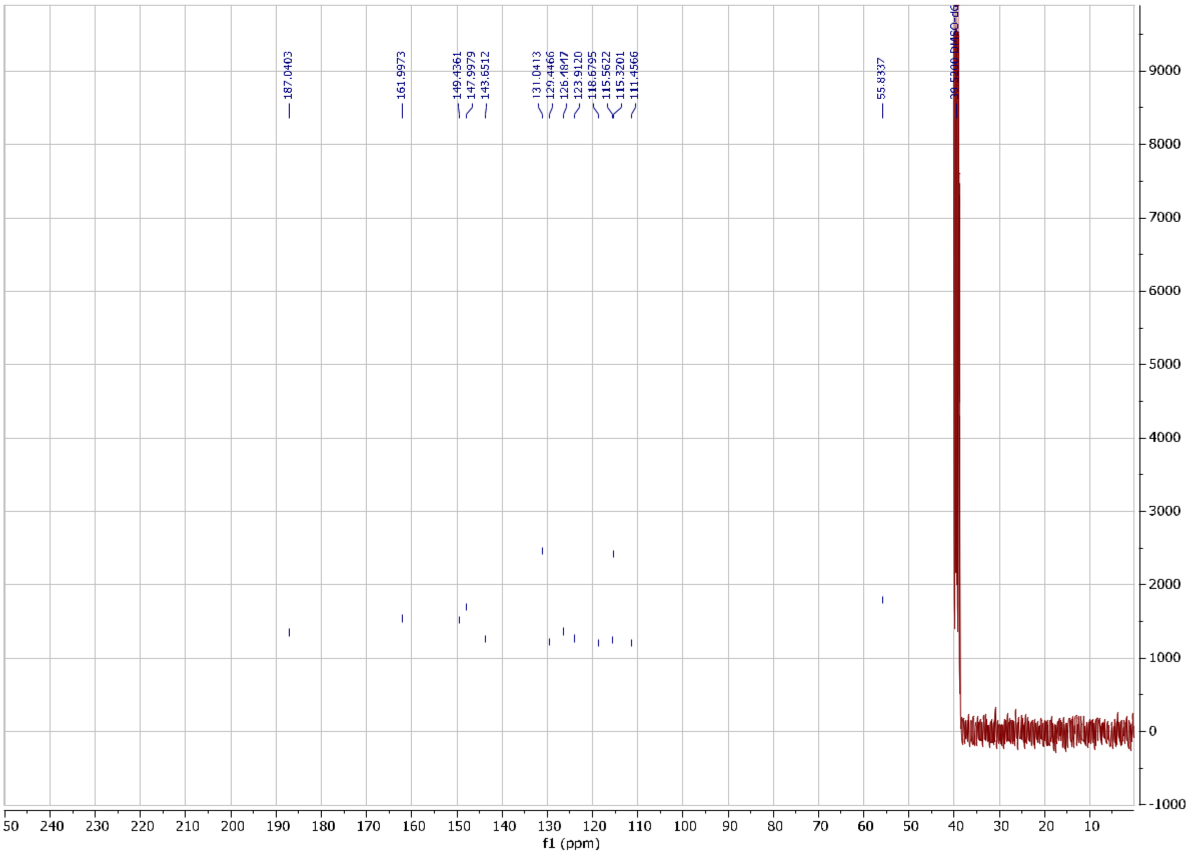

187.0403
161.9973
149.4361
147.9979
143.6512
131.0413
129.4466
126.4847
123.9120
118.6795
115.5622
115.3201
111.4566
55.8337
f1 (ppm)

a6

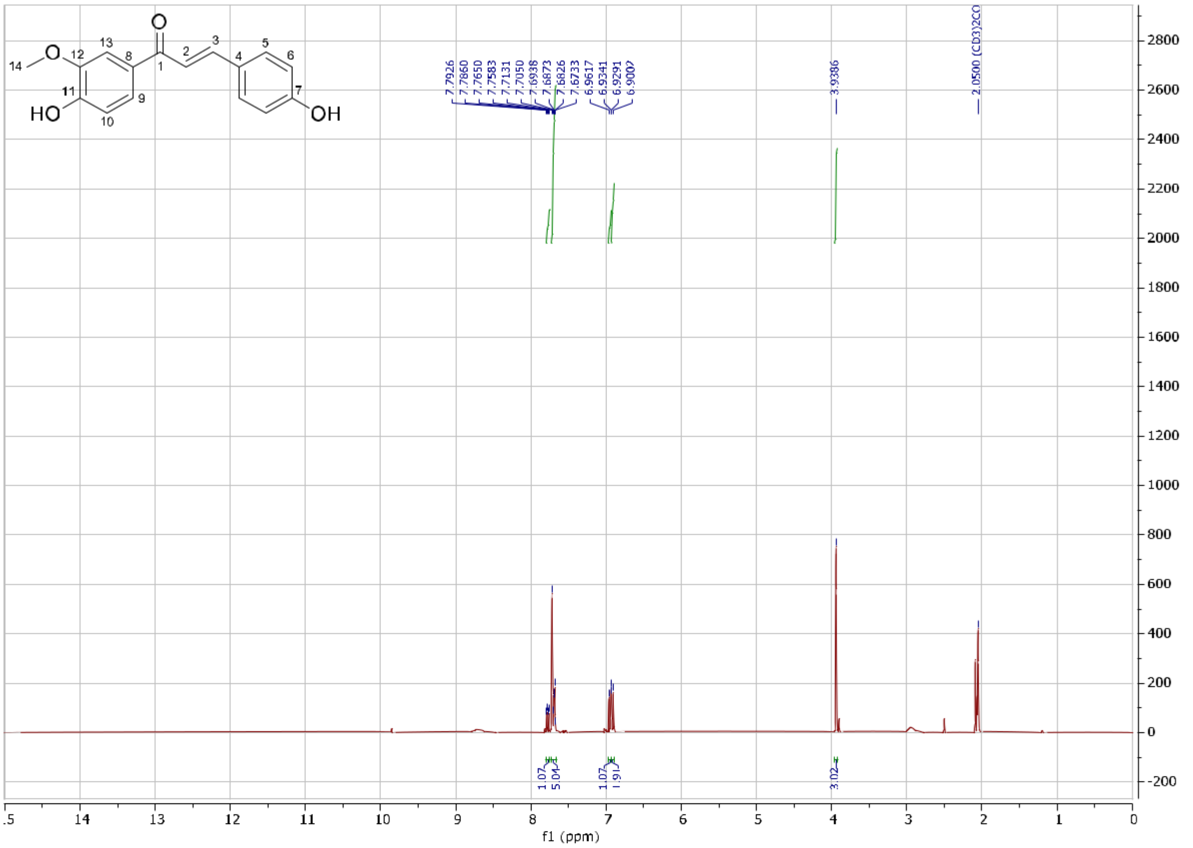

14
O
12
13
8
1
O
2
3
4
5
6
7
OH
11
HO
10
9
7.7926
7.7860
7.7650
7.7583
7.7131
7.7050
7.6938
7.6873
7.6826
7.6733
6.9617
6.9341
6.9291
6.9002
3.9386
2.0500 (CD3)2CO
1.07
5.04
1.07
1.91
3.02
f1 (ppm)

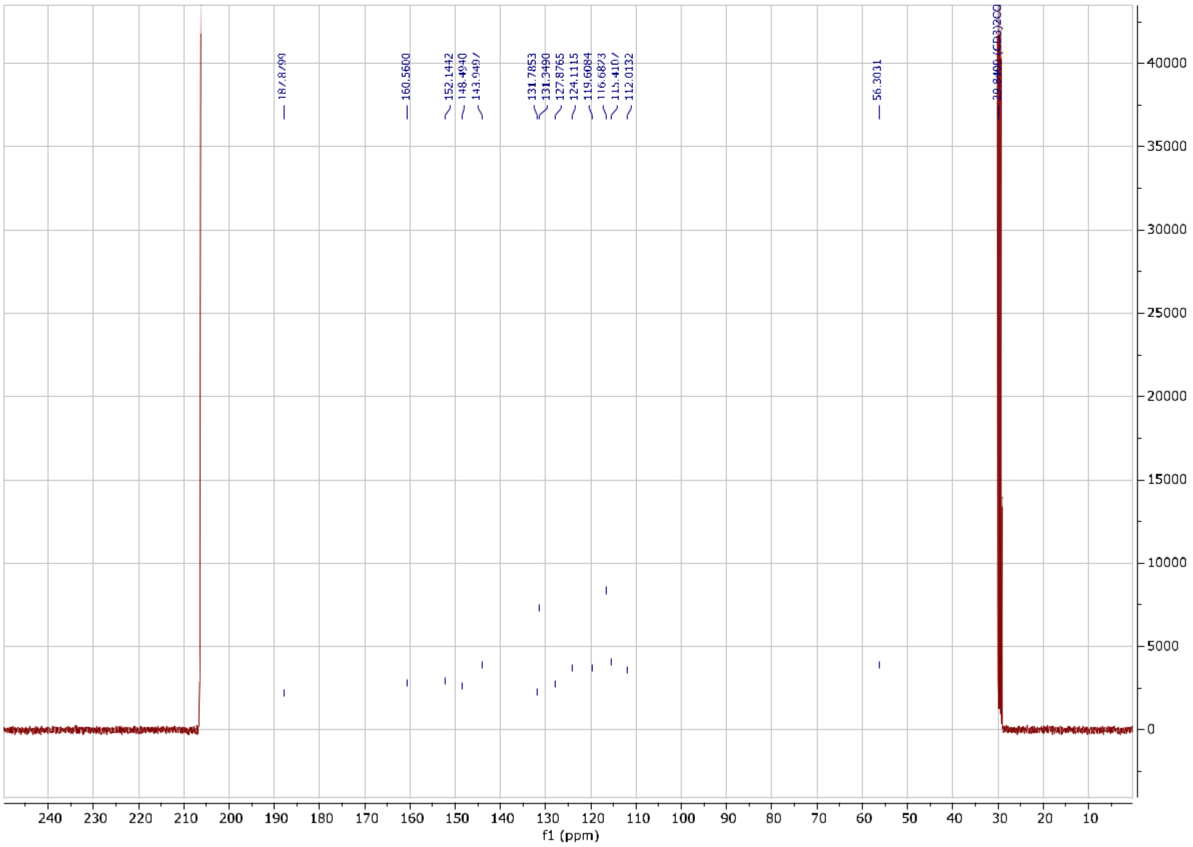

187.8799
160.5600
152.1442
148.4940
143.9497
131.7853
131.3490
127.8765
124.1115
119.6084
116.6823
115.4107
112.0132
56.3031
f1 (ppm)

a7

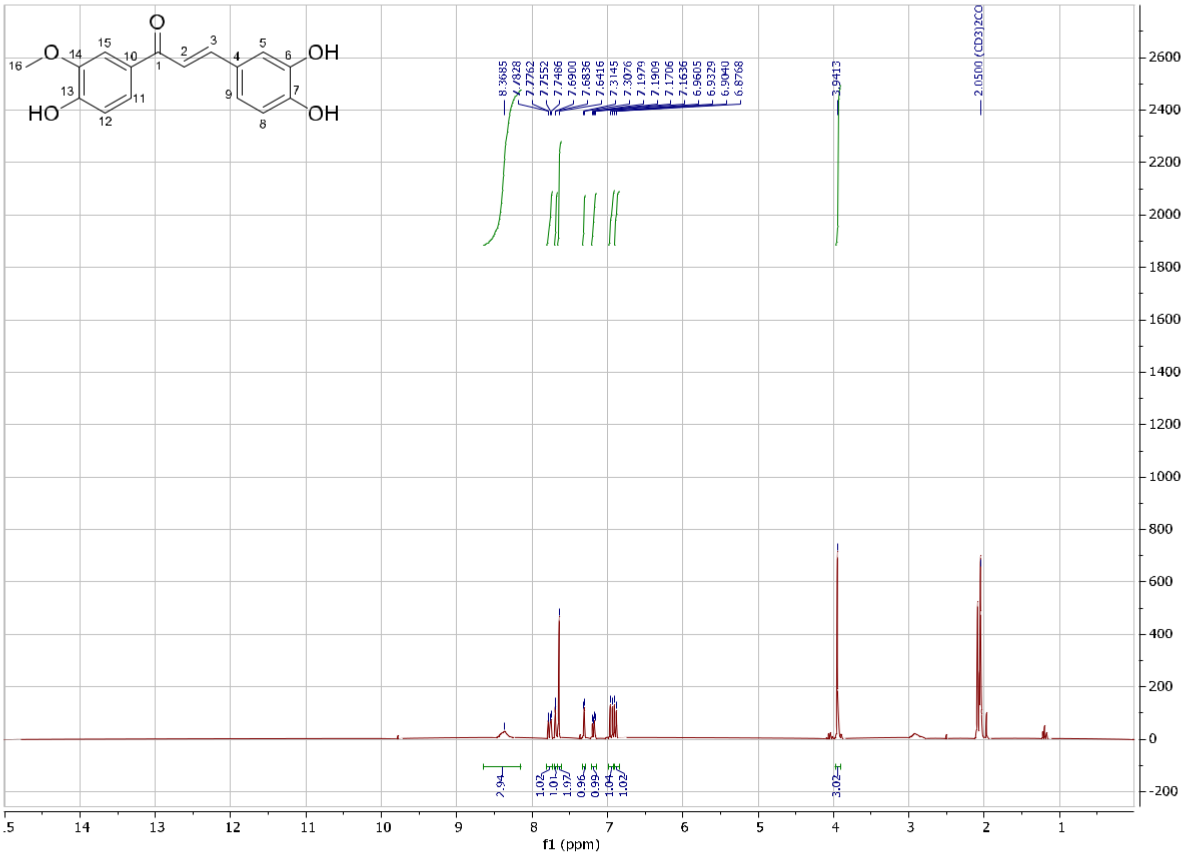

8.3685
7.7828
7.7762
7.7552
7.7486
7.6900
7.6836
7.6416
7.3145
7.3076
7.1979
7.1909
7.1706
7.1636
6.9605
6.9329
6.9040
6.8768
3.9413
2.0500 (CD3)2CO
2.94
1.02
1.01
1.97
0.96
0.99
1.04
1.02
3.02
f1 (ppm)

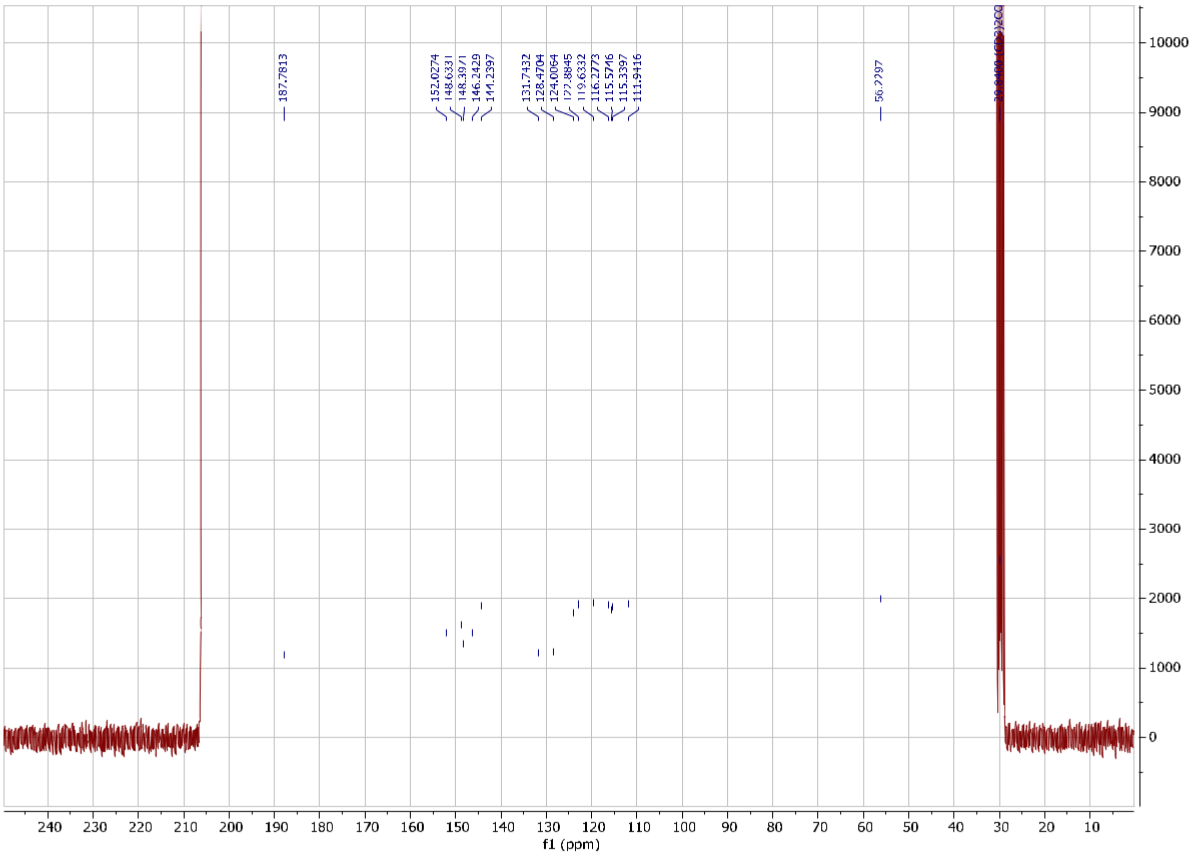

187.7813
152.0274
148.6331
146.2429
131.7432
128.4704
124.0064
119.6332
116.2773
115.5746
115.3397
111.9416
56.2297
f1 (ppm)

a8

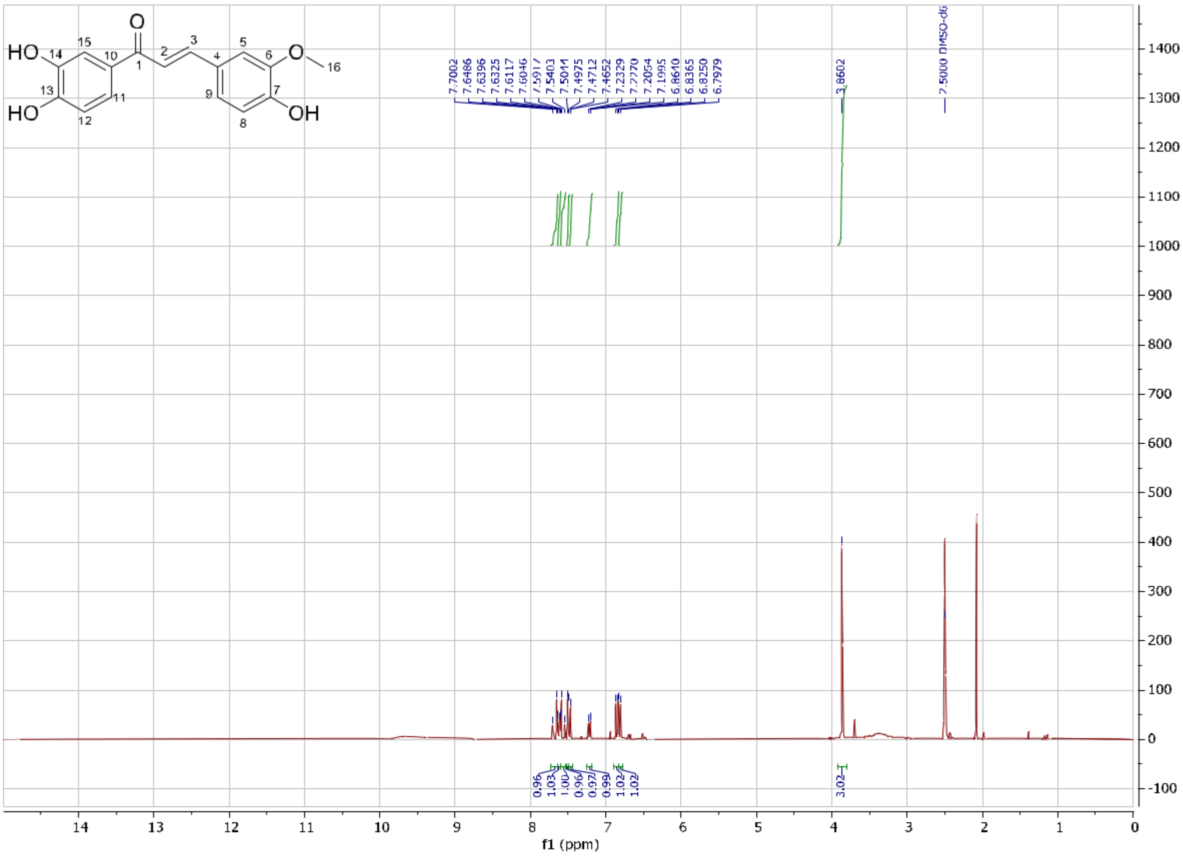

HO
HO
O
O
OH
7.7002 7.6486 7.6396 7.6325 7.6117 7.6046 7.5917 7.5403 7.5011 7.4975 7.4712 7.4652 7.2329 7.2270 7.2054 7.1995 6.8610 6.8365 6.8250 6.7979
3.8602
2.5000 DMSO-d6
0.96 1.03 1.00 0.96 0.97 0.99 1.02 1.02
3.02
f1 (ppm)

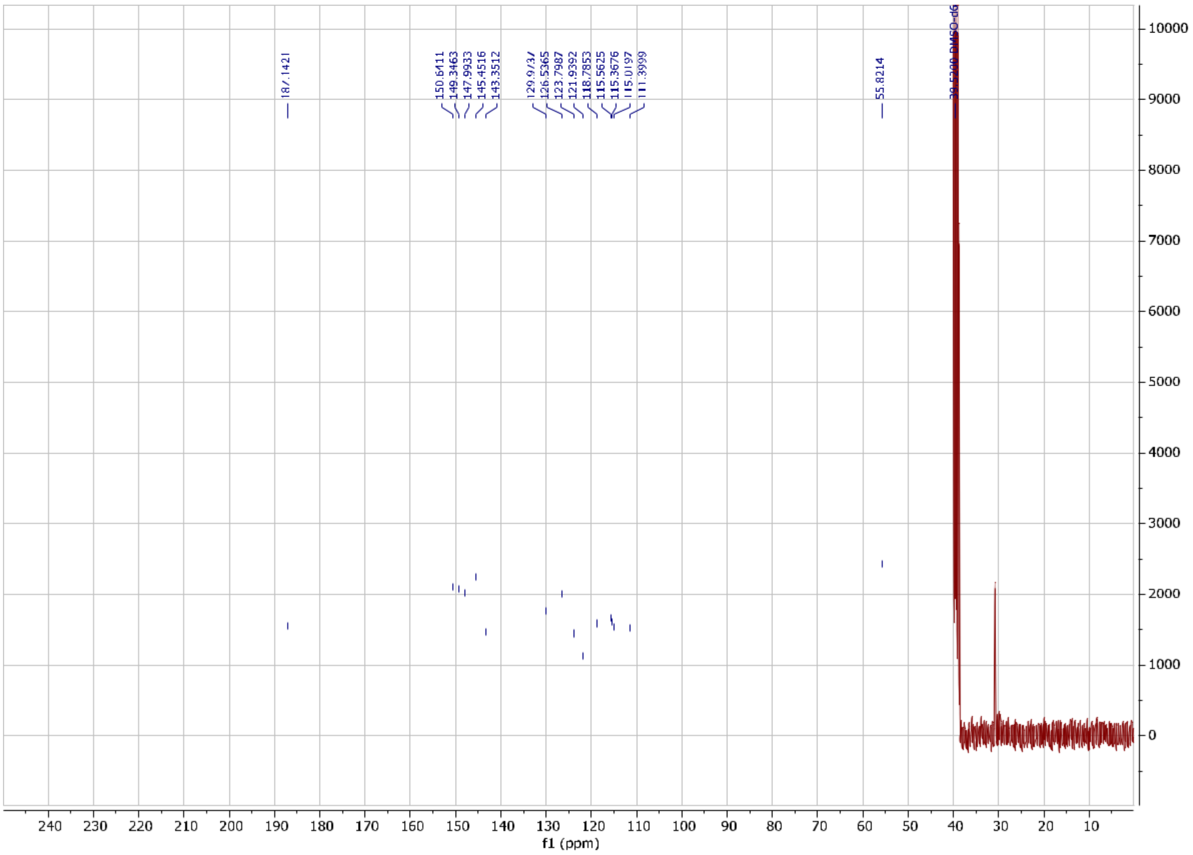

187.1421
150.6411 149.3463 147.9933 145.4516 143.3512
129.9737 126.5365 123.7987 121.9392 118.7853 115.5625 115.3676 115.0197 111.3999
55.8214
DMSO-d6
f1 (ppm)

a9

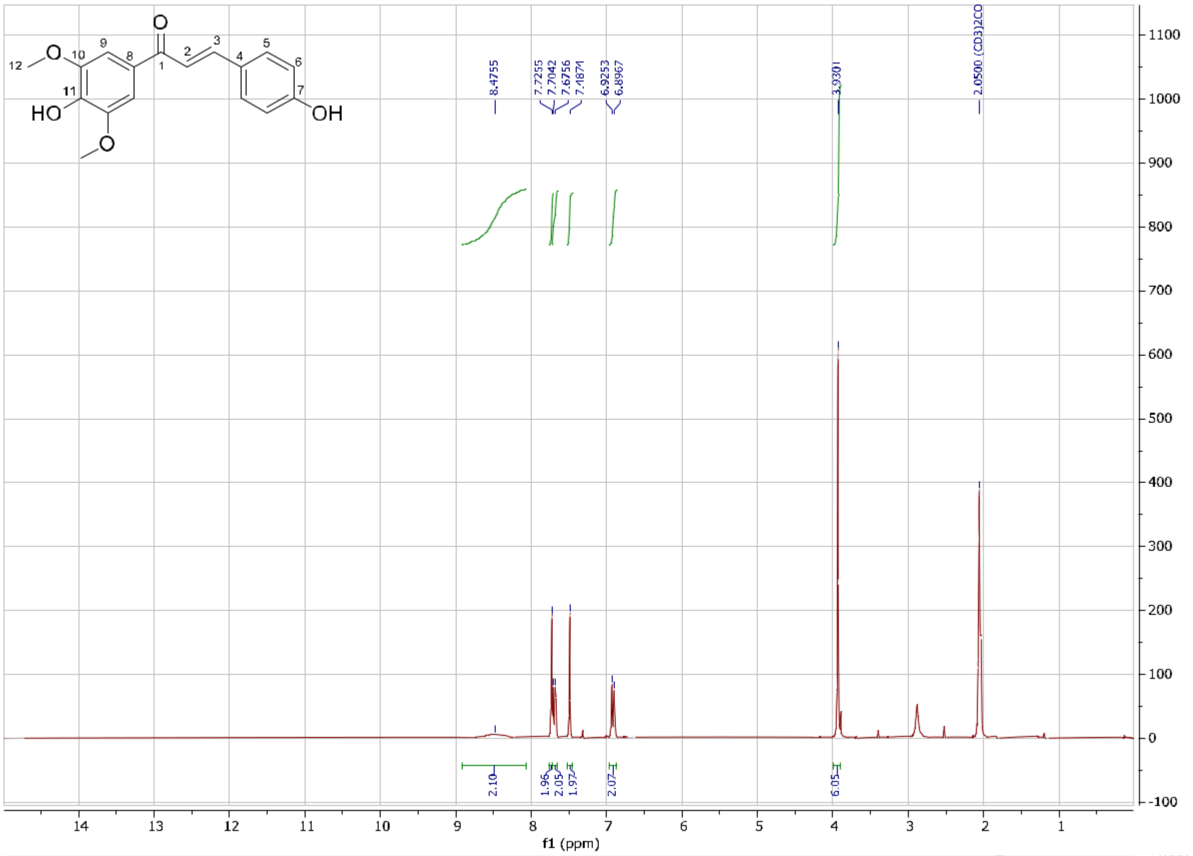
8.4755
7.7255
7.7042
7.6756
7.4874
6.9253
6.8967
3.9301
2.0500 (CD3)2CO
2.10
1.96
2.05
1.97
2.07
6.05
f1 (ppm)

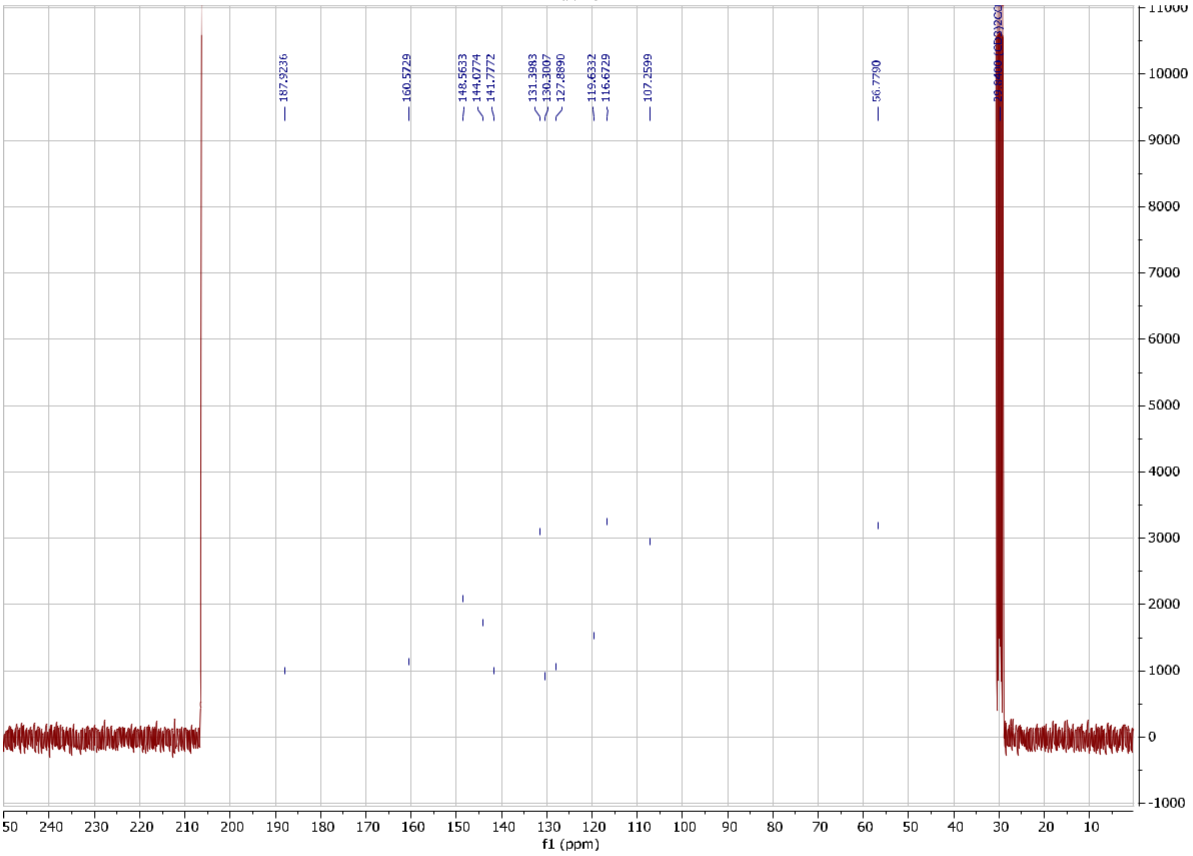
187.9236
160.5729
148.5633
144.0774
141.7772
131.3983
130.3007
127.8890
119.6332
116.6729
107.2599
56.7790
f1 (ppm)

a10

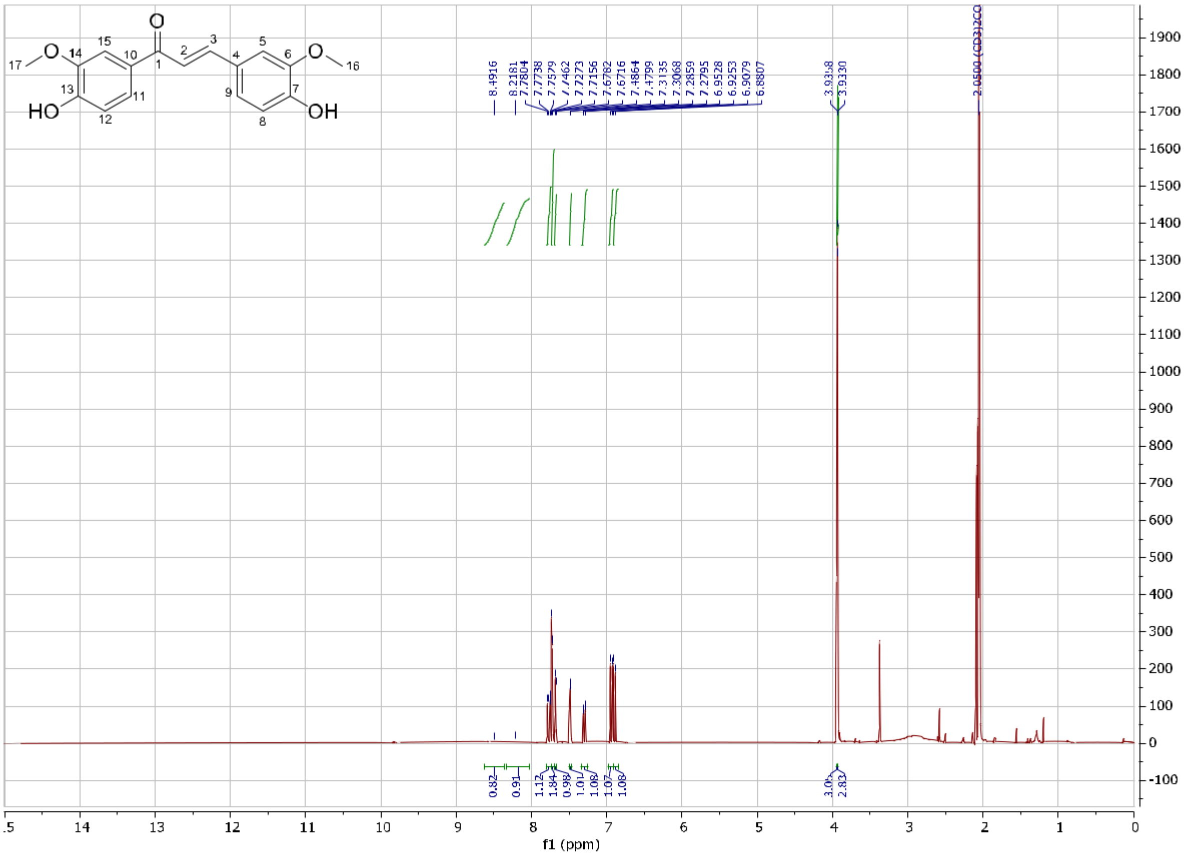

8.4916
8.2181
7.7804
7.7738
7.7529
7.7462
7.7273
7.7156
7.6782
7.6716
7.4864
7.4799
7.3135
7.3068
7.2859
7.2795
6.9528
6.9253
6.9079
6.8807
3.9358
3.9330
2.0500 (CD3)2CO
f1 (ppm)

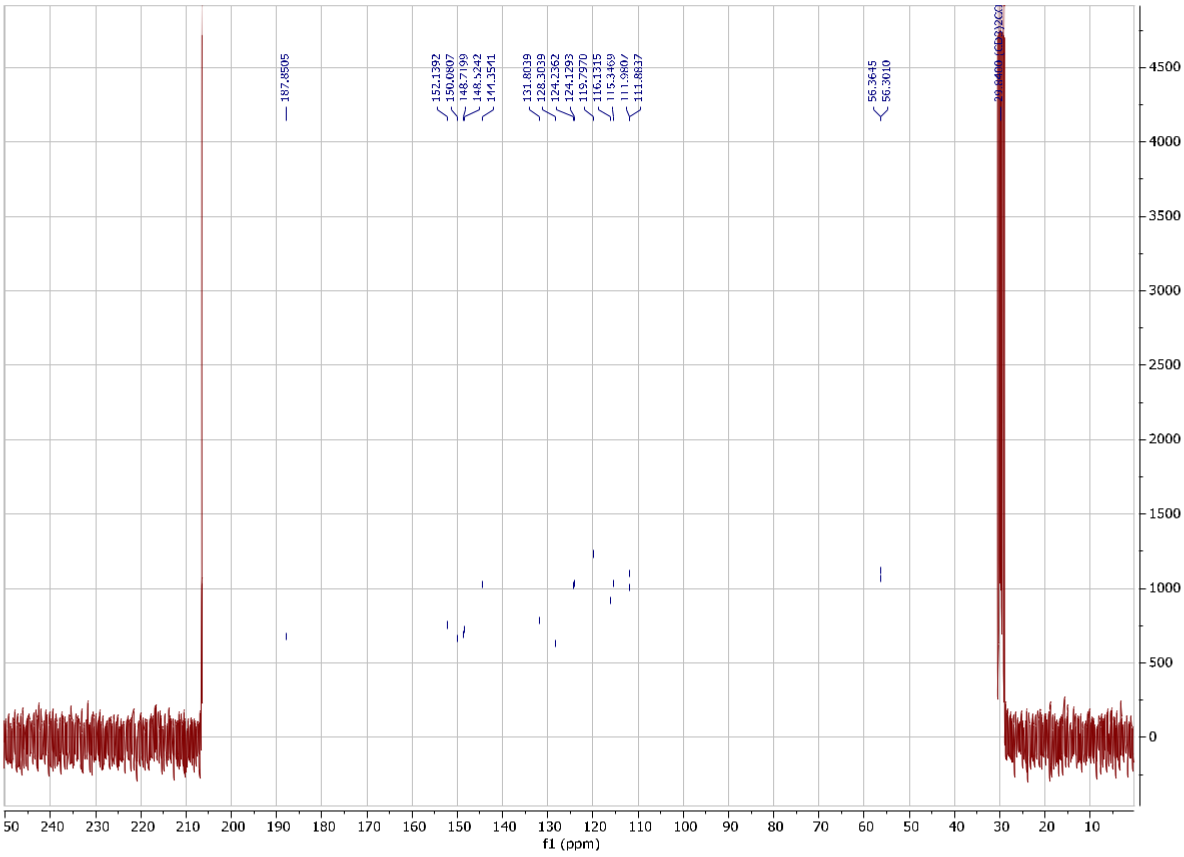

187.8505
152.1392
150.0807
148.7199
148.5242
144.3541
131.8039
128.3039
124.2362
124.1293
119.7970
116.1315
115.3469
111.9807
111.8837
56.3645
56.3010
f1 (ppm)

a11

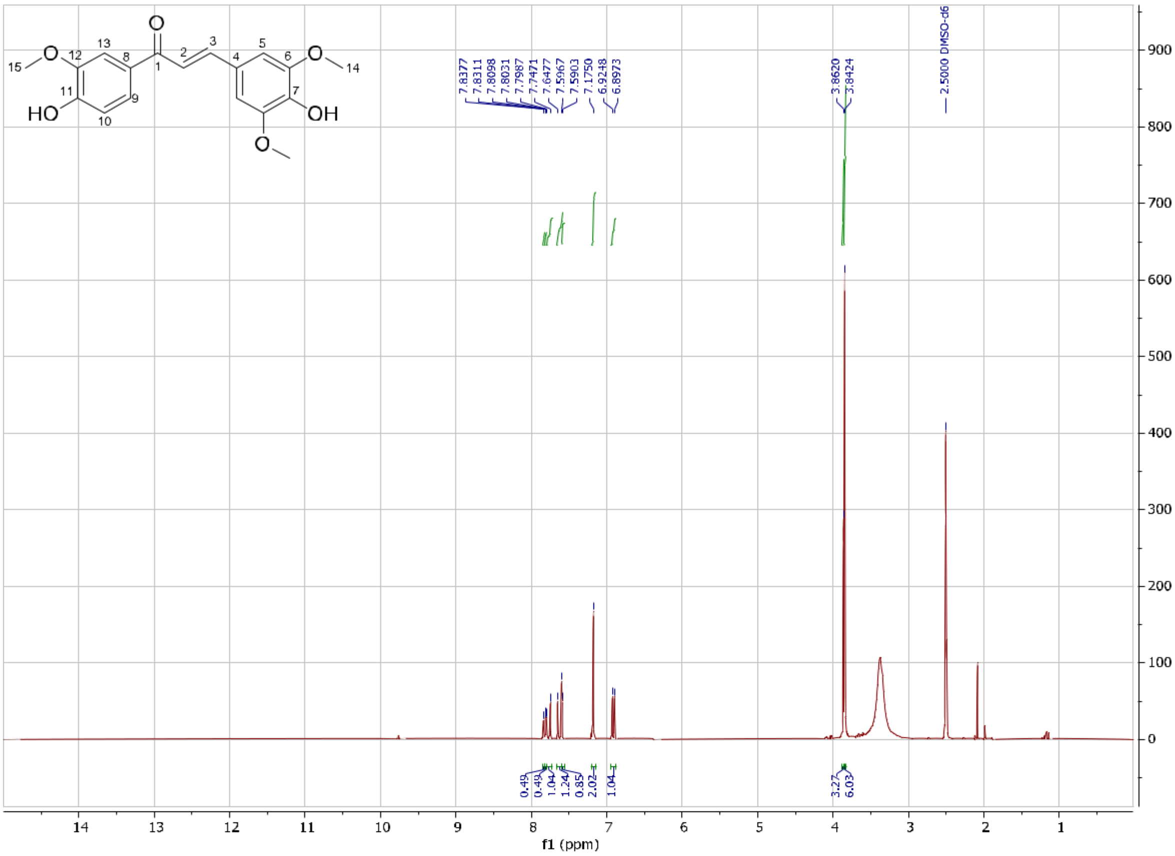
15
12
13
8
1
2
3
4
5
6
14
11
9
10
7
HO
OH
7.8377
7.8311
7.8098
7.8031
7.7987
7.7471
7.6477
7.5967
7.5903
7.1750
6.9248
6.8973
3.8620
3.8424
2.5000 DMSO-d6
0.49
0.49
1.04
1.24
0.85
2.02
1.04
3.27
6.03
f1 (ppm)

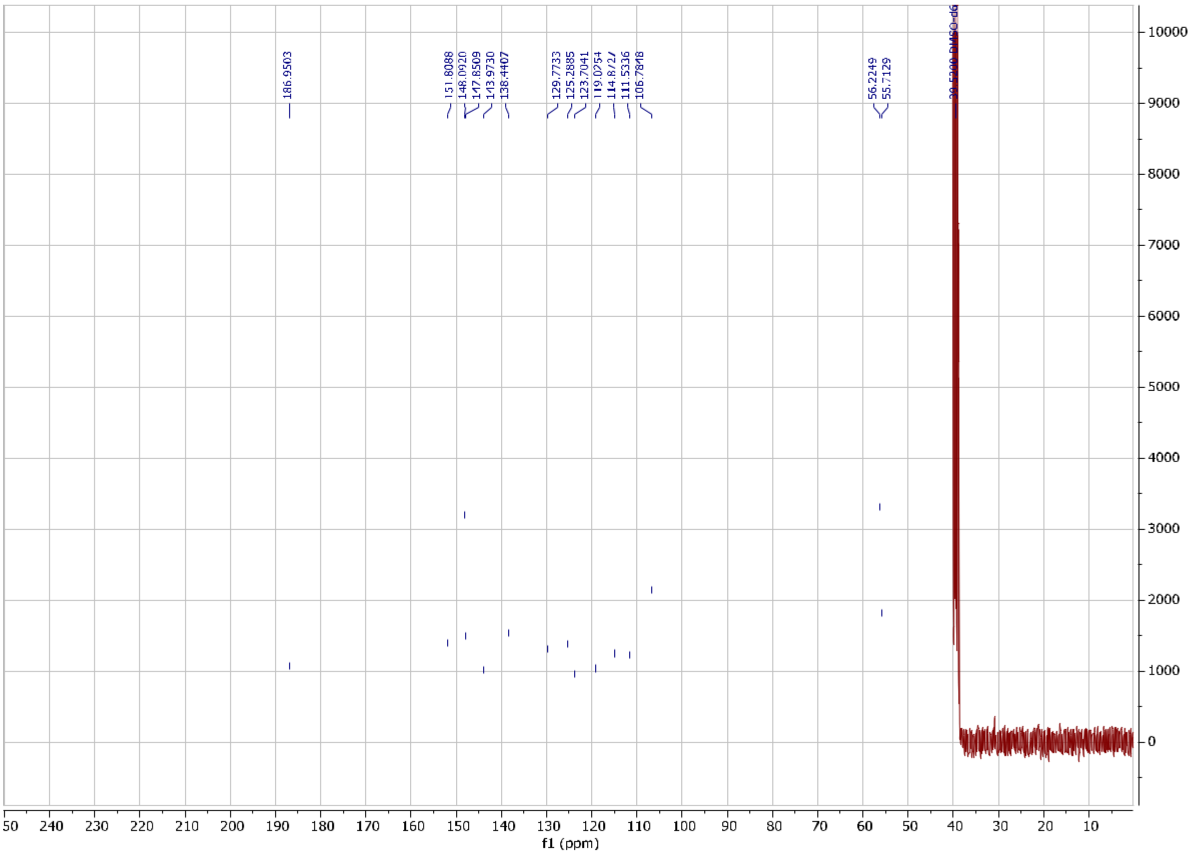
186.9503
151.8088
148.0920
147.8509
143.9730
138.4407
129.7733
125.2885
123.7041
119.0754
114.8727
111.5336
106.7848
56.2249
55.7129
DMSO-d6
f1 (ppm)

a12

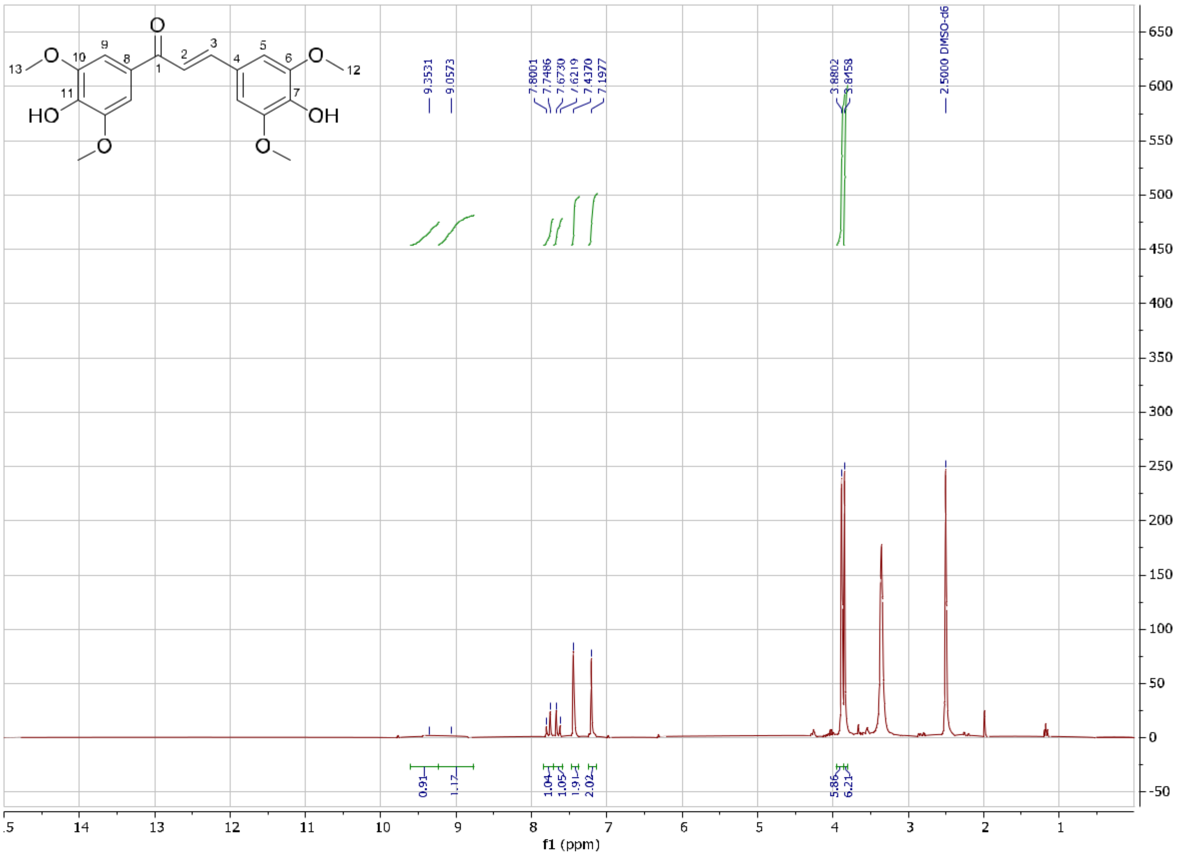
HO
OH
9.3531
9.0573
7.8001
7.7486
7.6730
7.6219
7.4370
7.1977
3.8802
3.8458
2.5000 DMSO-d6
0.91
1.17
1.04
1.05
1.91
2.02
5.86
6.21
f1 (ppm)

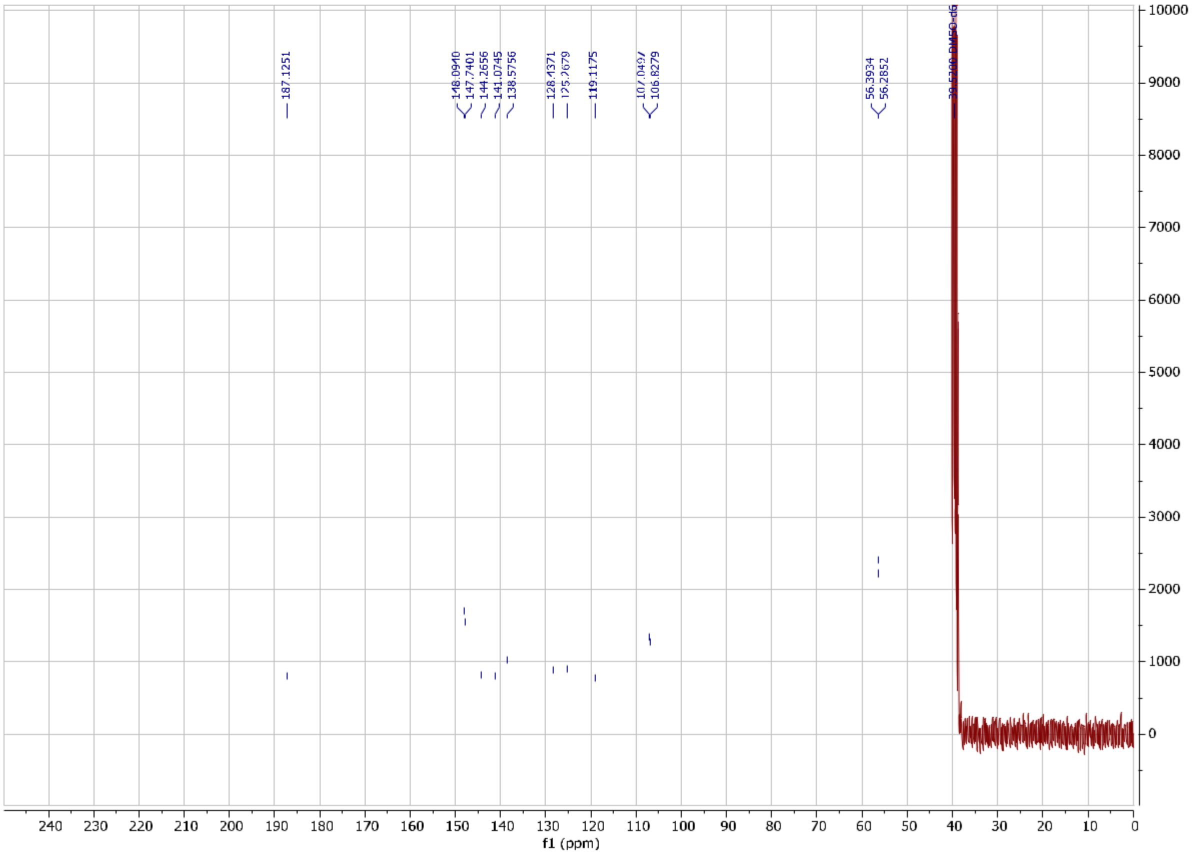
187.1251
148.0940
147.7401
144.2656
141.0745
138.5756
128.4371
125.2679
119.1175
107.0497
106.8279
56.3934
56.2852
f1 (ppm)

2. 1H & 13C NMR spectra saturated molecules (series b)

b1

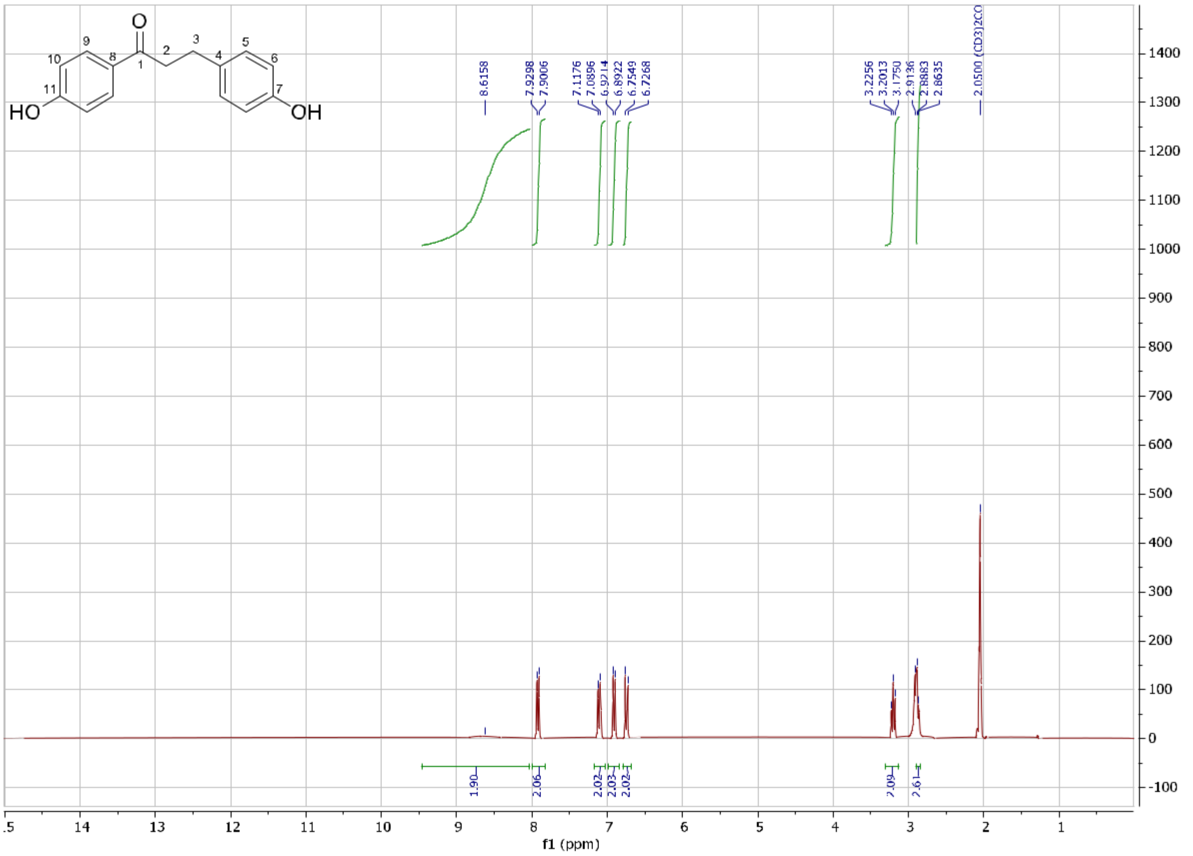

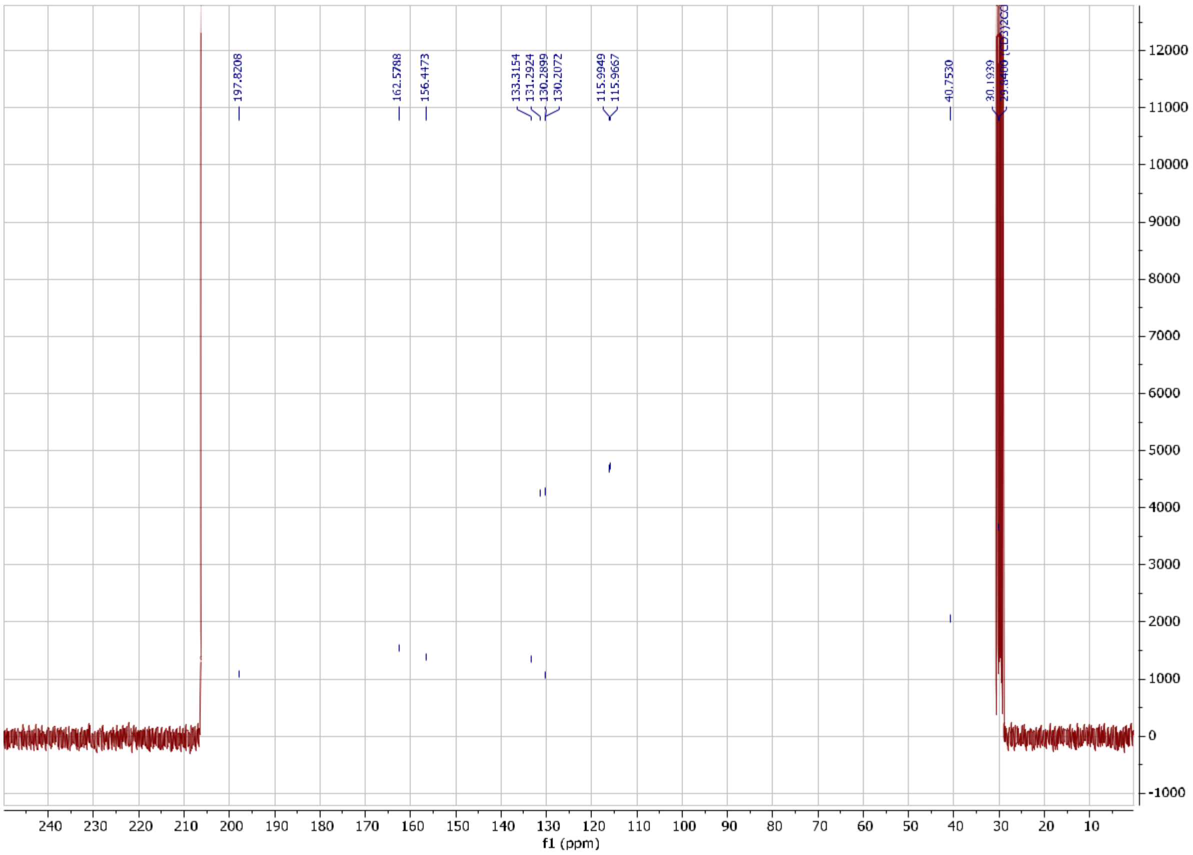

b2

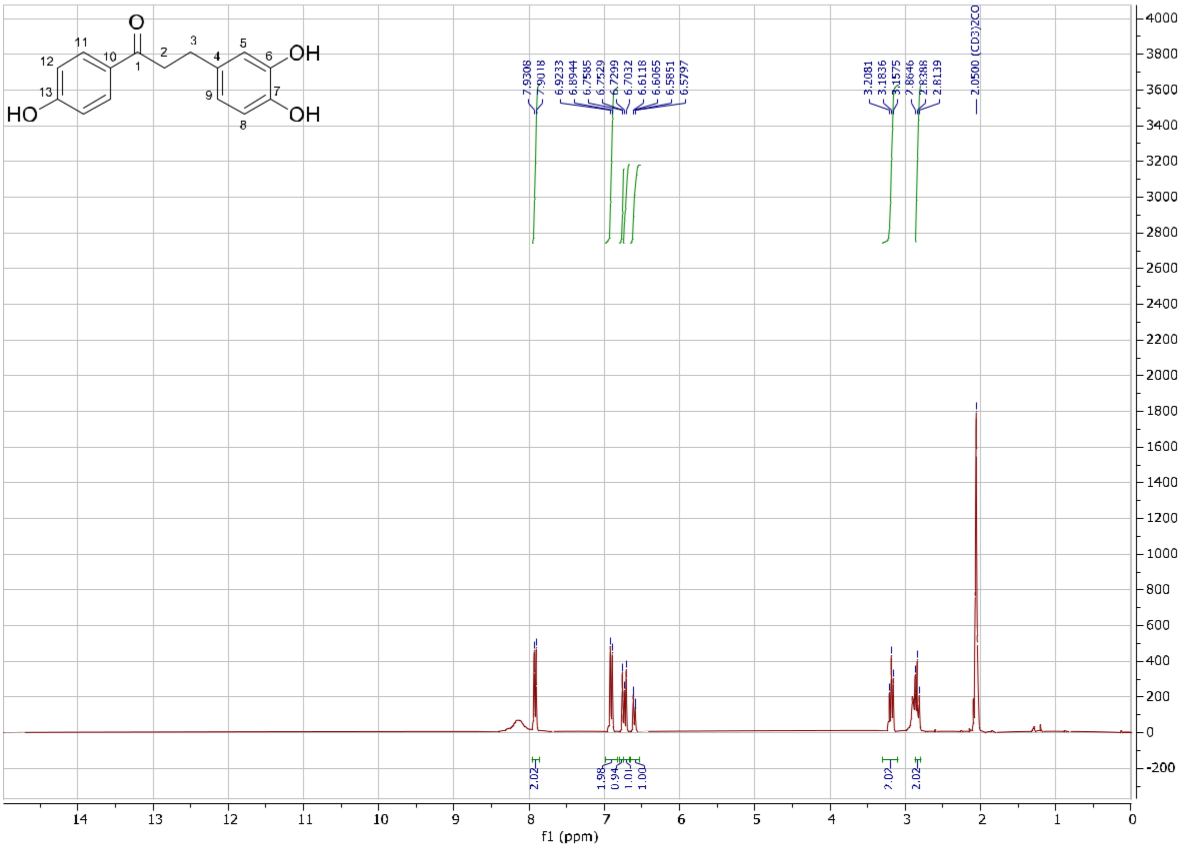
OH
OH
HO
O
7.9308
7.9018
2.0500 (CD3)2CO
f1 (ppm)

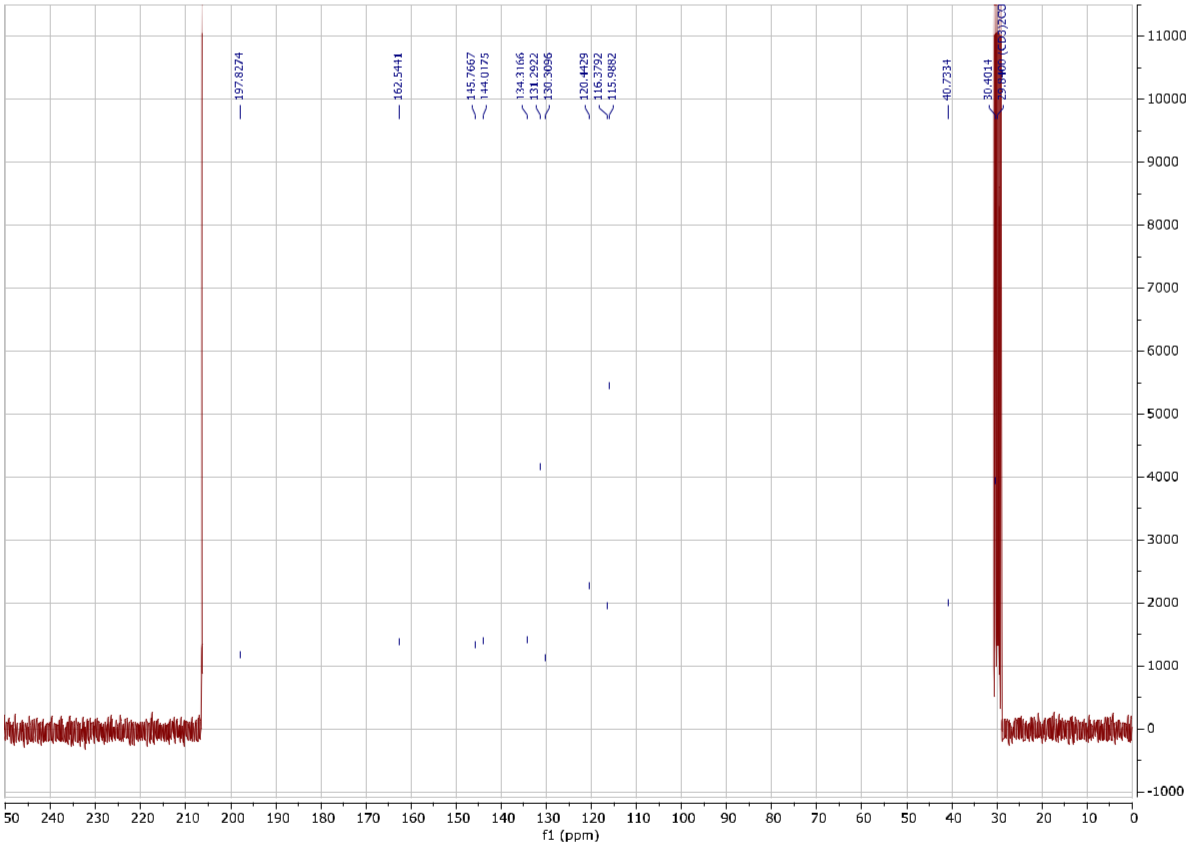
197.8274
162.5411
145.7667
144.0175
134.3166
131.2922
130.3096
120.4429
116.3792
115.9882
40.7334
30.4014
f1 (ppm)

b3

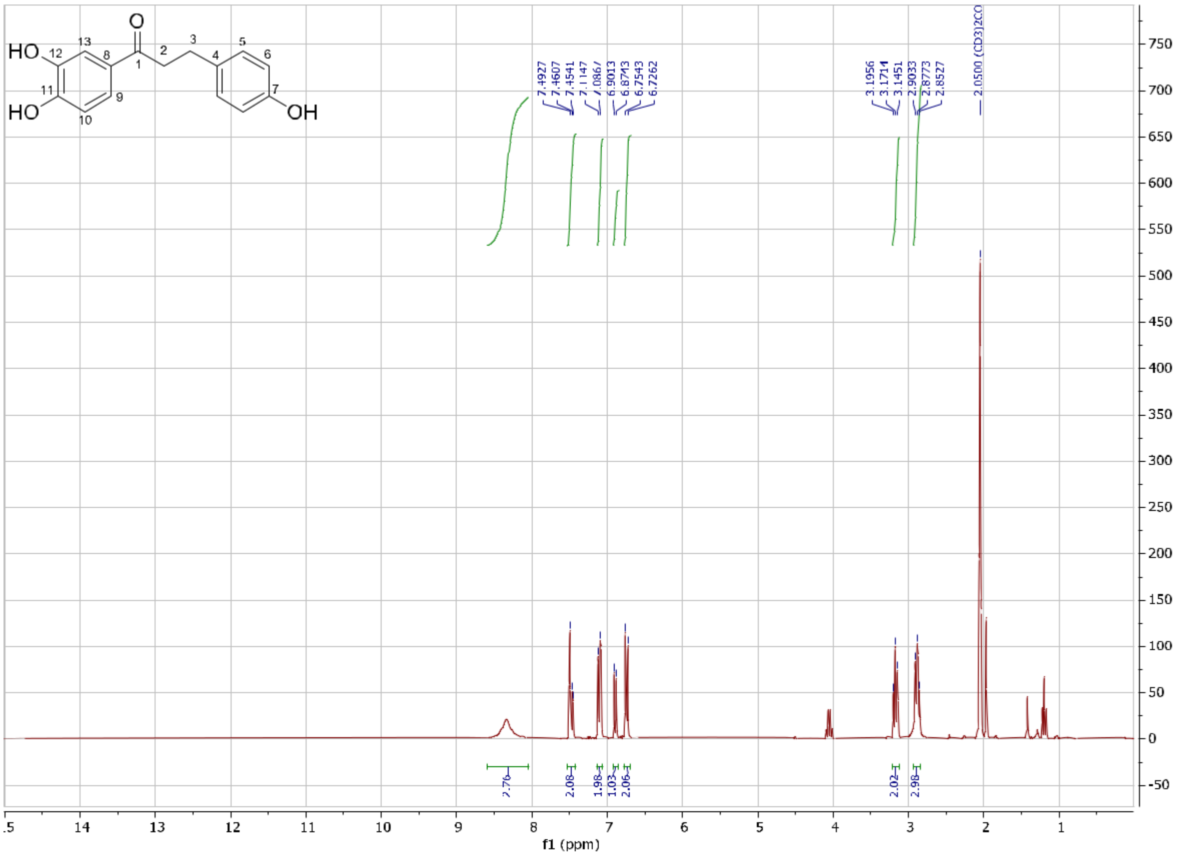
HO
HO
OH
O
1
2
3
4
5
6
7
8
9
10
11
12
13
7.4927
7.4607
7.4541
7.1147
7.0867
6.9013
6.8743
6.7543
6.7262
3.1956
3.1714
3.1451
2.9033
2.8773
2.8527
2.0500 (CD3)2CO
2.76
2.08
1.98
1.03
2.06
2.02
2.98
f1 (ppm)

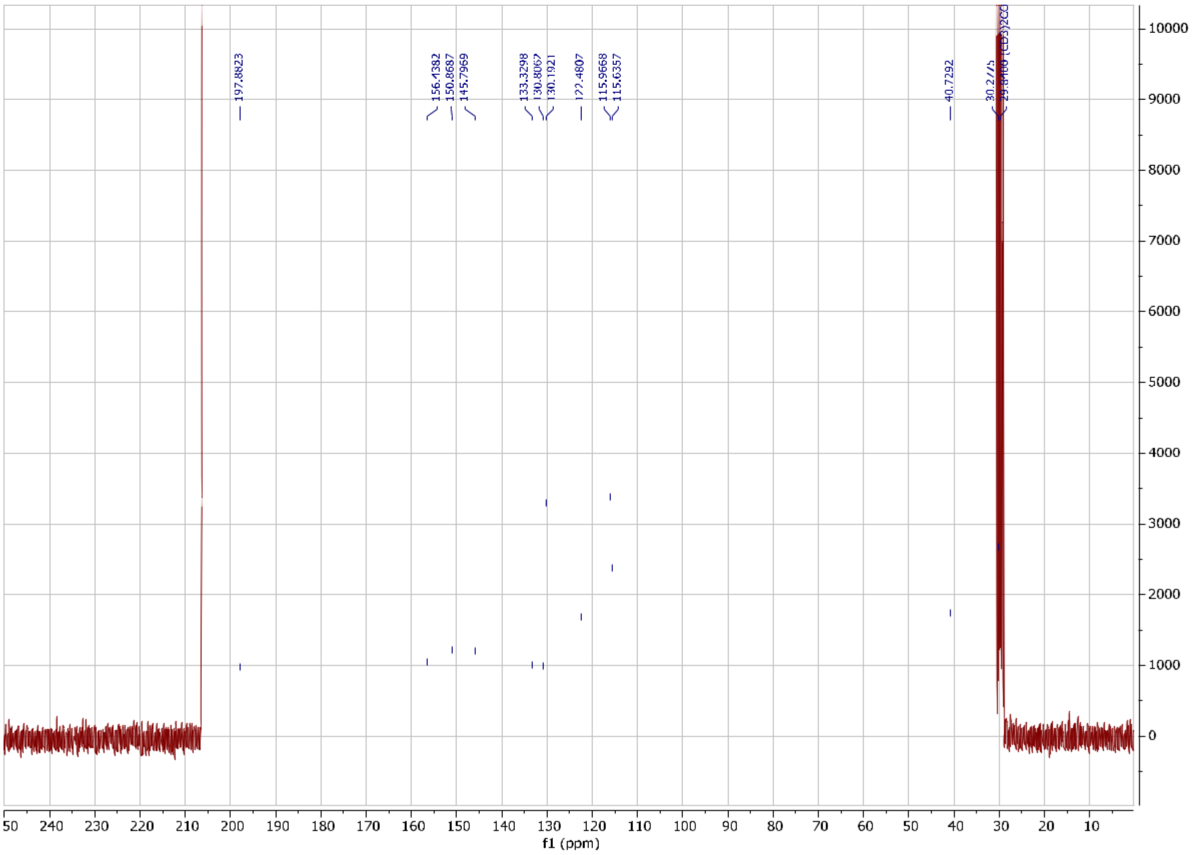
197.8823
156.4382
150.8687
145.7969
133.3298
130.8062
130.1921
122.4807
115.9668
115.6357
40.7292
30.2775
f1 (ppm)

b4

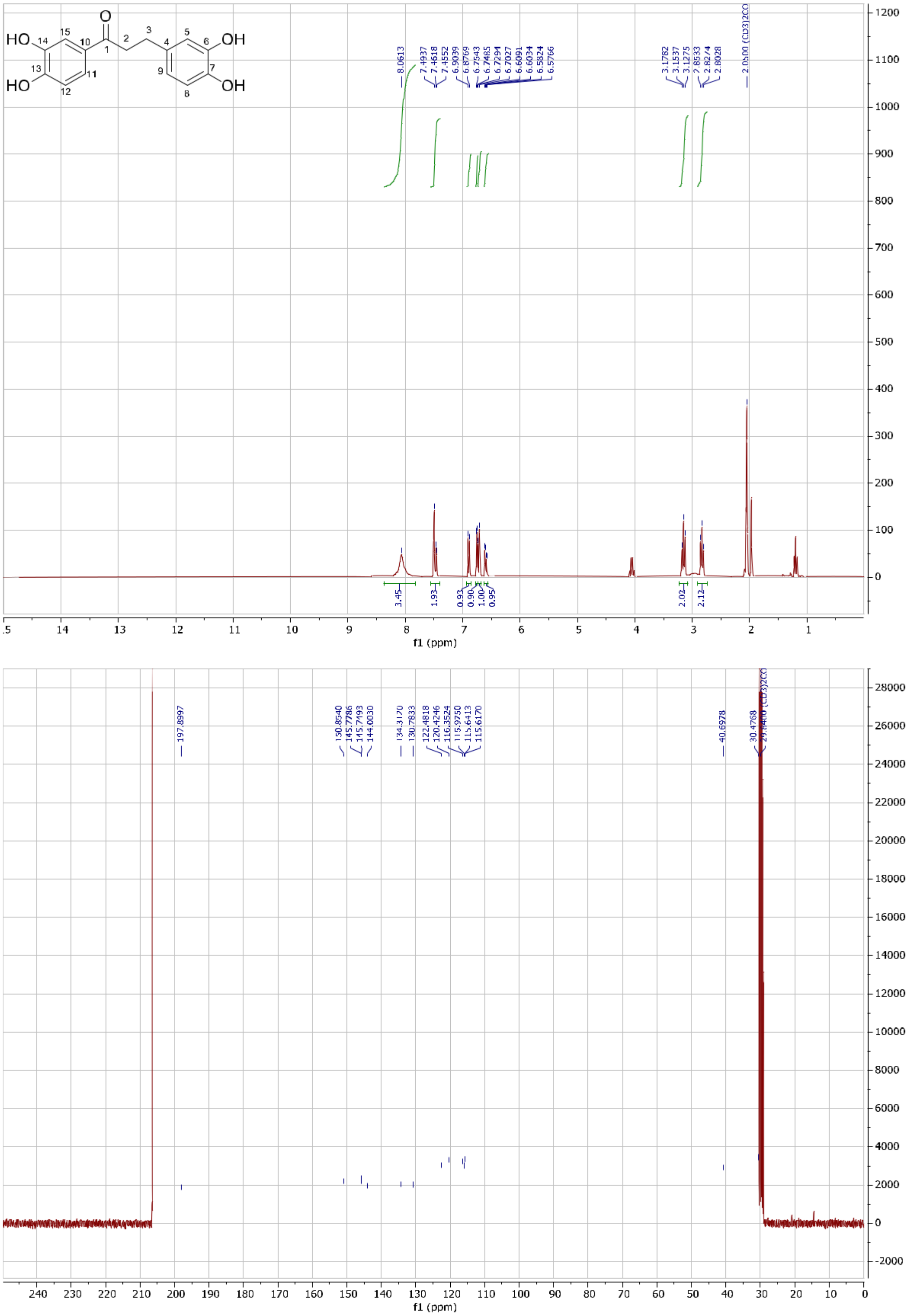
HO
HO
OH
OH
O
8.0613
7.4937
7.4618
7.4552
6.9039
6.8769
6.7543
6.7485
6.7294
6.7027
6.6091
6.6034
6.5824
6.5766
3.1782
3.1537
3.1275
2.8533
2.8274
2.8028
2.0500 (CD3)2CO
3.45
1.93
0.93
0.90
1.00
0.95
2.02
2.12
f1 (ppm)
197.8997
150.8540
145.7786
145.7193
144.0030
134.3120
130.7833
122.4818
120.4246
116.3524
115.9750
115.6413
115.6170
40.6978
30.4768
29.8400 (CD3)2CO
f1 (ppm)

b5

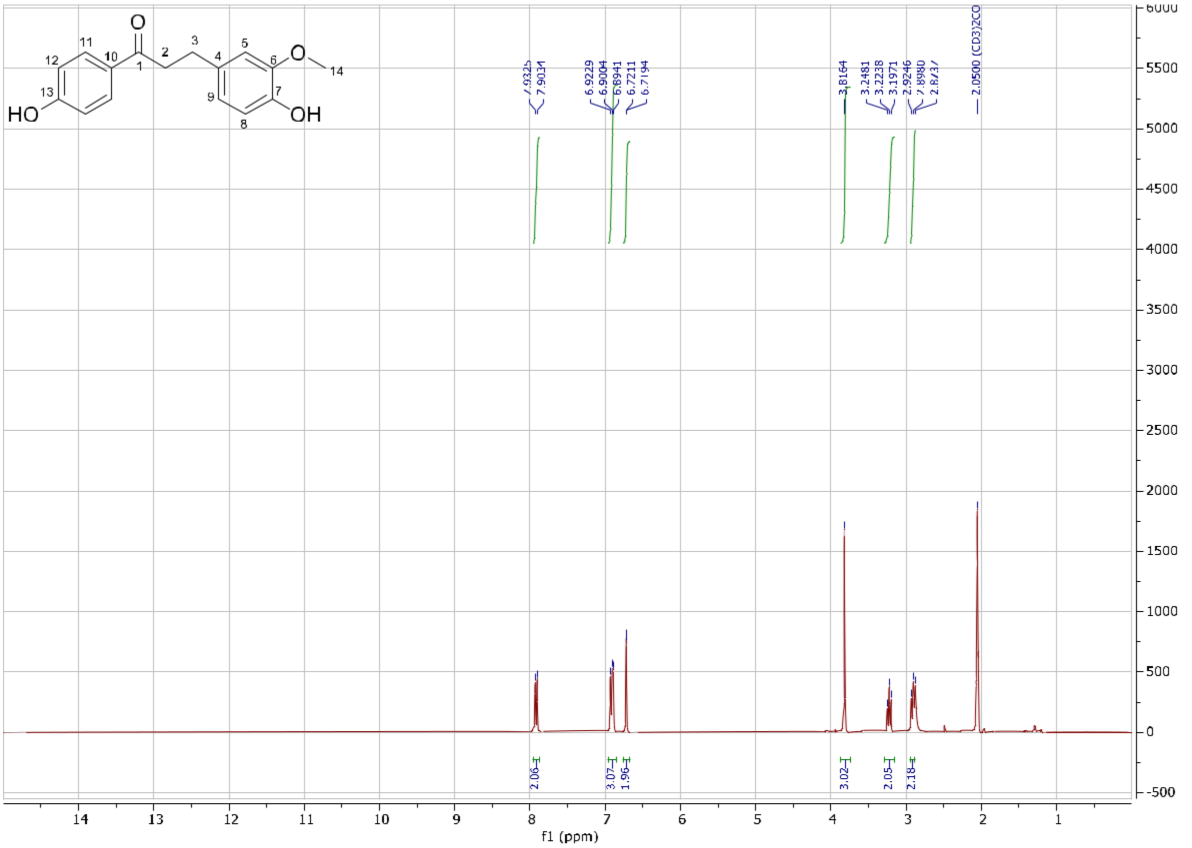
HO
OH
O
14
f1 (ppm)
3.8164
2.0500 (CD3)2CO
2.06
3.07
1.96
3.02
2.05
2.18

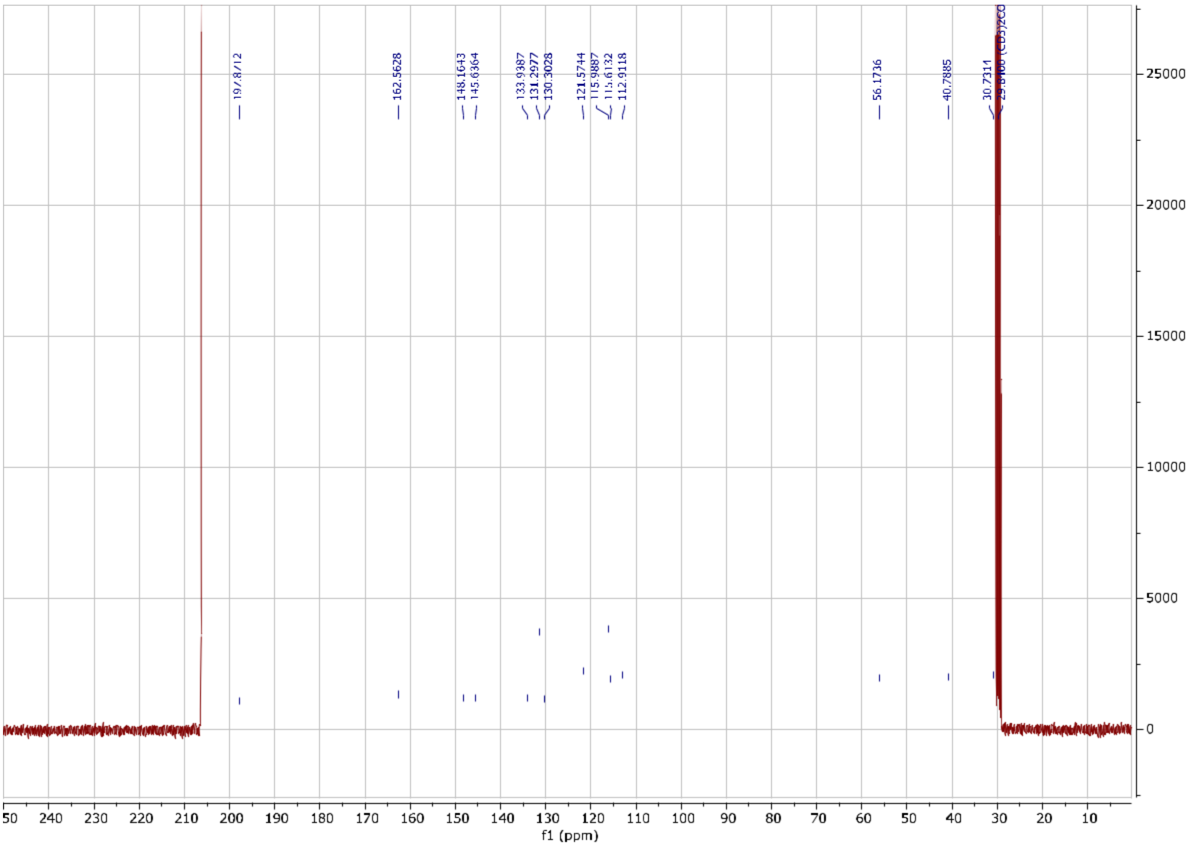
162.5628
148.1643
145.6364
133.9387
131.2977
130.3028
121.5744
56.1736
40.7885
30.7311
f1 (ppm)

b6

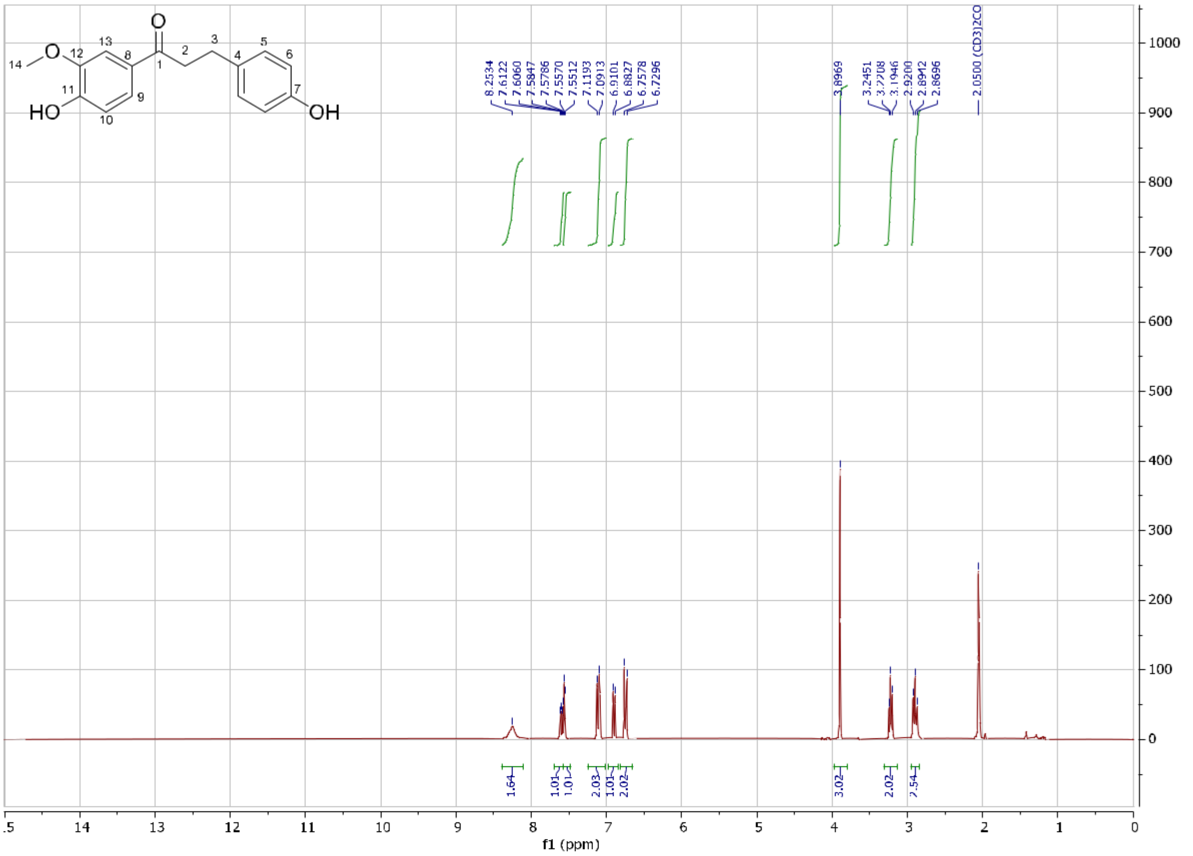

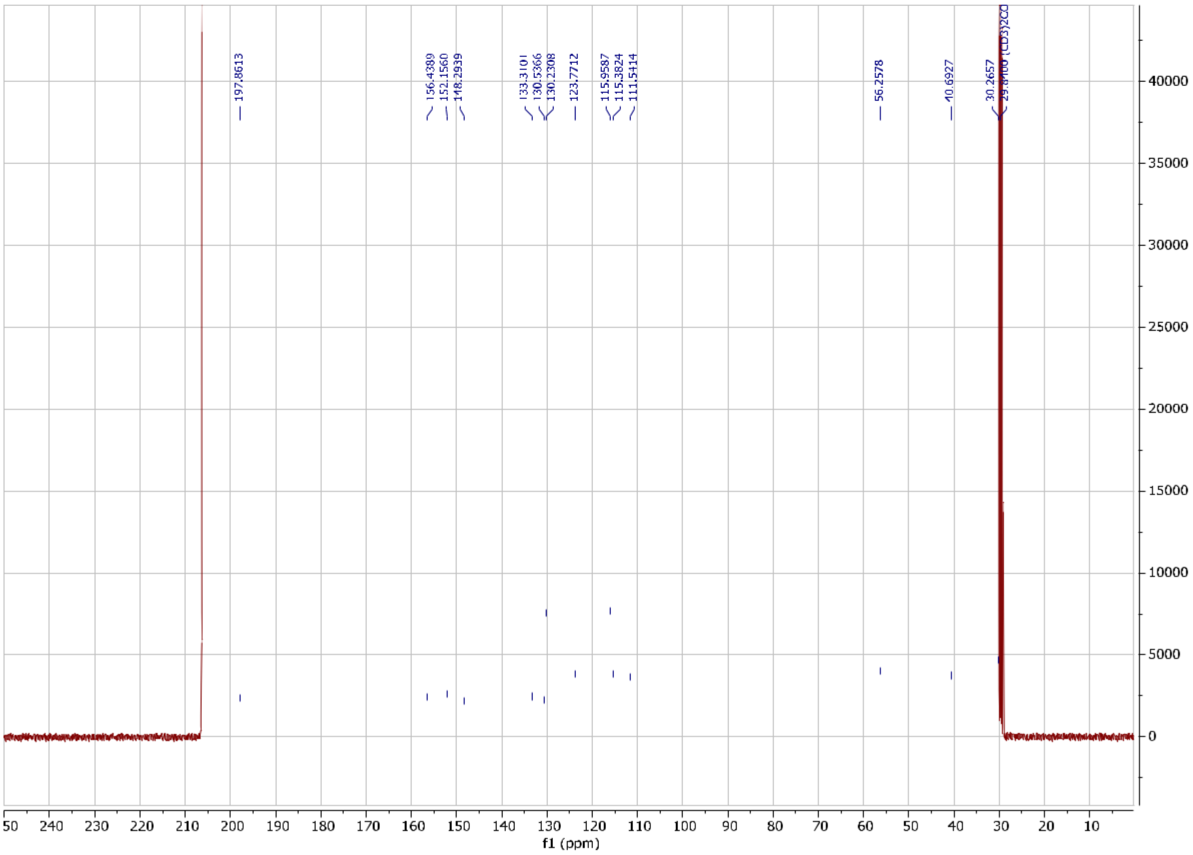

b7

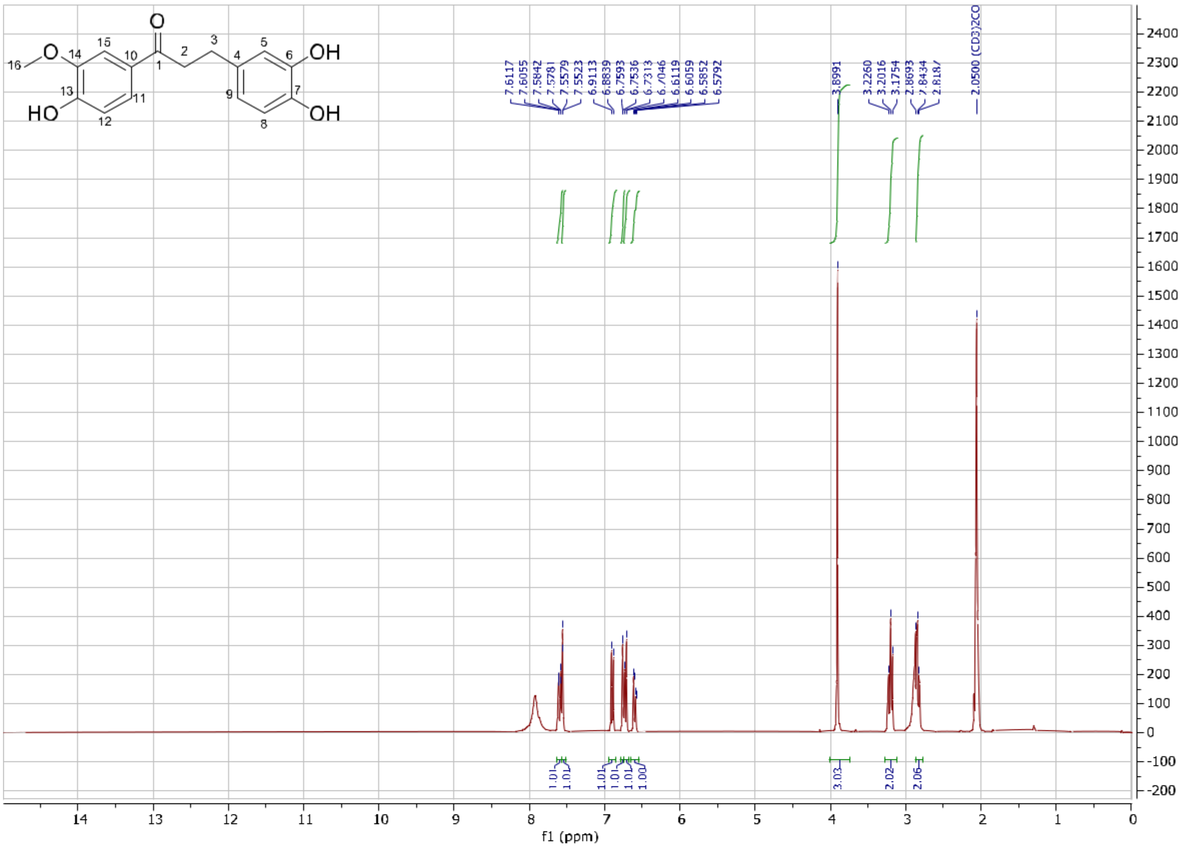
7.6117
7.6055
7.5842
7.5781
7.5579
7.5523
6.9113
6.8839
6.7593
6.7536
6.7313
6.6119
6.6059
6.5852
6.5792
3.8991
3.2260
3.2016
3.1754
2.8693
2.8434
2.0500 (CD3)2CO
1.01
1.01
1.01
1.01
1.01
1.00
3.03
2.02
2.06
f1 (ppm)
OH
OH
HO
O

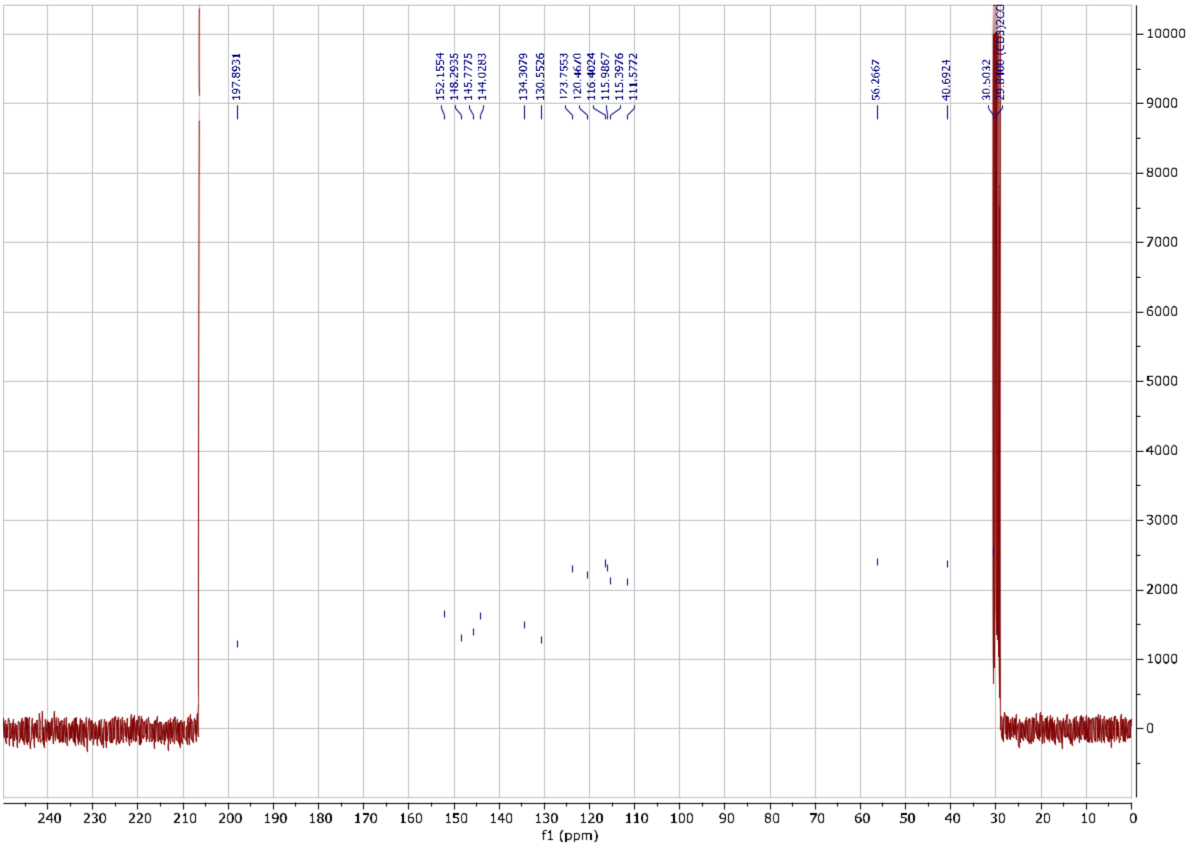
197.8931
152.1554
148.2935
145.7775
144.0283
134.3079
130.5526
123.7553
115.9867
115.3976
111.5772
56.2667
40.6924
30.5032
(CD3)2CO
f1 (ppm)

b8

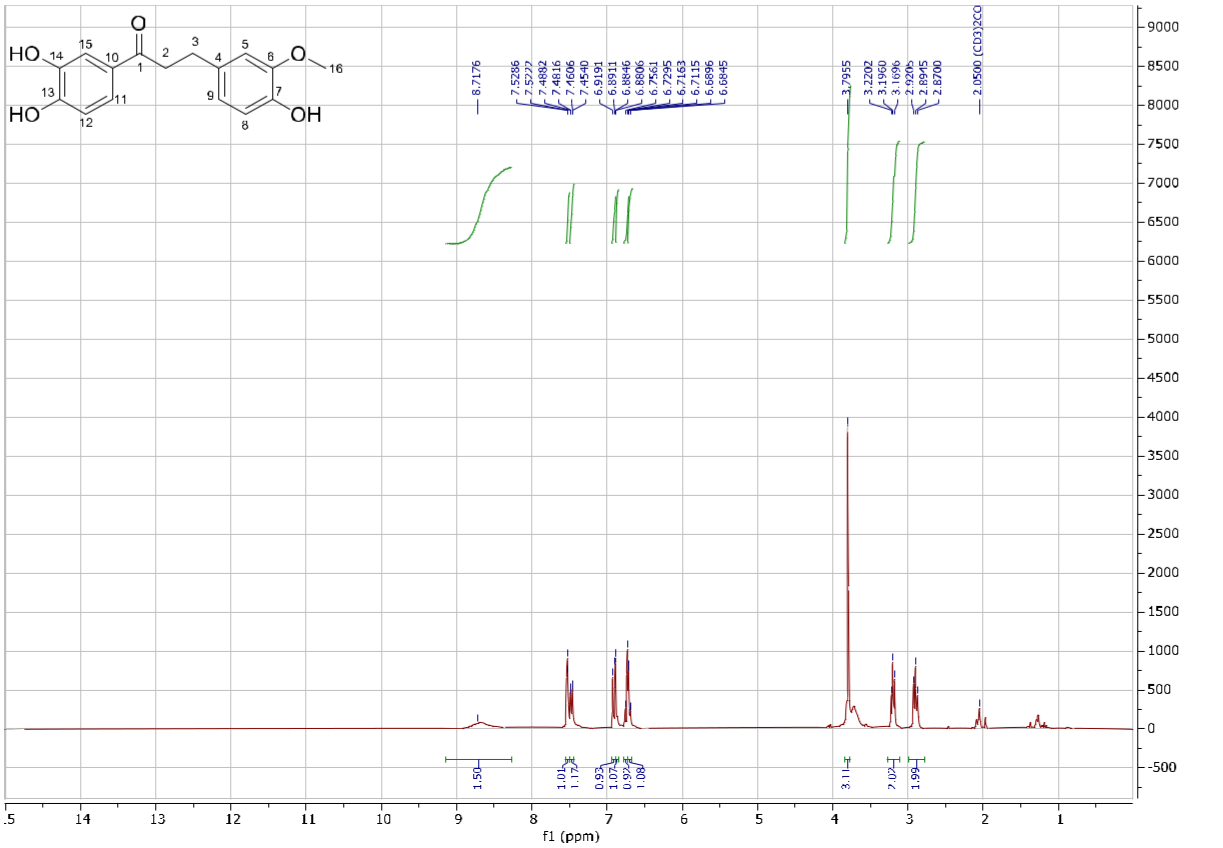
HO
HO
OH
O
8.7176
7.5286
7.5222
7.4882
7.4816
7.4606
7.4540
6.9191
6.8911
6.8846
6.8806
6.7561
6.7295
6.7163
6.7115
6.6896
6.6845
3.7955
3.2202
3.1960
3.1696
2.9205
2.8945
2.8700
2.0500 (CD3)2CO
1.50
1.01
1.17
0.93
1.07
0.92
1.08
3.11
2.02
1.99
f1 (ppm)

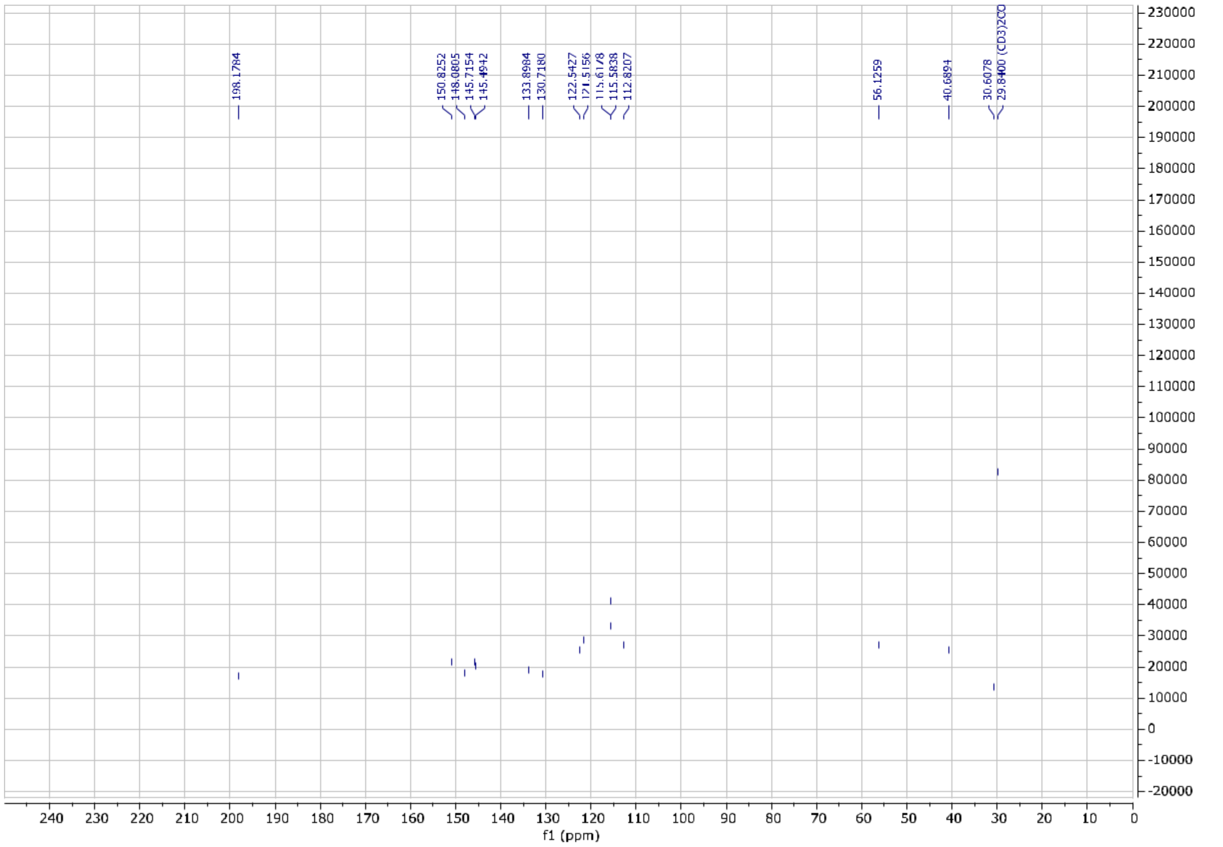
198.1784
150.8252
148.0805
145.7154
145.4942
133.8984
130.7180
122.5427
121.5156
115.6178
115.5838
112.8207
56.1259
40.6894
30.6078
29.8400 (CD3)2CO
f1 (ppm)

b9

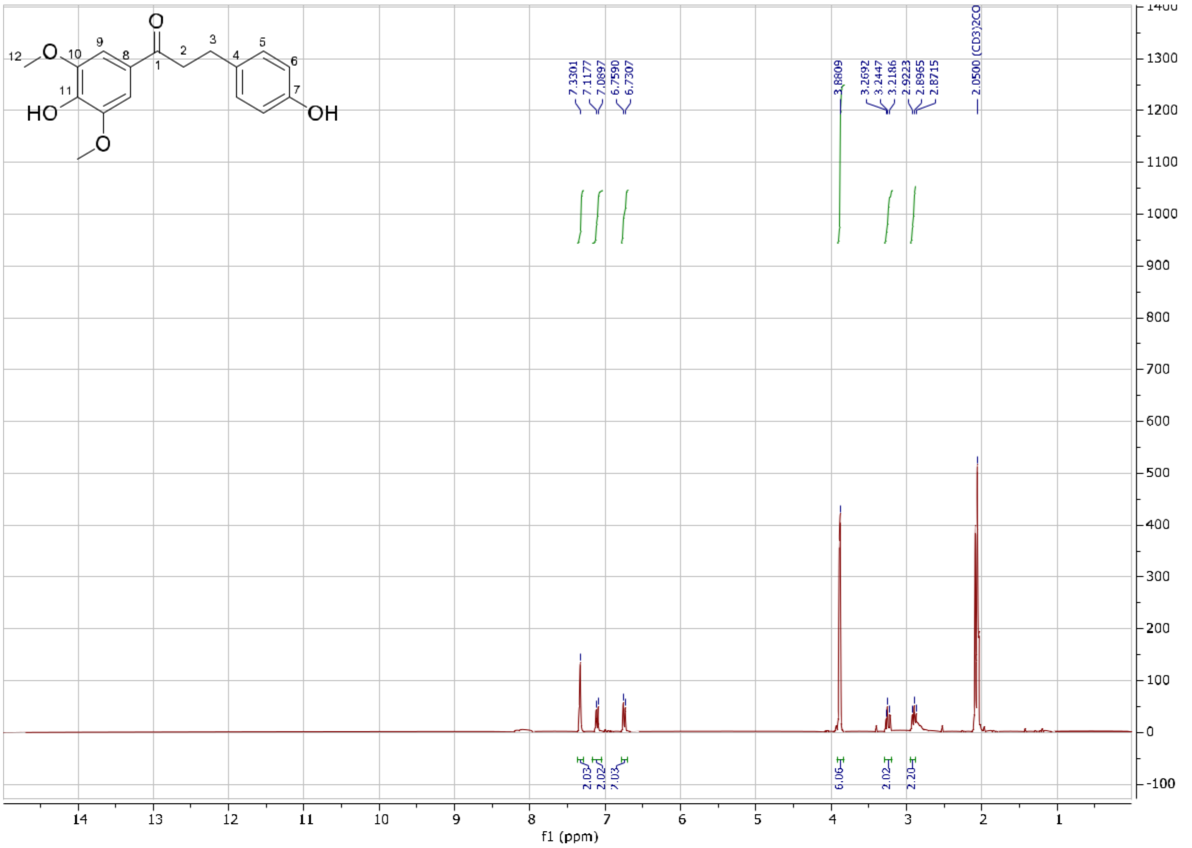
HO
OH
7.3301
7.1177
7.0897
6.7590
6.7307
3.8809
3.2692
3.2447
3.2186
2.9223
2.8965
2.8715
2.0500 (CD3)2CO
f1 (ppm)

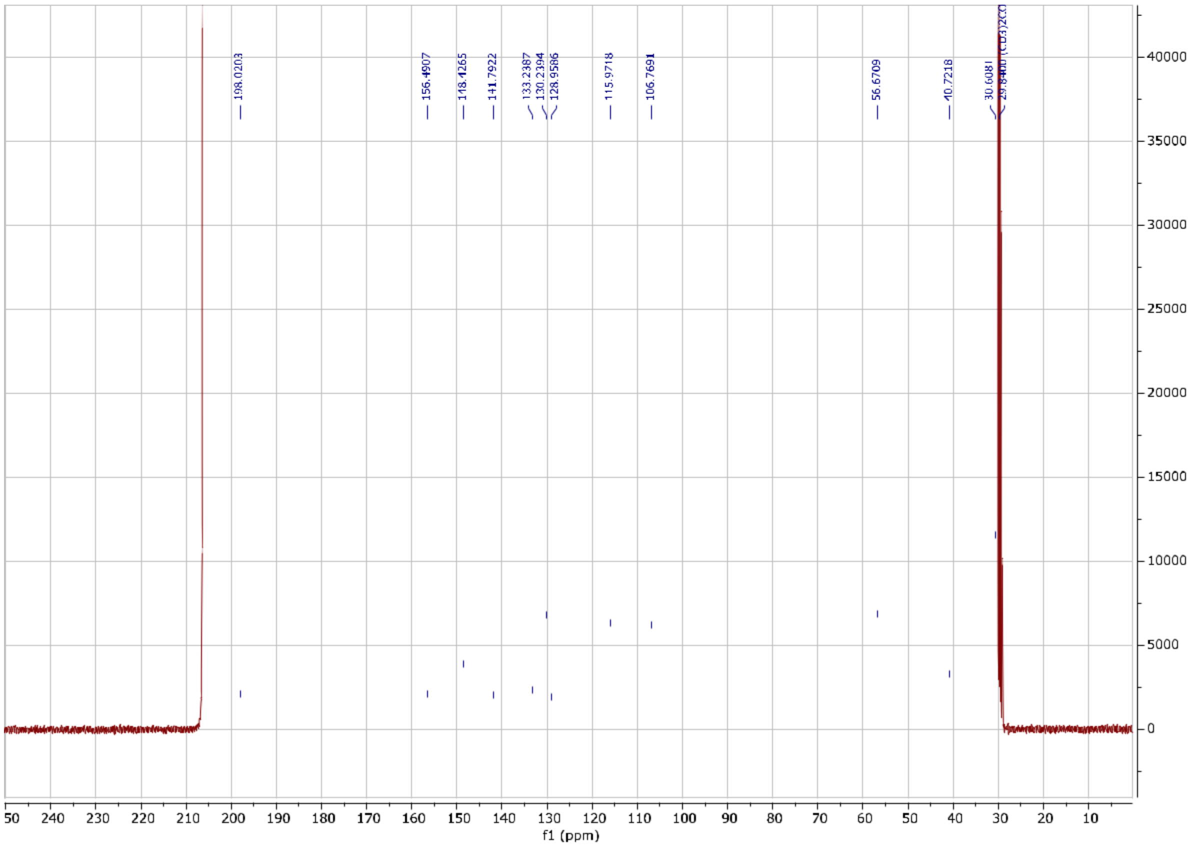
198.0203
156.4907
148.4265
141.7922
133.2387
130.2394
128.9586
115.9718
106.7691
56.6709
40.7218
30.6081
f1 (ppm)

b10

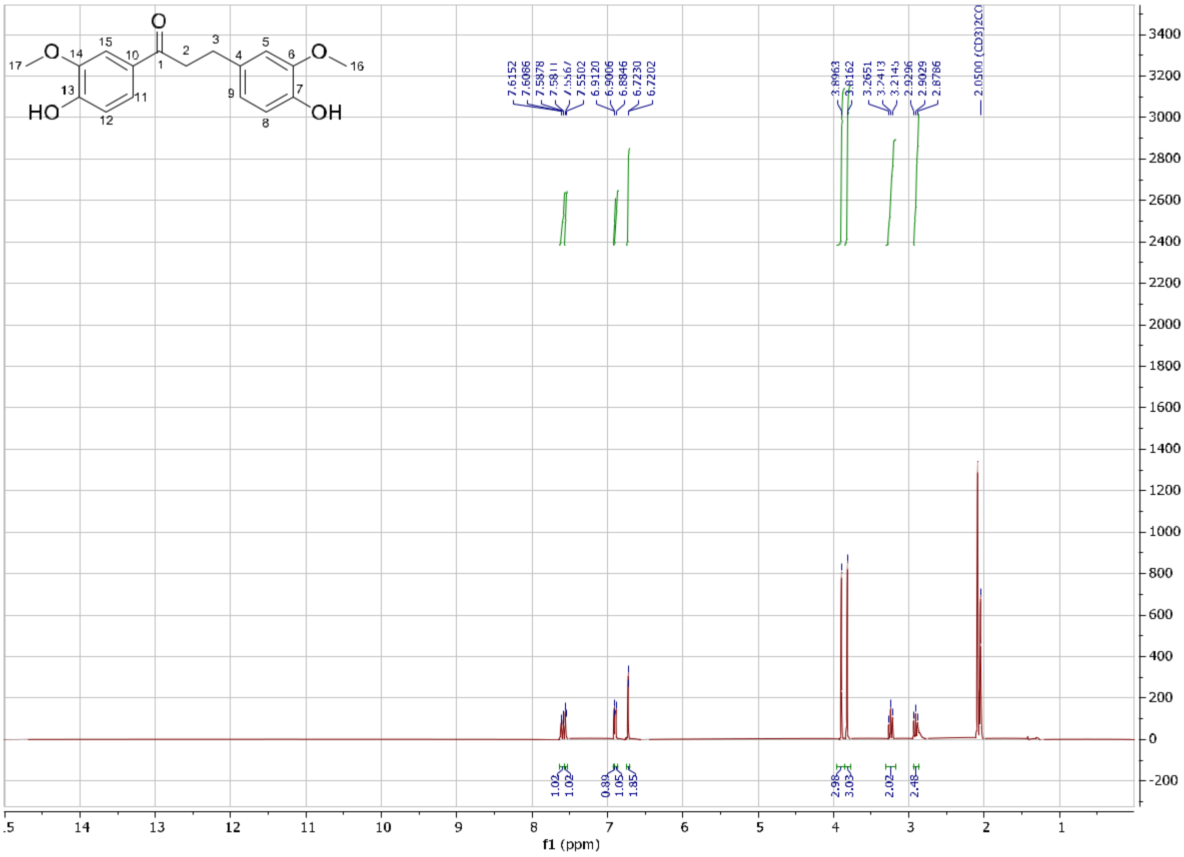

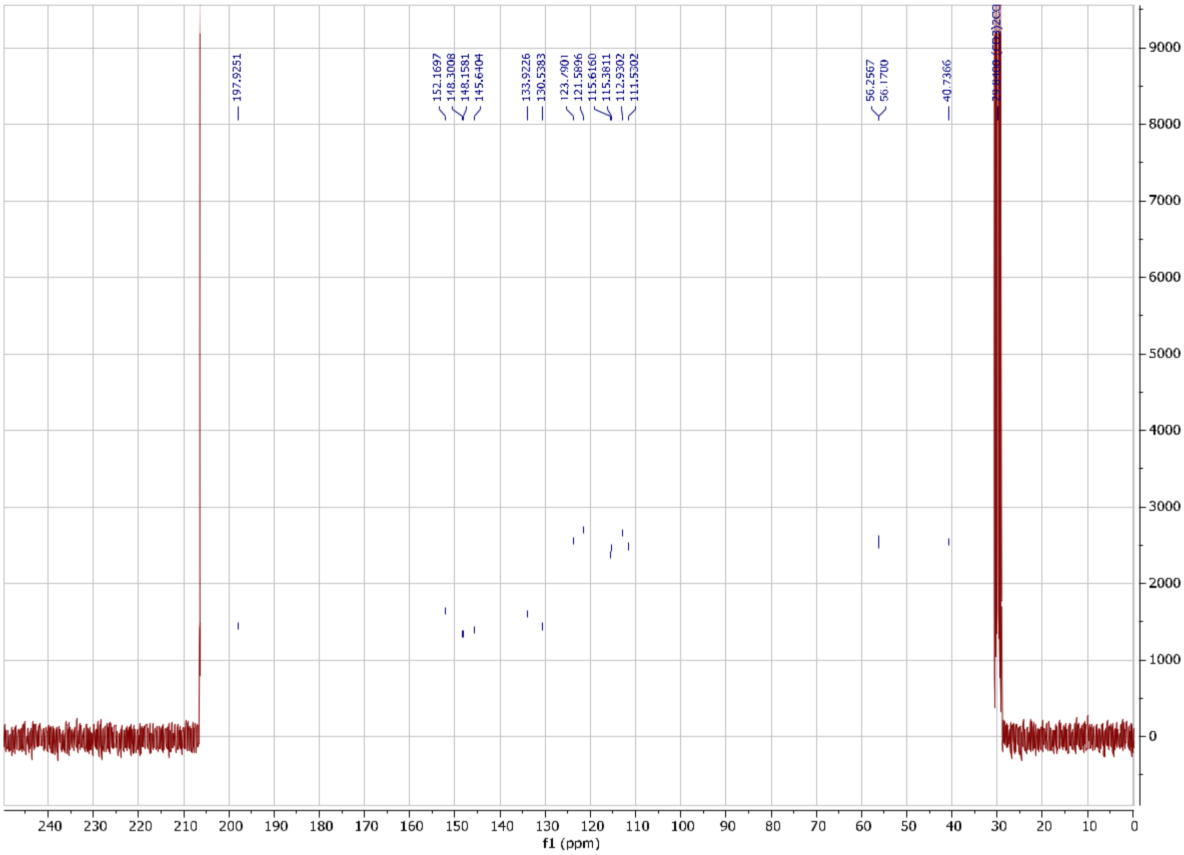

b11

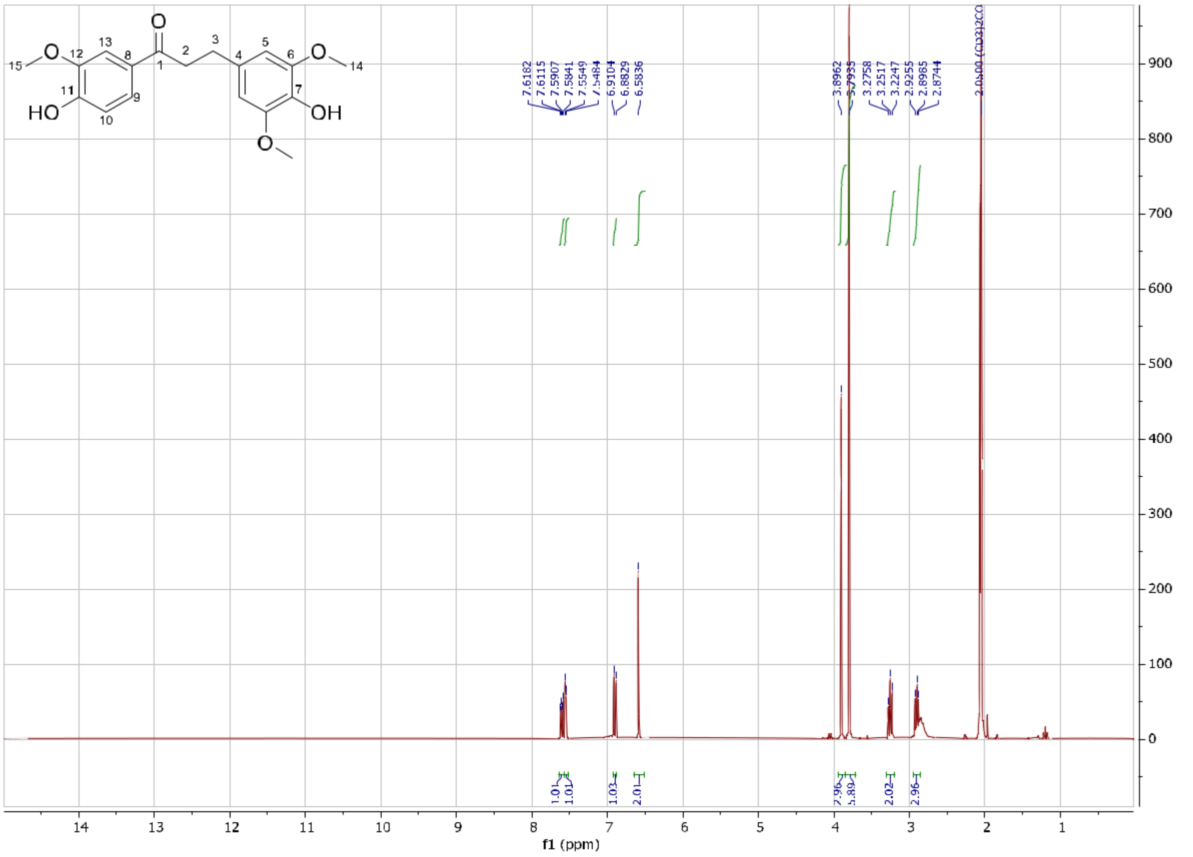
O
15
12
13
8
1
2
3
4
5
6
14
11
9
10
7
HO
OH
7.6182
7.6115
7.5907
7.5841
7.5549
7.5484
6.9104
6.8829
6.5836
3.8962
3.7935
3.2758
3.2517
3.2247
2.9255
2.8985
2.8744
2.0500 (CD3)2CO
1.01
1.01
1.03
2.01
2.96
5.89
2.02
2.96
f1 (ppm)

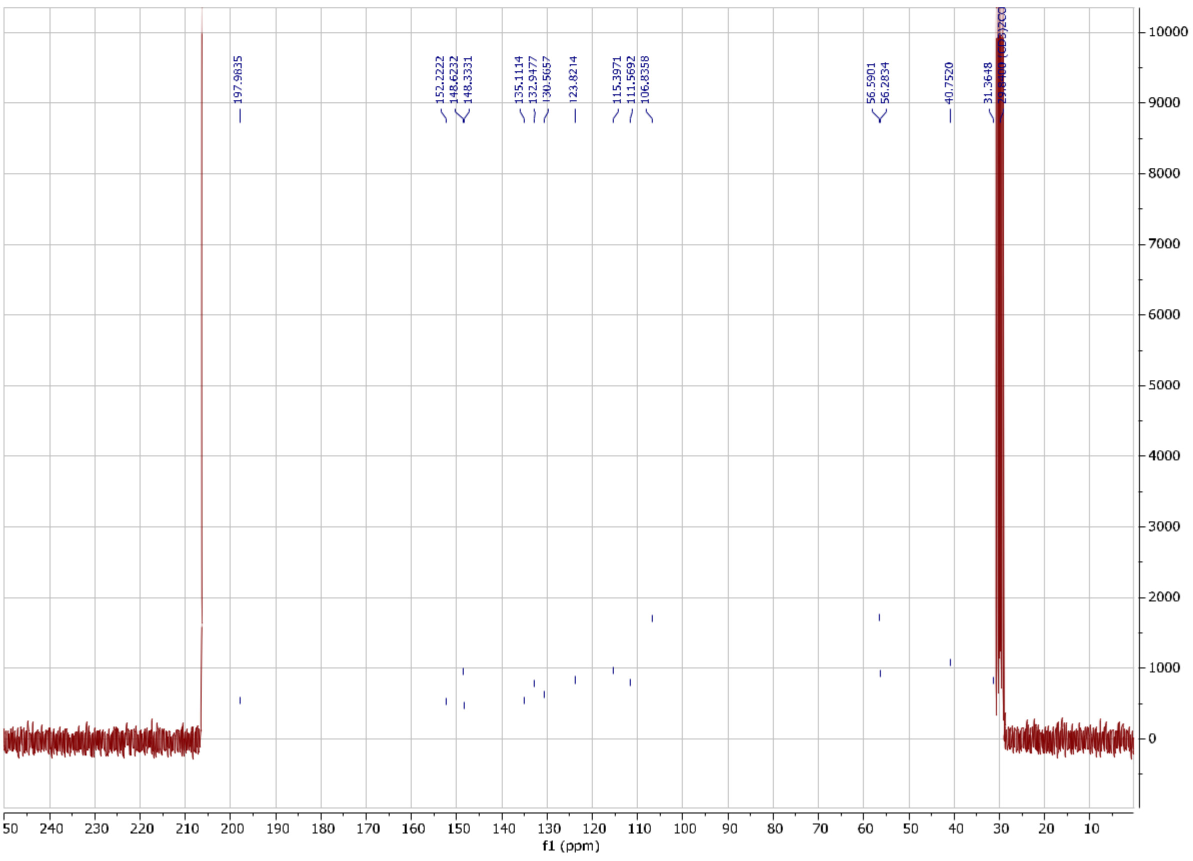
197.9835
152.2222
148.6232
148.3331
135.1114
132.9477
130.5657
123.8214
115.3971
111.5692
106.8358
56.5901
56.2834
40.7520
31.3648
f1 (ppm)

b12

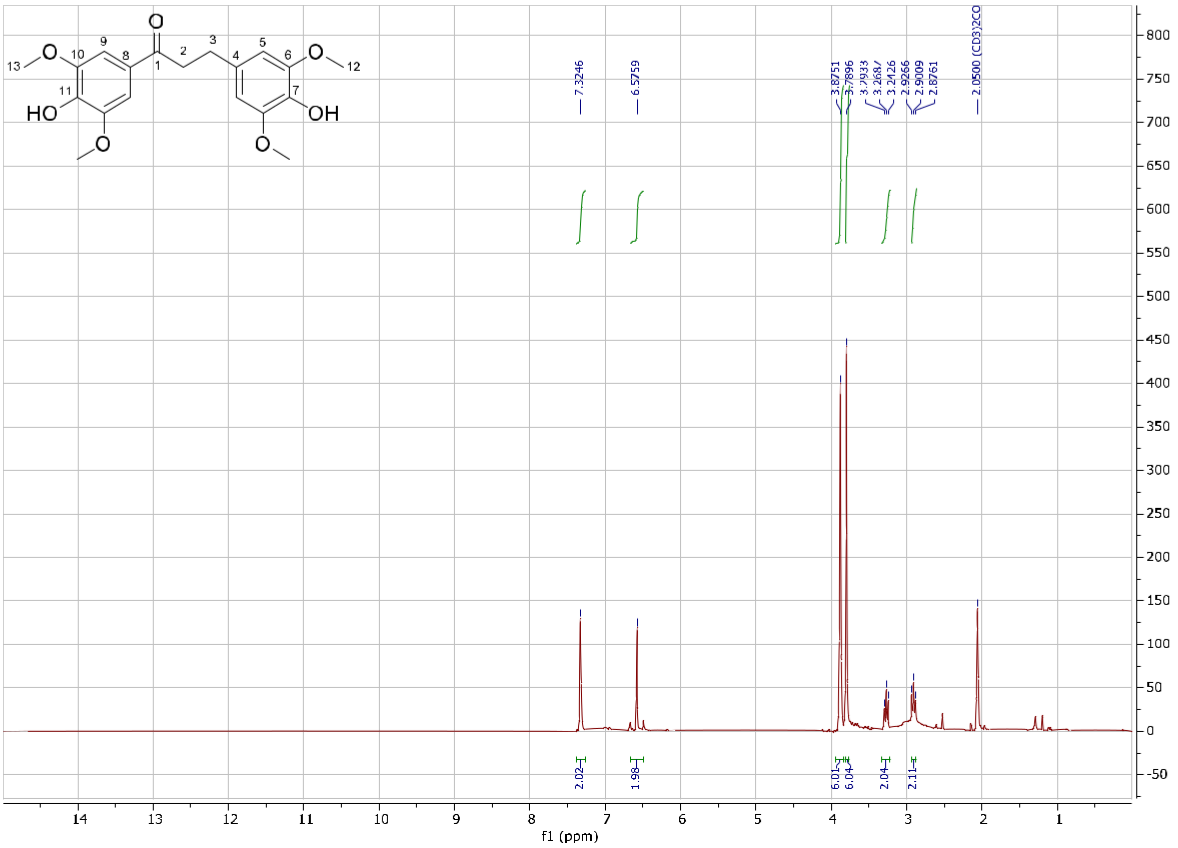
7.3246
6.5759
3.8751
3.7896
3.2933
3.2687
3.2426
2.9266
2.9009
2.8761
2.0500 (CD3)2CO
2.02
1.98
6.01
6.04
2.04
2.11
f1 (ppm)

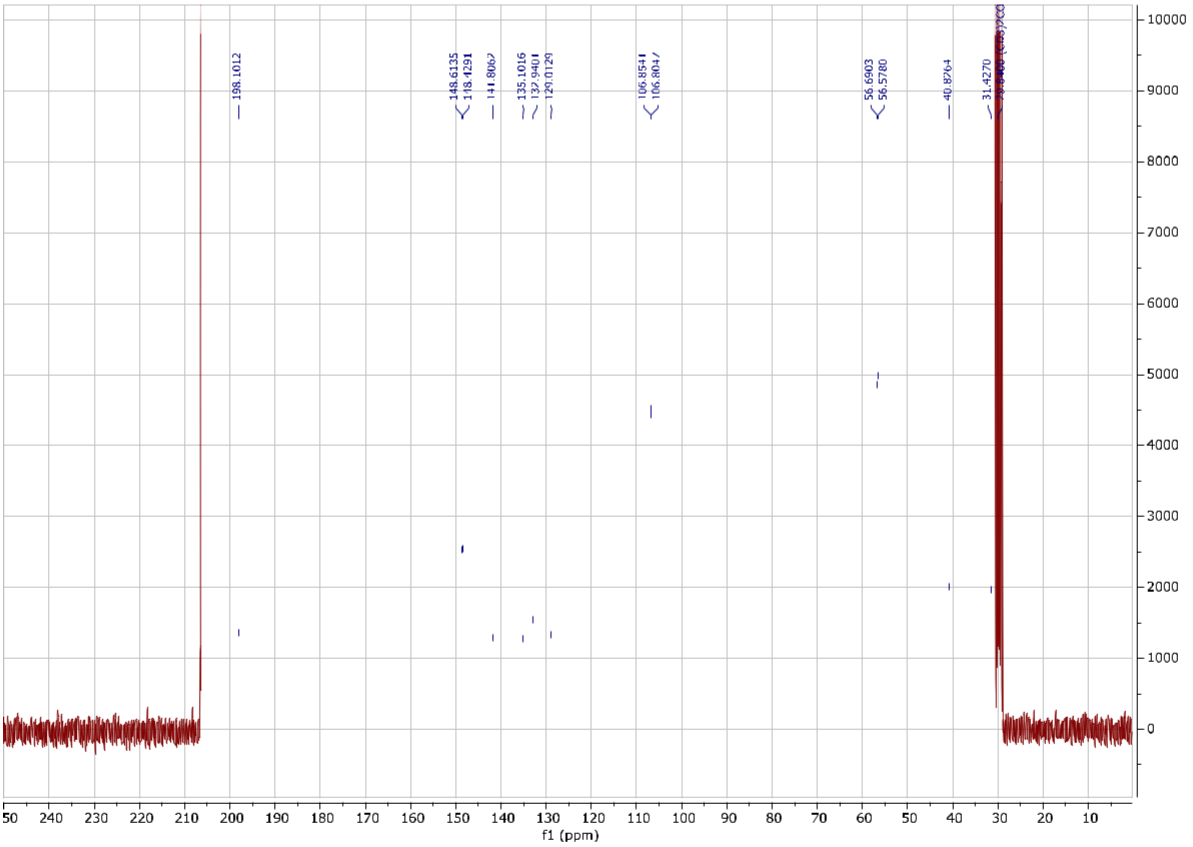
198.1012
148.6135
148.4291
141.8062
135.1016
132.9401
129.0129
106.8541
106.8047
56.6903
56.5780
40.8764
31.4270
f1 (ppm)

3. Tyrosinase inhibition

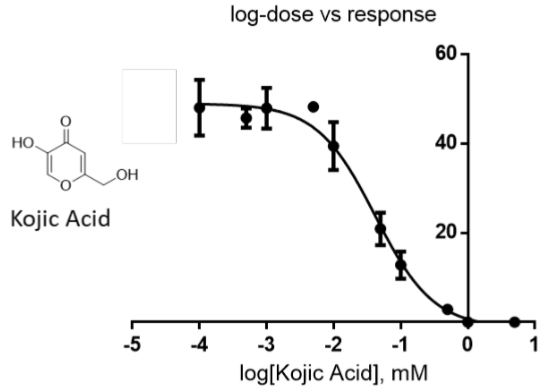

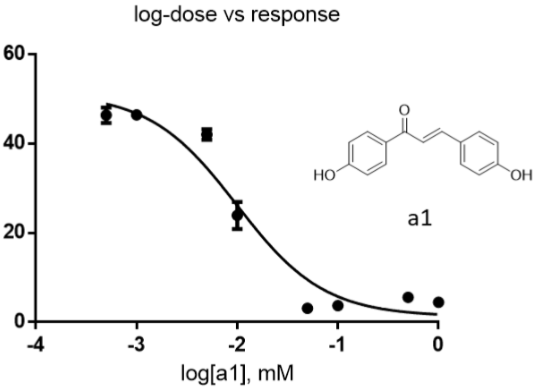

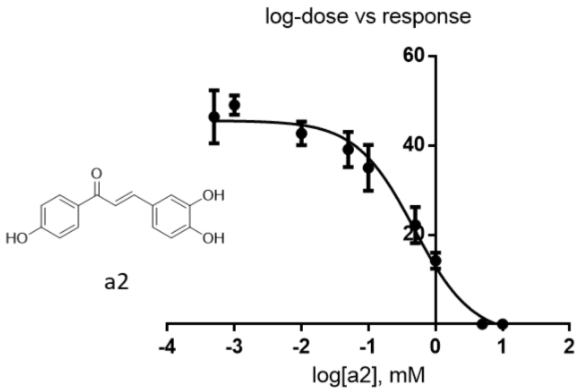

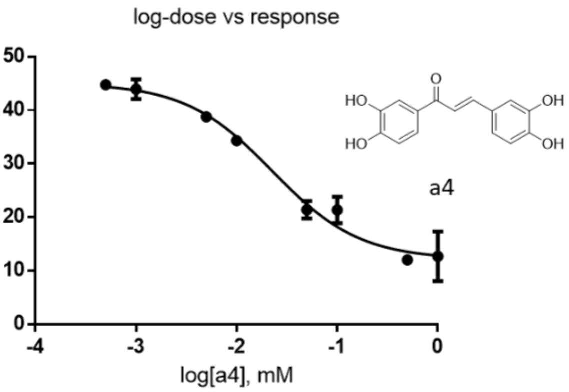

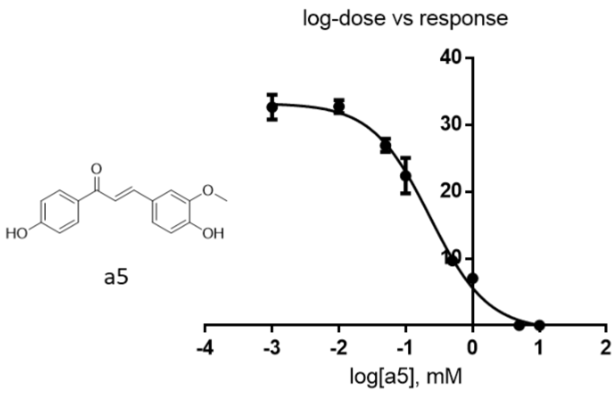

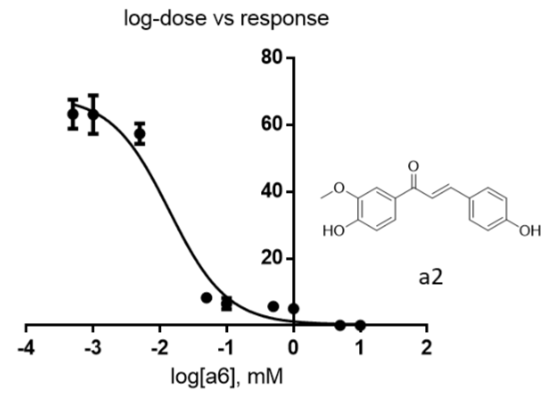

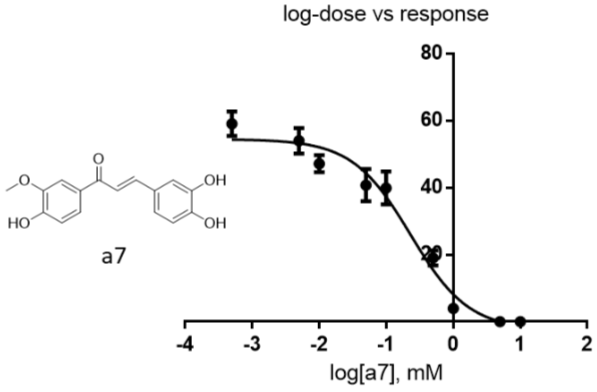

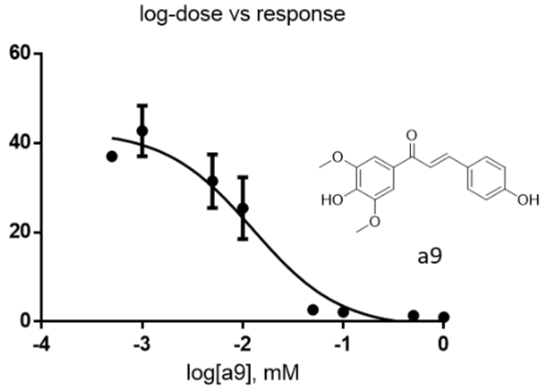

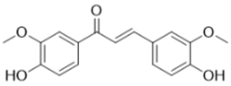

a10

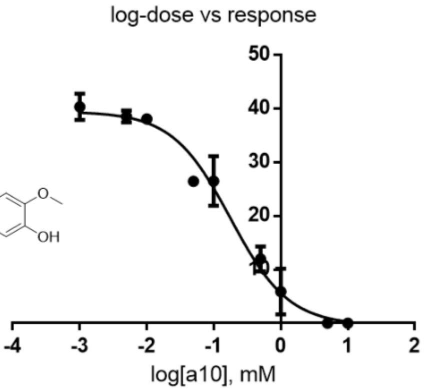

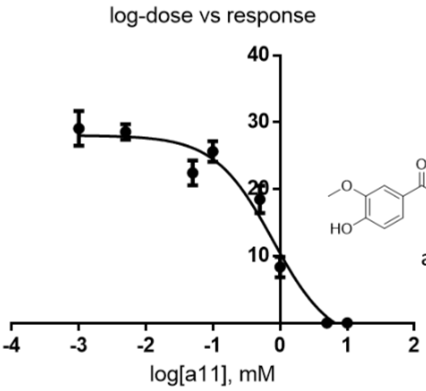

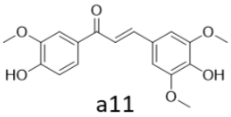

a11

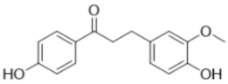

b5

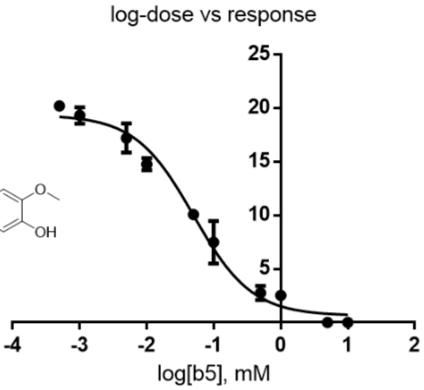

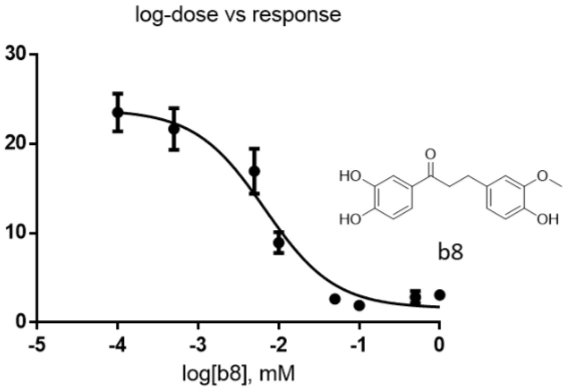

b8

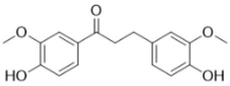

b10

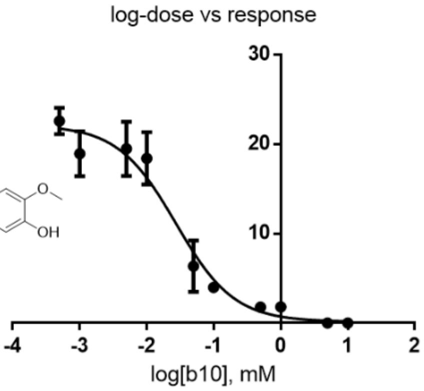

4. Antioxidant activity

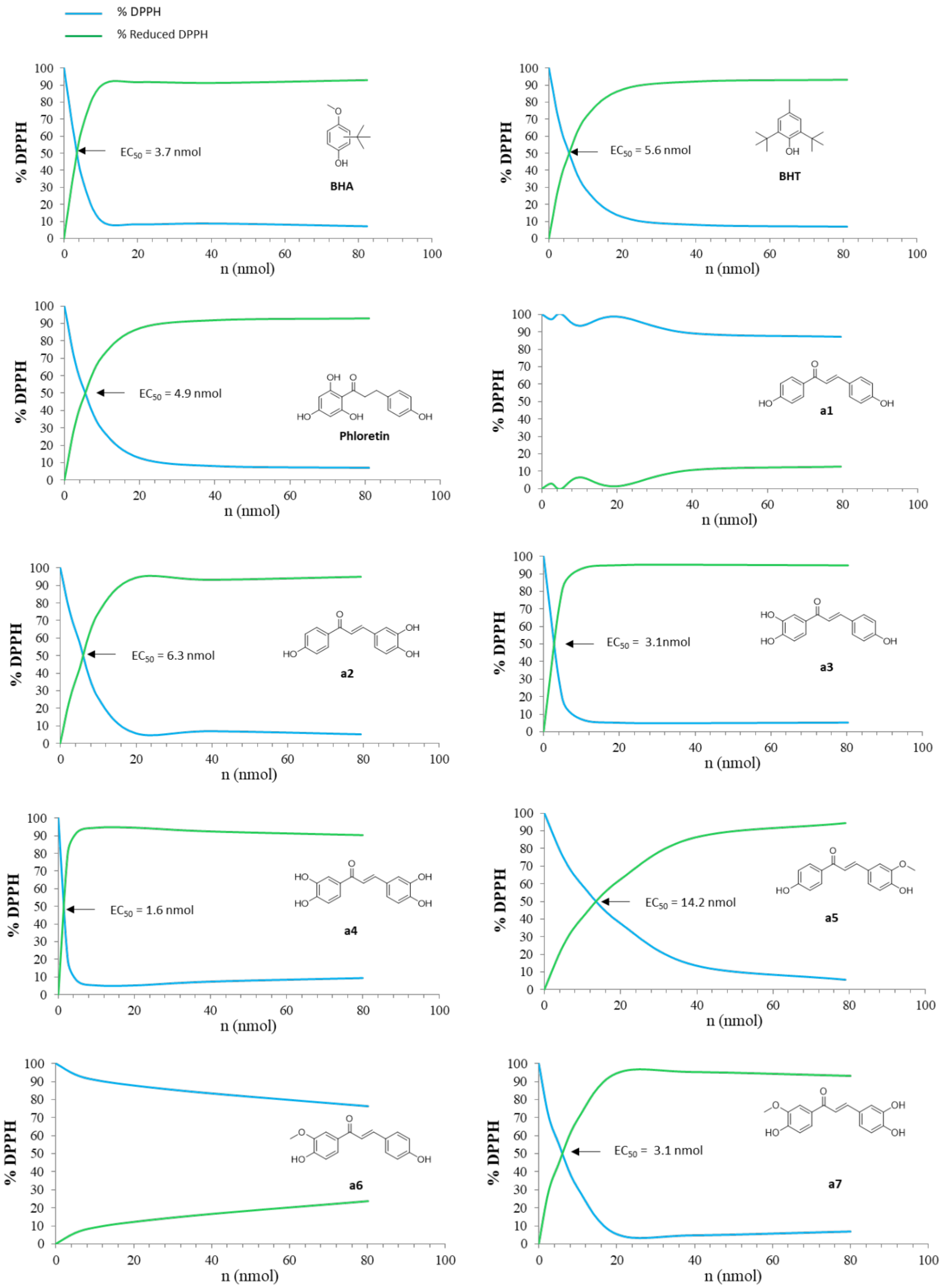

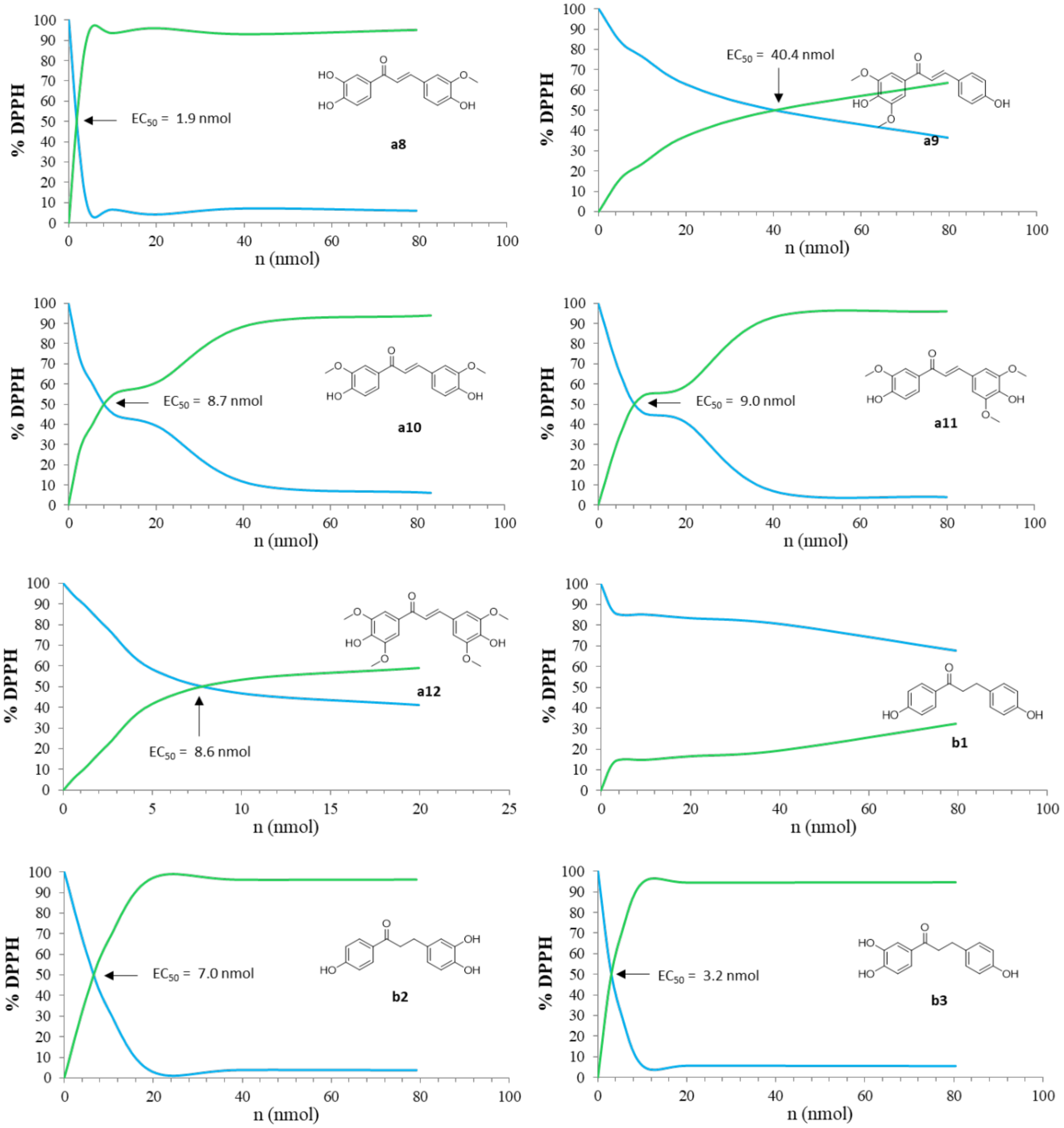

a8
EC50 = 1.9 nmol
a9
EC50 = 40.4 nmol
a10
EC50 = 8.7 nmol
a11
EC50 = 9.0 nmol
a12
EC50 = 8.6 nmol
b1
b2
EC50 = 7.0 nmol
b3
EC50 = 3.2 nmol
% DPPH
n (nmol)

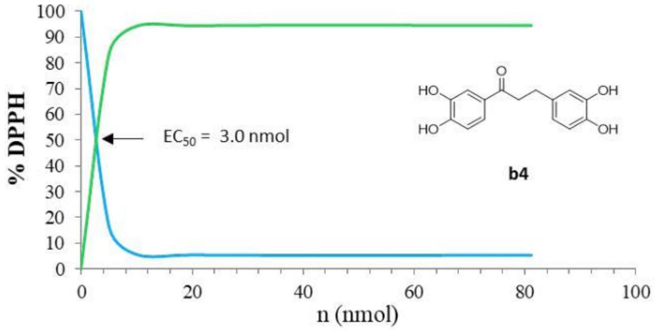
% DPPH
n (nmol)
EC_{50} = 3.0 nmol
b4

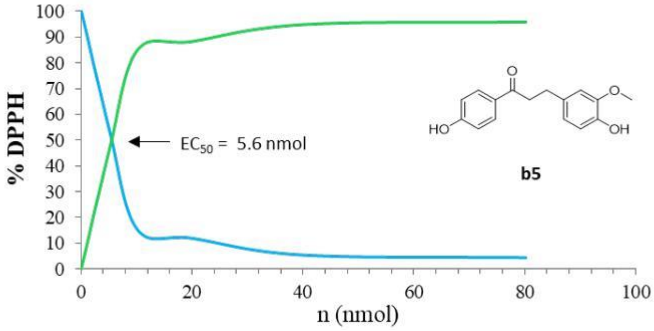
% DPPH
n (nmol)
EC_{50} = 5.6 nmol
b5

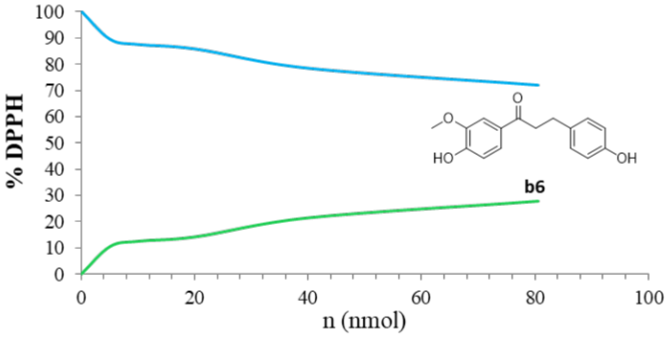
% DPPH
n (nmol)
b6

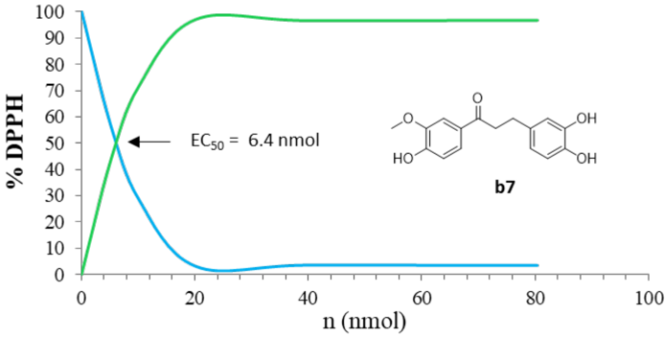
% DPPH
n (nmol)
EC_{50} = 6.4 nmol
b7

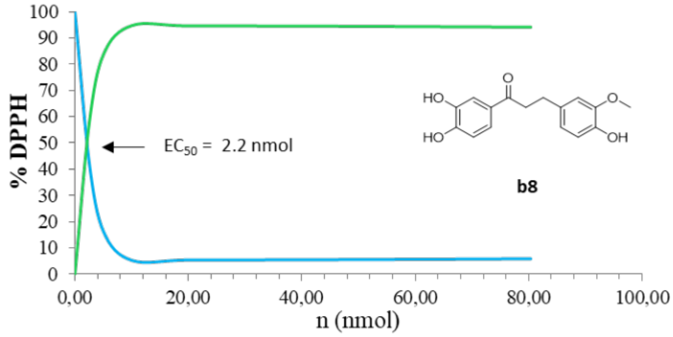
% DPPH
n (nmol)
EC_{50} = 2.2 nmol
b8

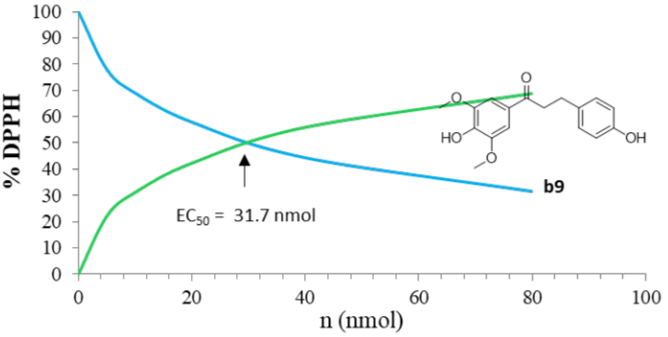
% DPPH
n (nmol)
EC_{50} = 31.7 nmol
b9

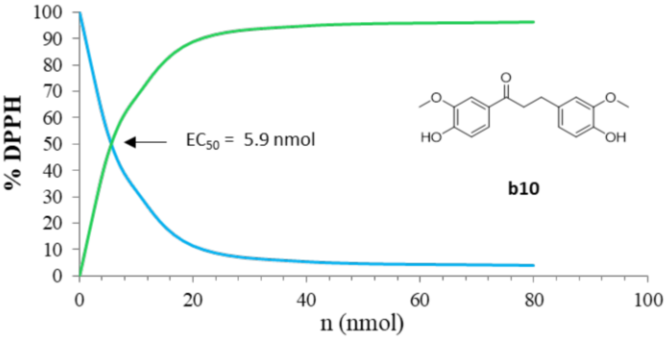
% DPPH
n (nmol)
EC_{50} = 5.9 nmol
b10

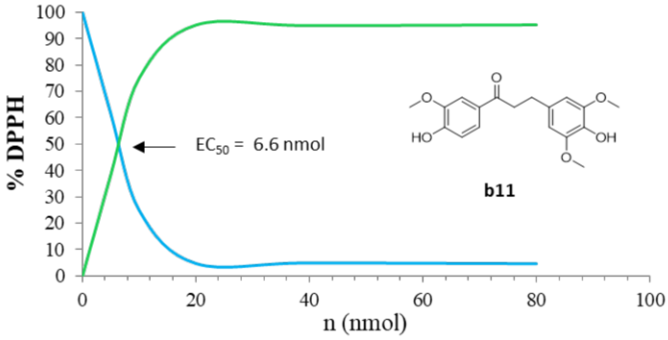
% DPPH
n (nmol)
EC_{50} = 6.6 nmol
b11

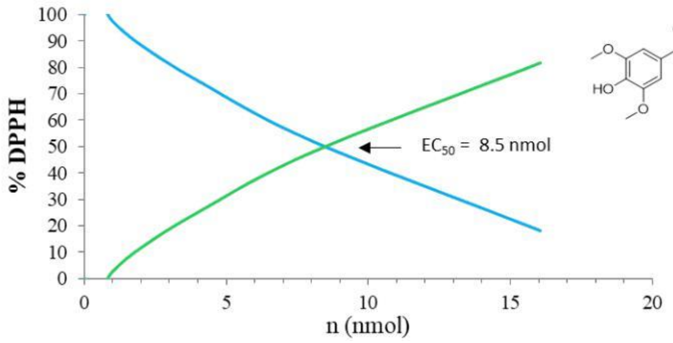

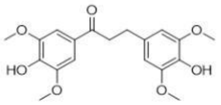

b12

5. UV Filter activity

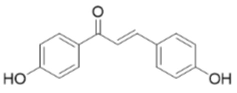

a1

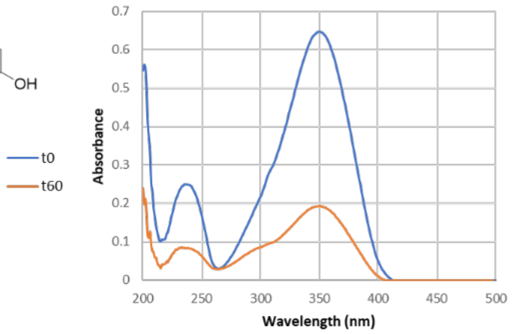

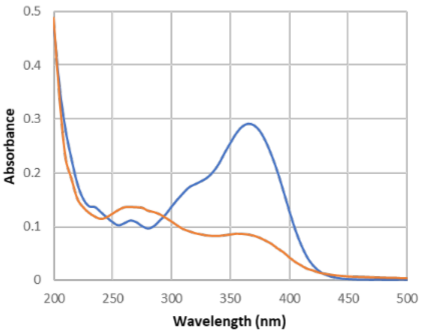

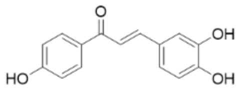

a2

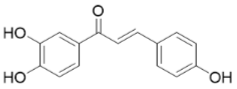

a3

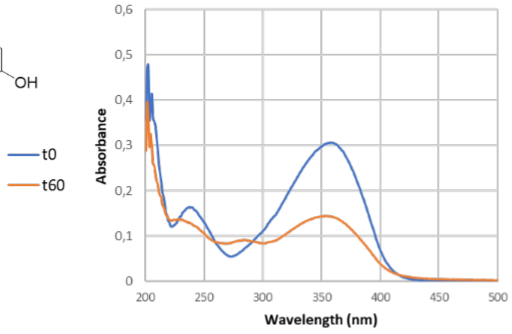

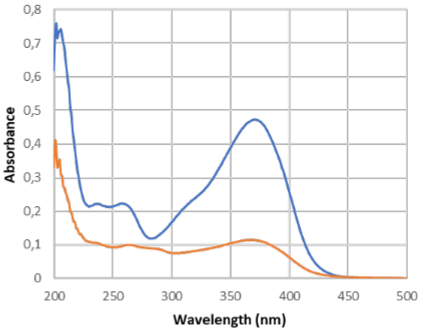

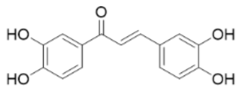

a4

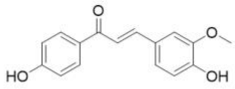

a5

t0
t60

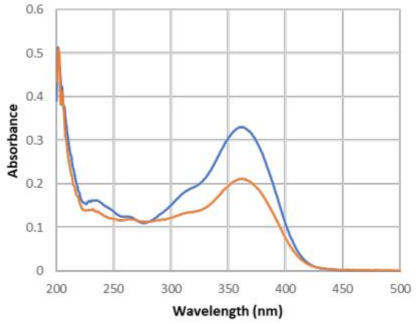

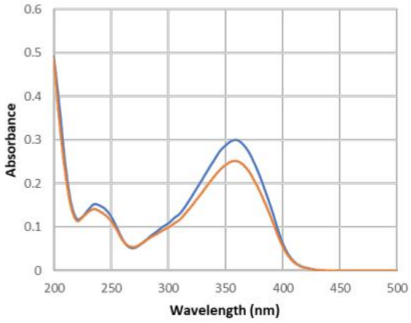

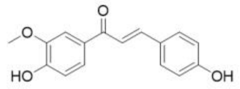

a6

t0
t60

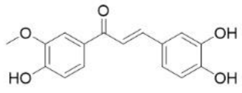

a7

t0
t60

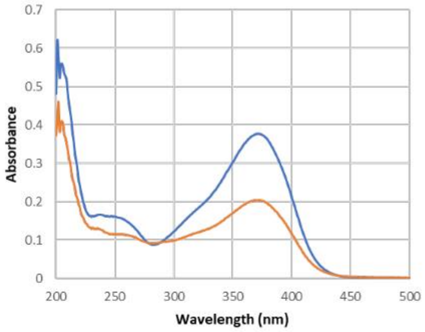

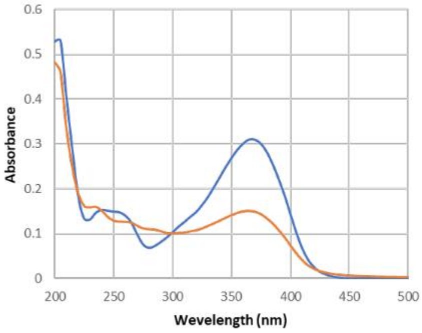

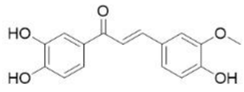

a8

t0
t60

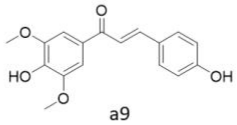

a9

t0
t60

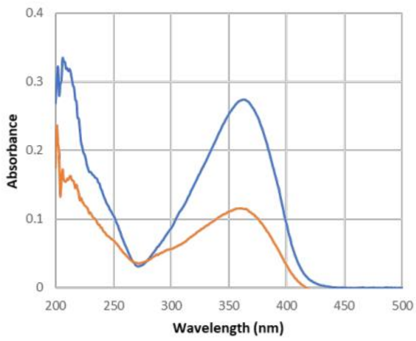

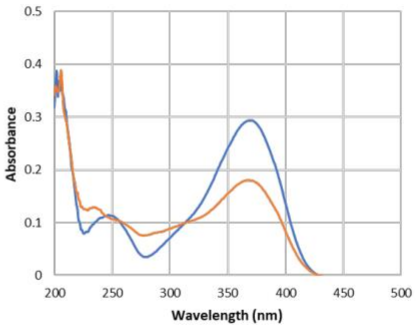

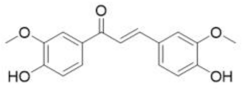

a10

t0
t60

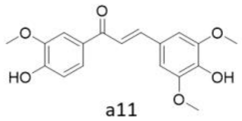

a11

t0
t60

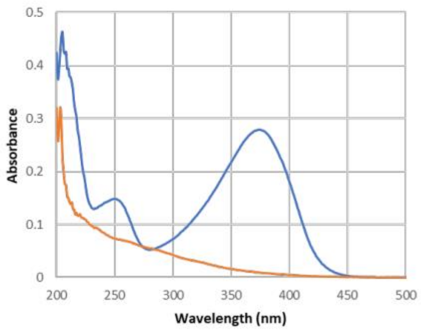

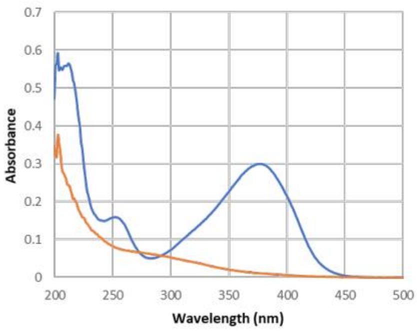

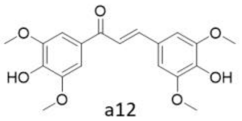

a12

t0
t60

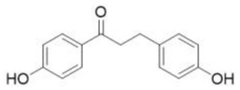

b1

t0
t60

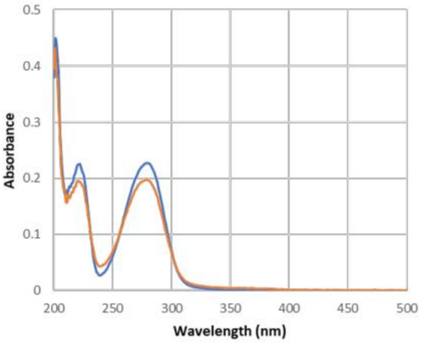

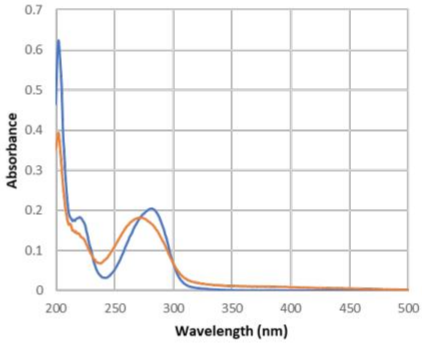

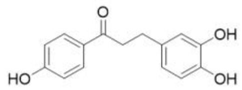

b2

t0
t60

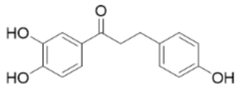

b3

t0
t60

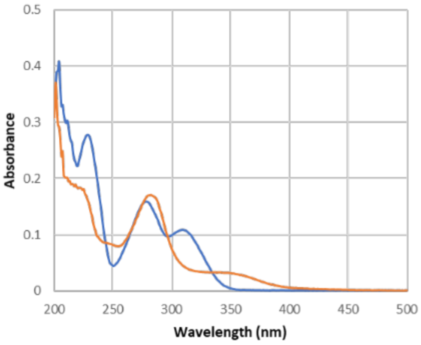

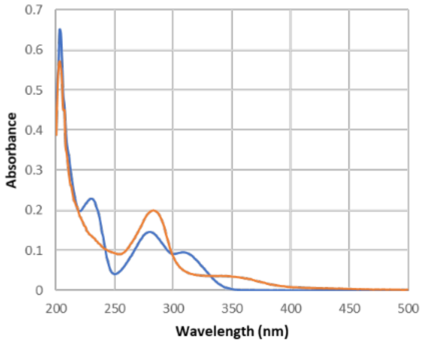

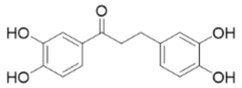

b4

t0
t60

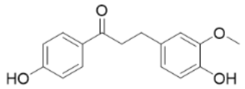

b5

t0
t60

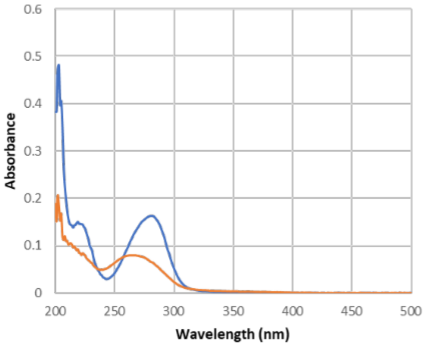

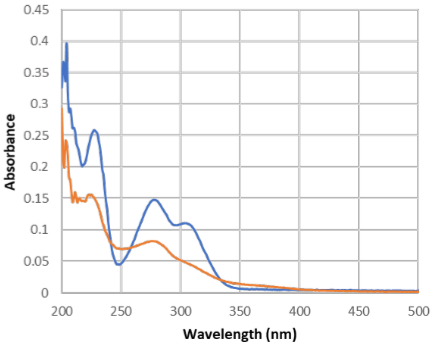

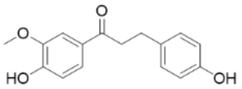

b6

t0
t60

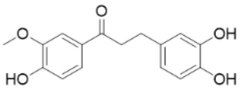

b7

t0
t60

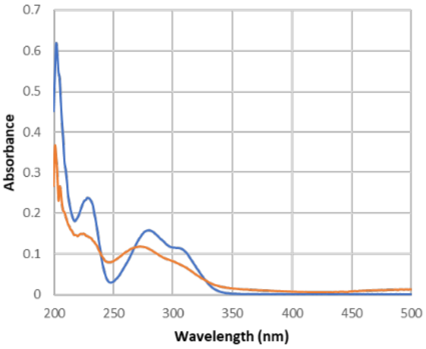

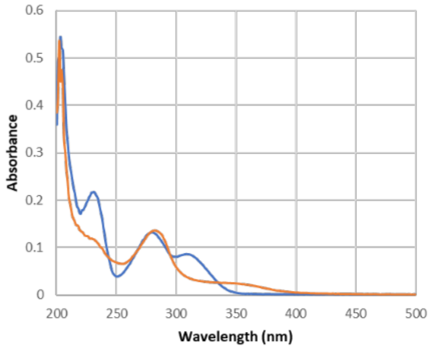

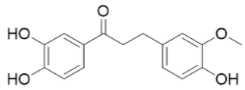

b8

t0
t60

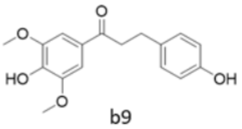

b9

t0
t60

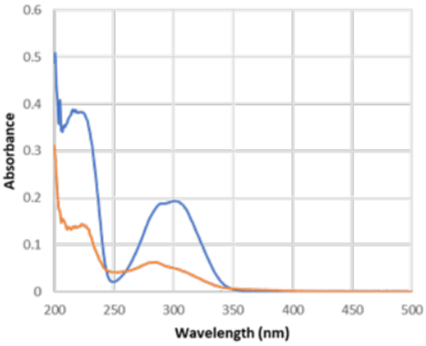

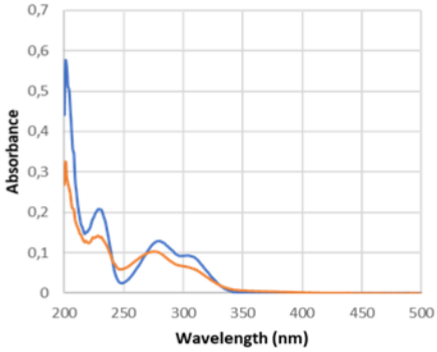

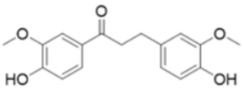

b10

t0
t60

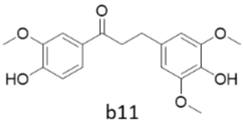

b11

t0
t60

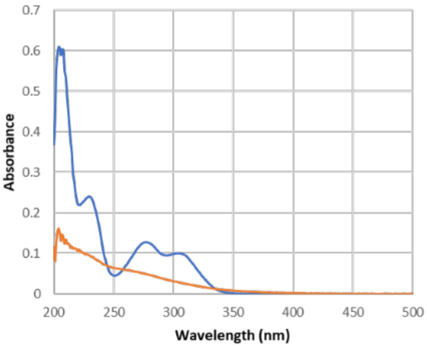

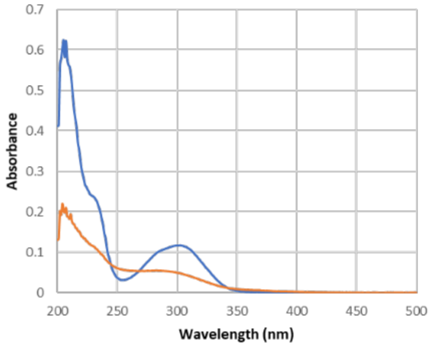

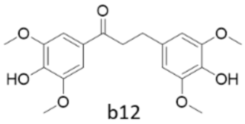

b12

t0
t60

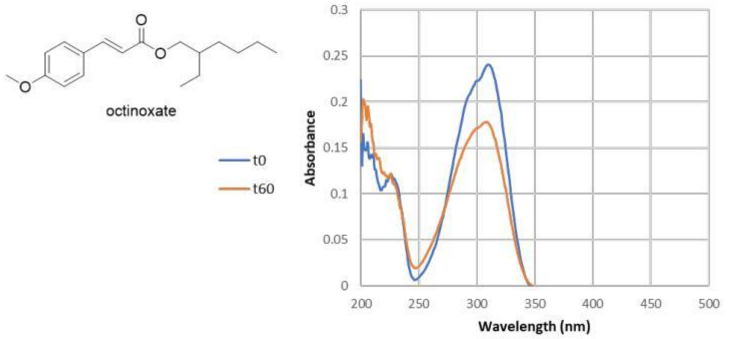

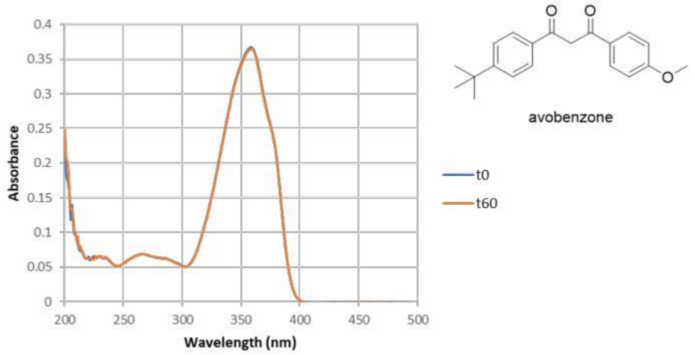

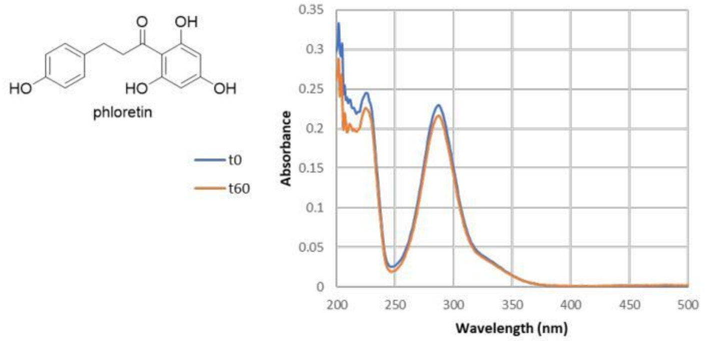

6. FT-IR spectra

a1

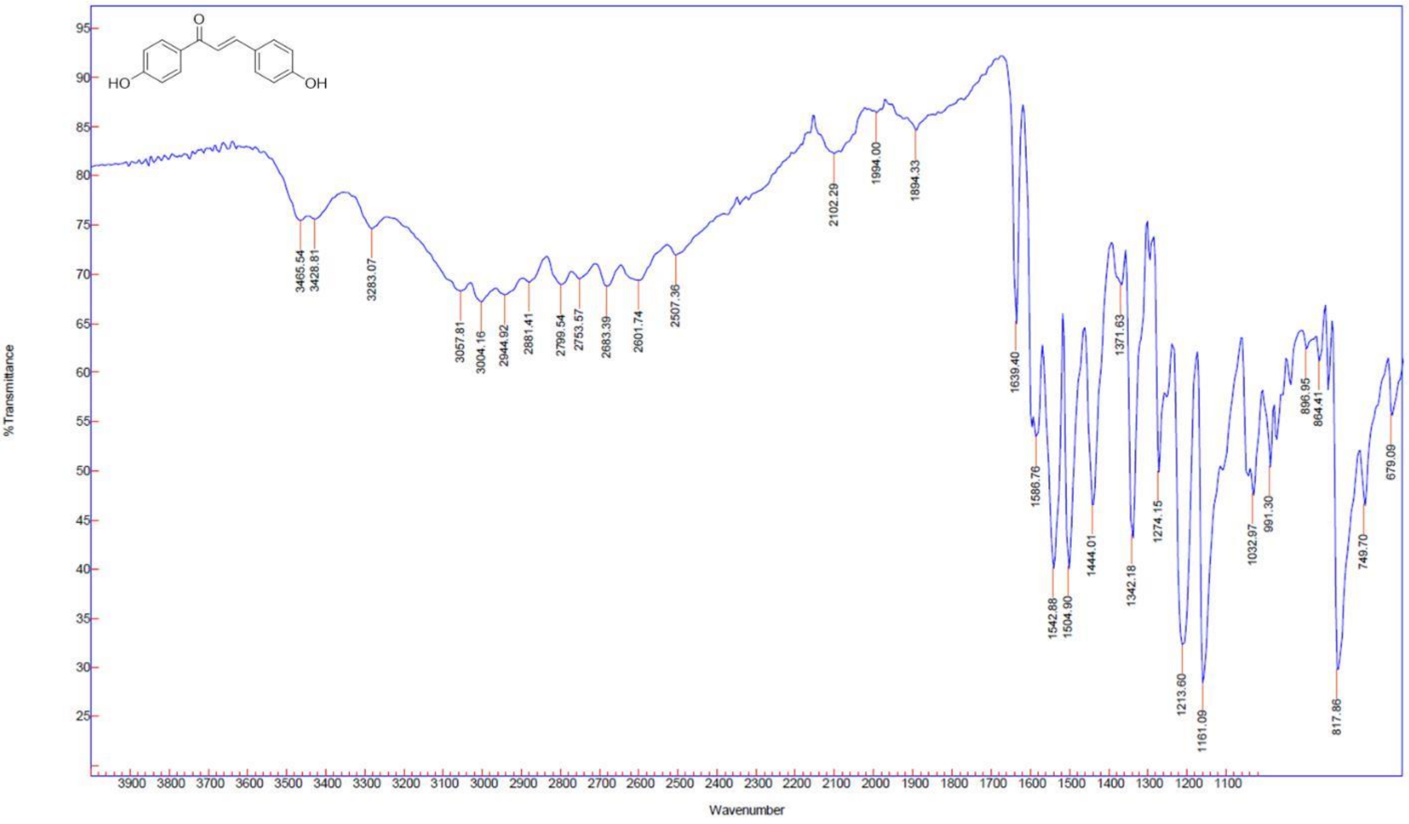

a2

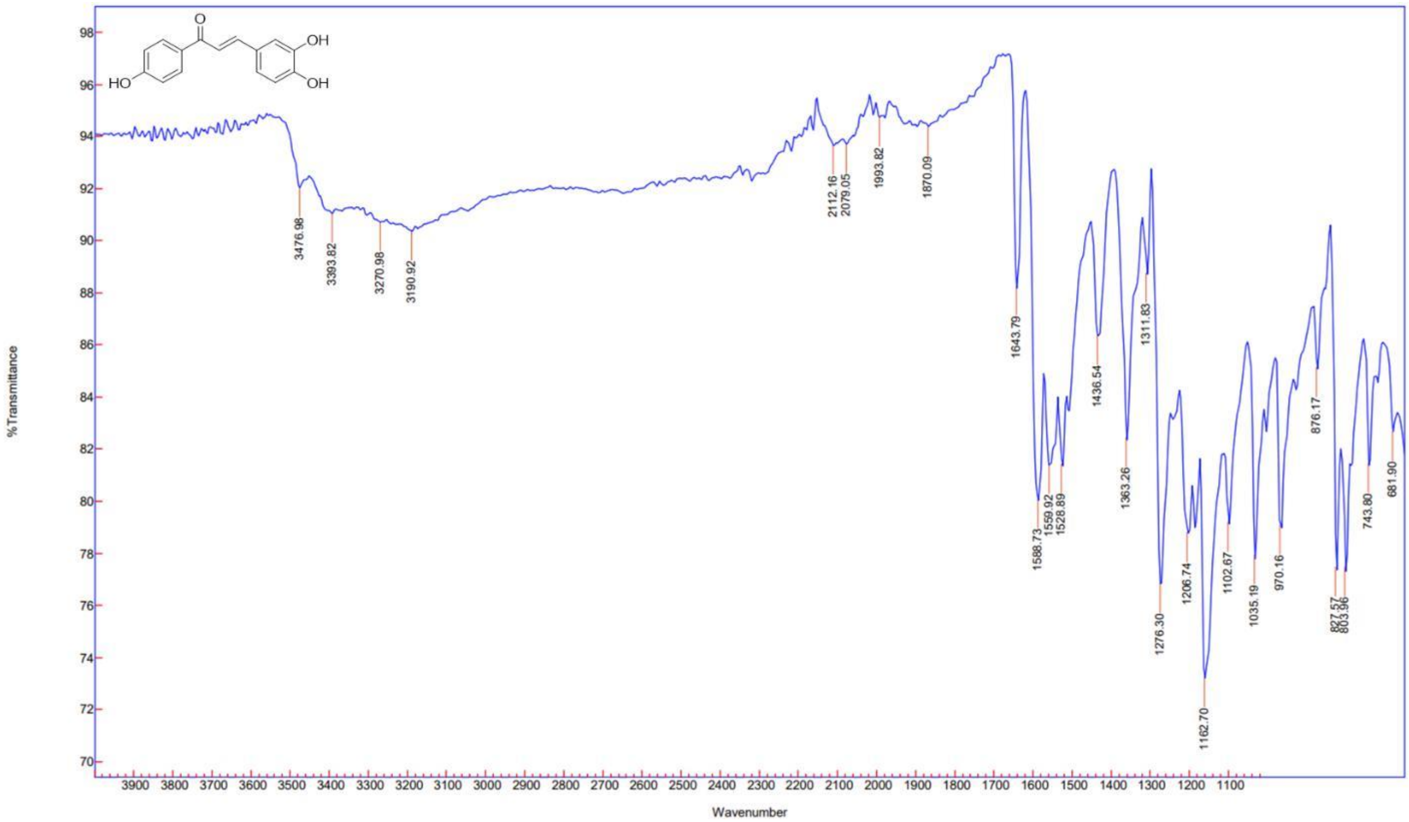

HO
OH
OH
O
% Transmittance
Wavenumber
3476.98
3393.82
3270.98
3190.92
2112.16
2079.05
1993.82
1870.09
1643.79
1588.73
1559.92
1528.89
1436.54
1363.26
1311.83
1276.30
1206.74
1162.70
1102.67
1035.19
970.16
876.17
827.57
803.96
743.80
681.90

a3

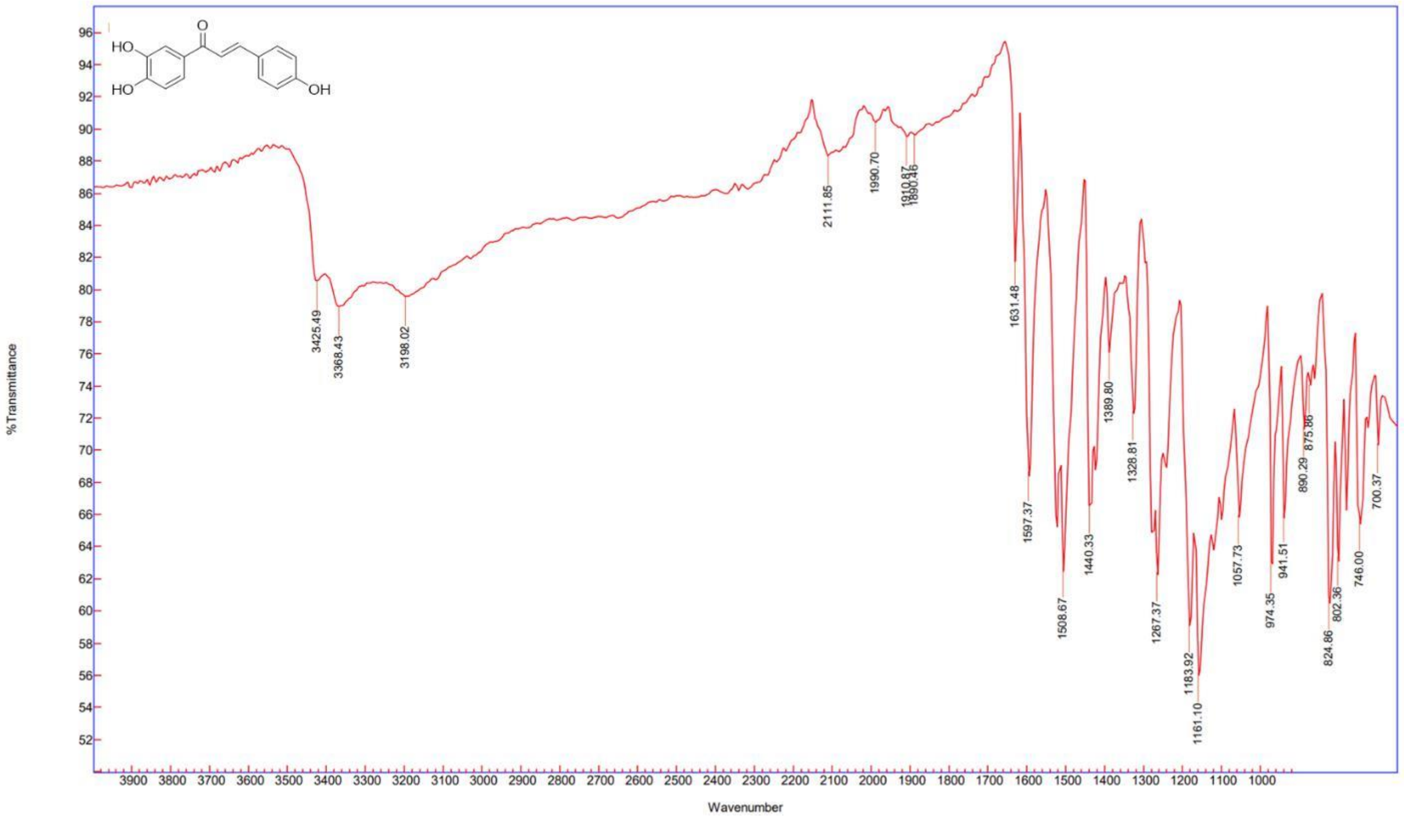

HO
HO
OH
O
% Transmittance
Wavenumber
3425.49
3368.43
3198.02
2111.85
1990.70
1910.87
1890.46
1631.48
1597.37
1508.67
1440.33
1389.80
1328.81
1267.37
1183.92
1161.10
1057.73
974.35
941.51
890.29
875.86
824.86
802.36
746.00
700.37

a4

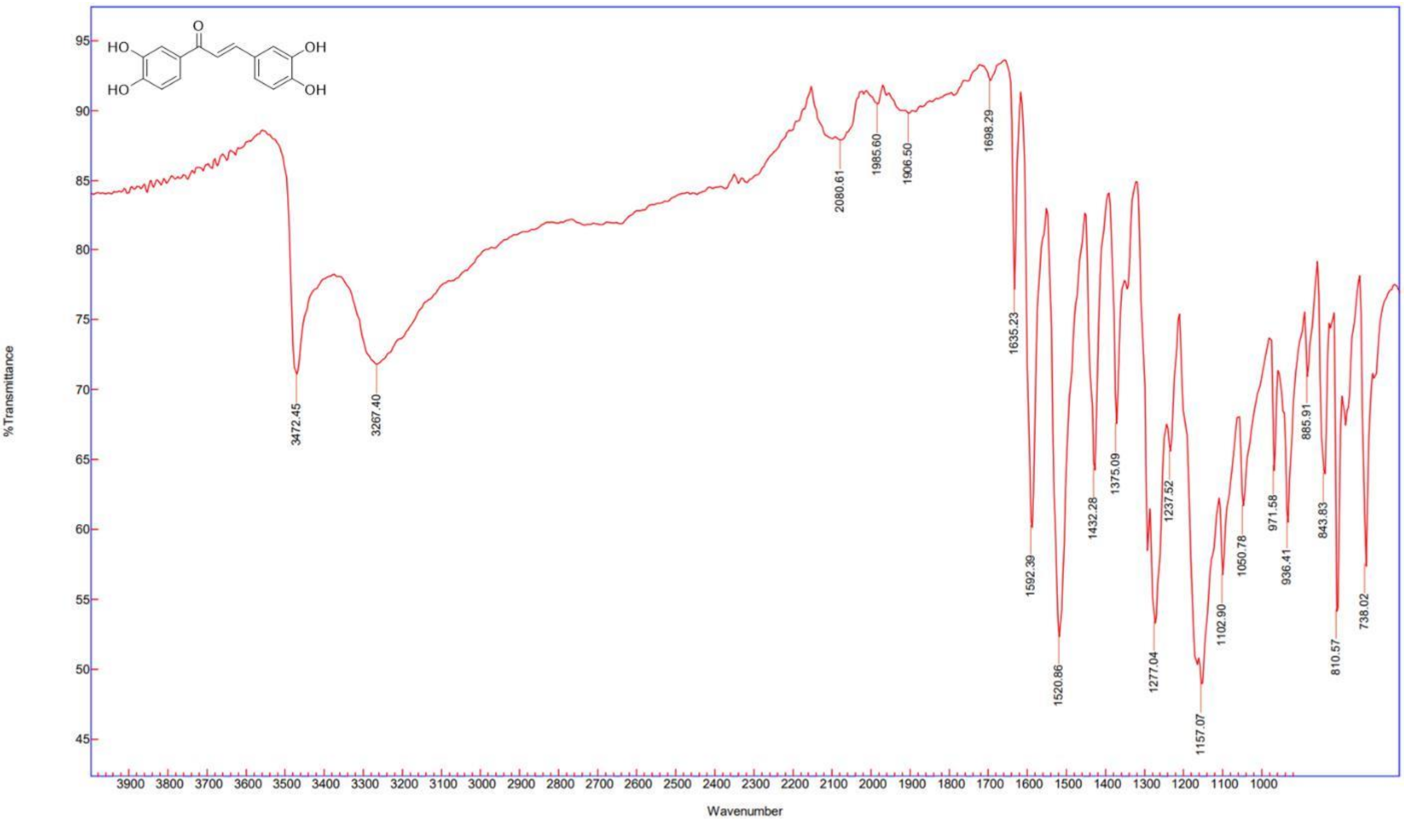

HO
HO
O
OH
OH
%Transmittance
Wavenumber
3472.45
3267.40
2080.61
1985.60
1906.50
1698.29
1635.23
1592.39
1520.86
1432.28
1375.09
1277.04
1237.52
1157.07
1102.90
1050.78
971.58
936.41
885.91
843.83
810.57
738.02

a5

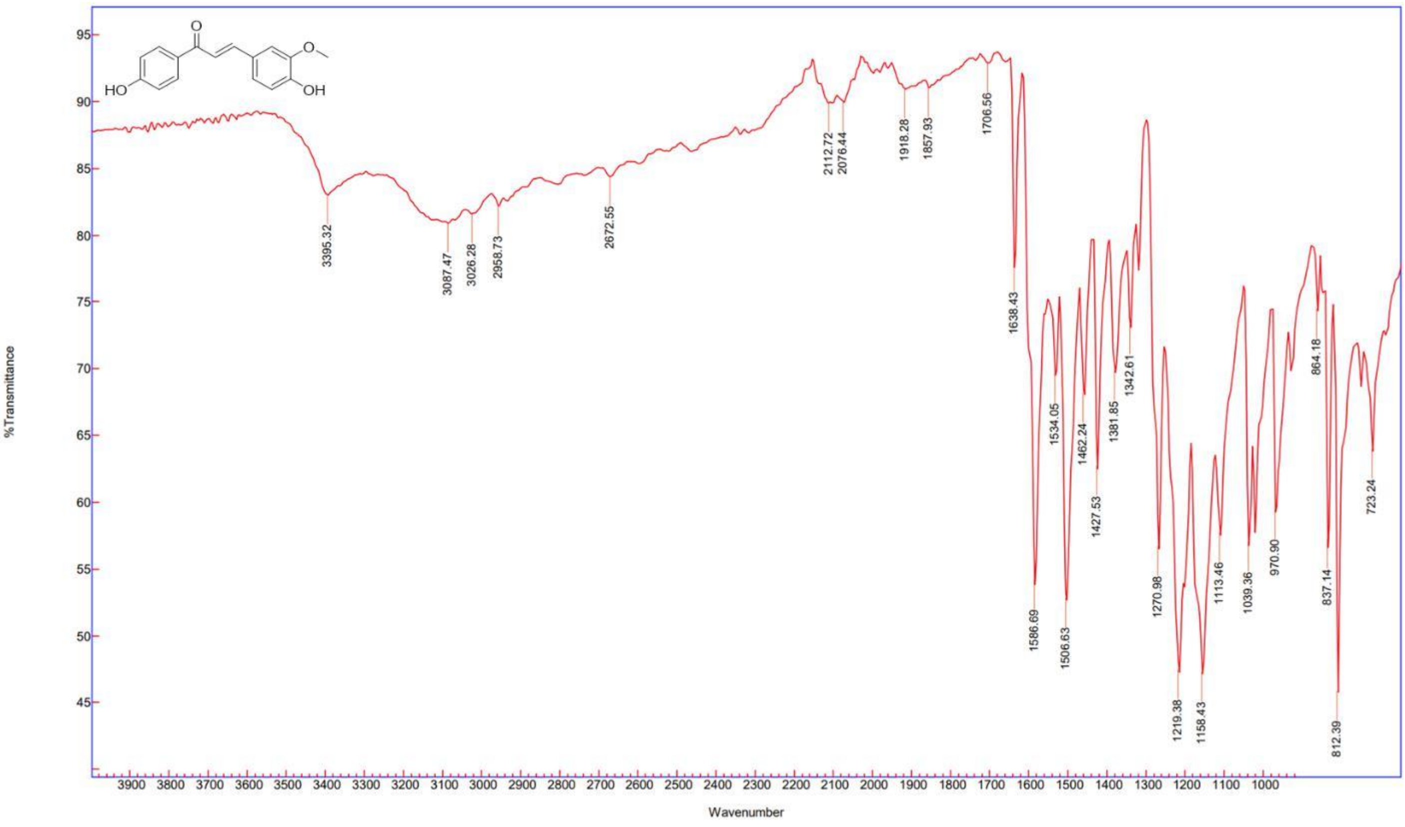

HO
O
O
OH
%Transmittance
Wavenumber
3395.32
3087.47
3026.28
2958.73
2672.55
2112.72
2076.44
1918.28
1857.93
1706.56
1638.43
1586.69
1534.05
1506.63
1462.24
1427.53
1381.85
1342.61
1270.98
1219.38
1158.43
1113.46
1039.36
970.90
864.18
837.14
812.39
723.24

a6

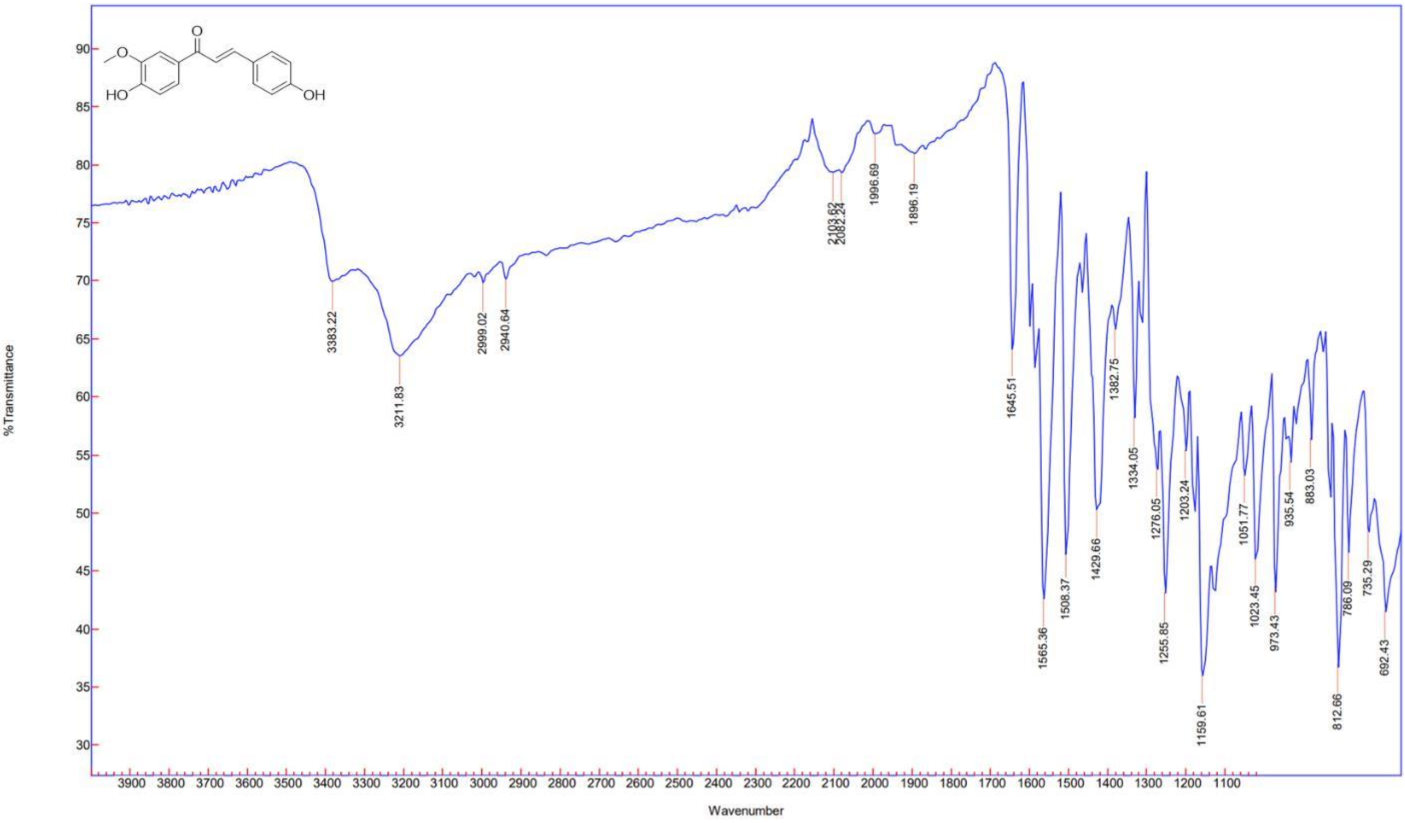
%Transmittance
Wavenumber
3383.22
3211.83
2999.02
2940.64
2103.62
2082.24
1996.69
1896.19
1645.51
1565.36
1508.37
1429.66
1382.75
1334.05
1276.05
1255.85
1203.24
1159.61
1051.77
1023.45
973.43
935.54
883.03
812.66
786.09
735.29
692.43

a7

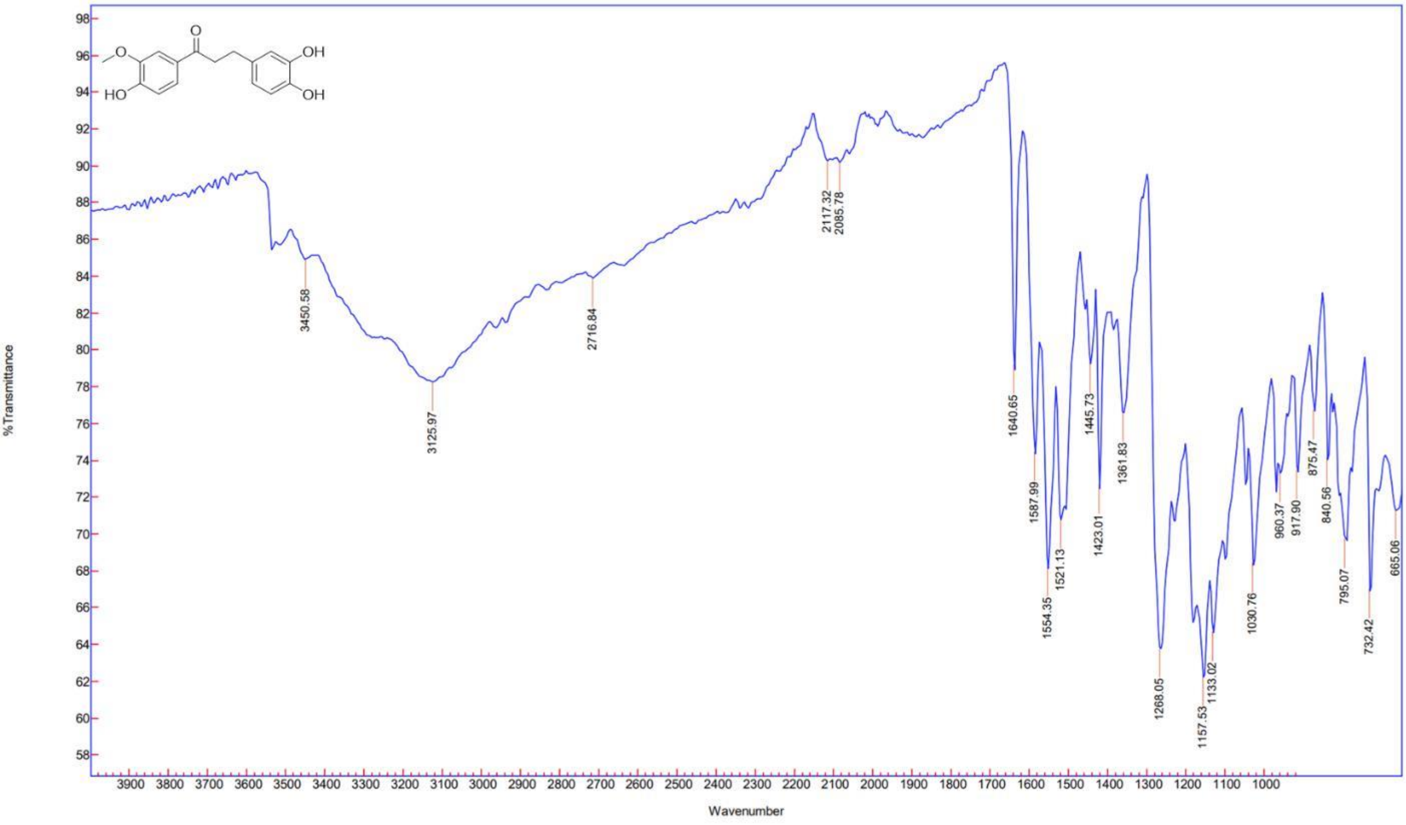
%Transmittance
Wavenumber
3450.58
3125.97
2716.84
2117.32
2085.78
1640.65
1587.99
1554.35
1521.13
1445.73
1423.01
1361.83
1268.05
1157.53
1133.02
1030.76
960.37
917.90
875.47
840.56
795.07
732.42
665.06

a8

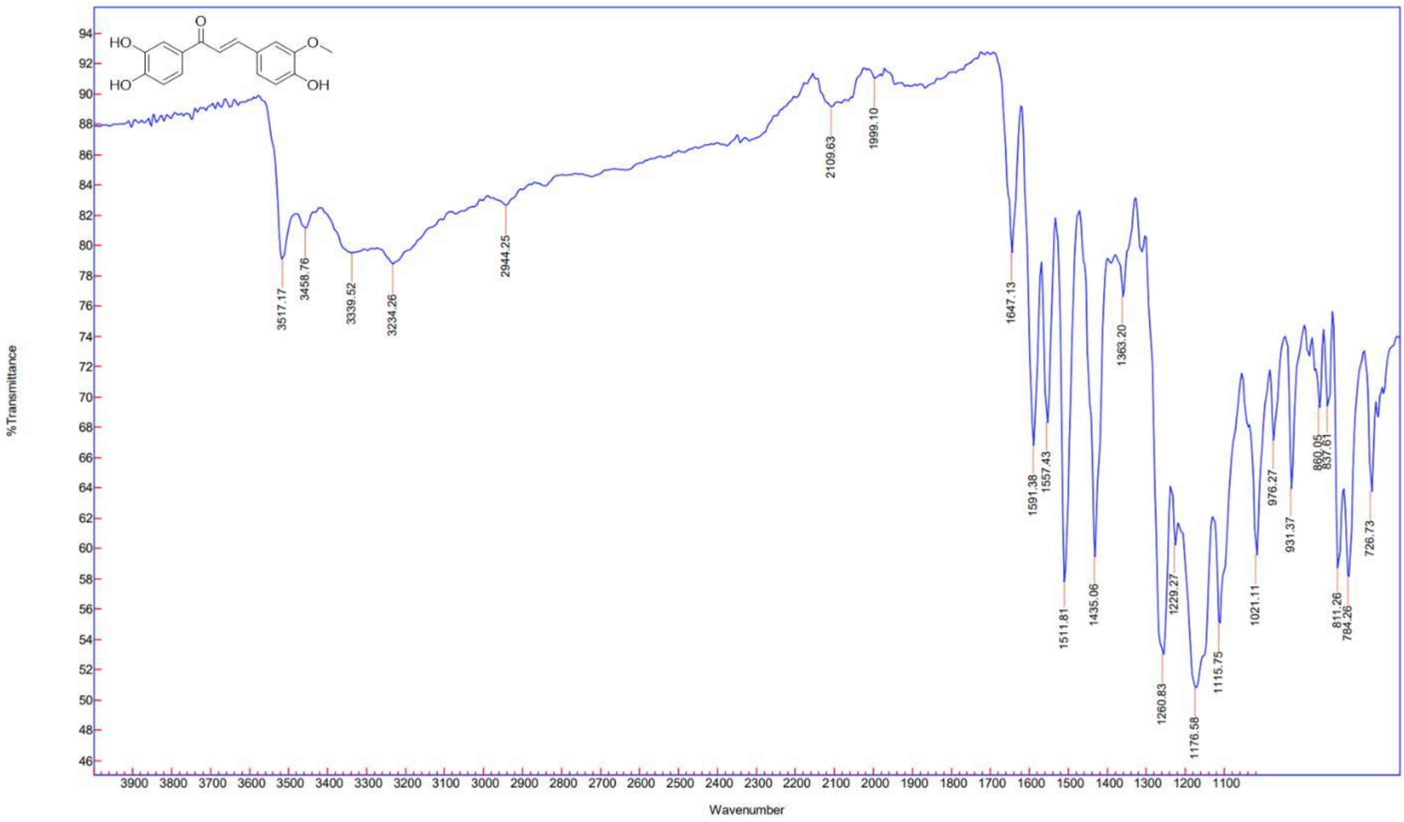
%Transmittance
Wavenumber
3517.17
3458.76
3339.52
3234.26
2944.25
2109.63
1999.10
1647.13
1591.38
1557.43
1511.81
1435.06
1363.20
1260.83
1229.27
1176.58
1115.75
1021.11
976.27
931.37
860.05
837.61
811.26
784.26
726.73

a9

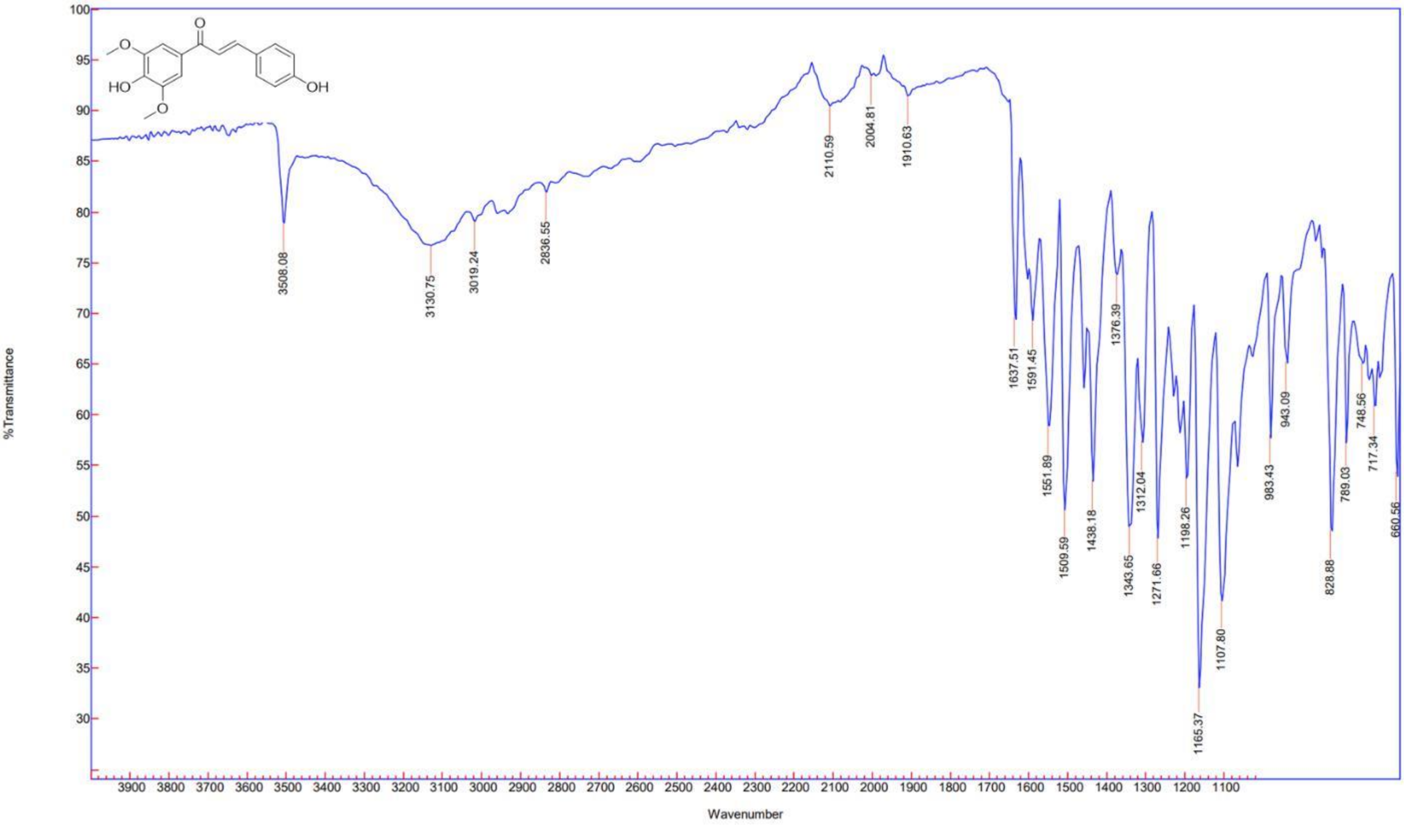
%Transmittance
Wavenumber
3508.08
3130.75
3019.24
2836.55
2110.59
2004.81
1910.63
1637.51
1591.45
1551.89
1509.59
1438.18
1376.39
1343.65
1312.04
1271.66
1198.26
1165.37
1107.80
983.43
943.09
828.88
789.03
748.56
717.34
660.56

a10

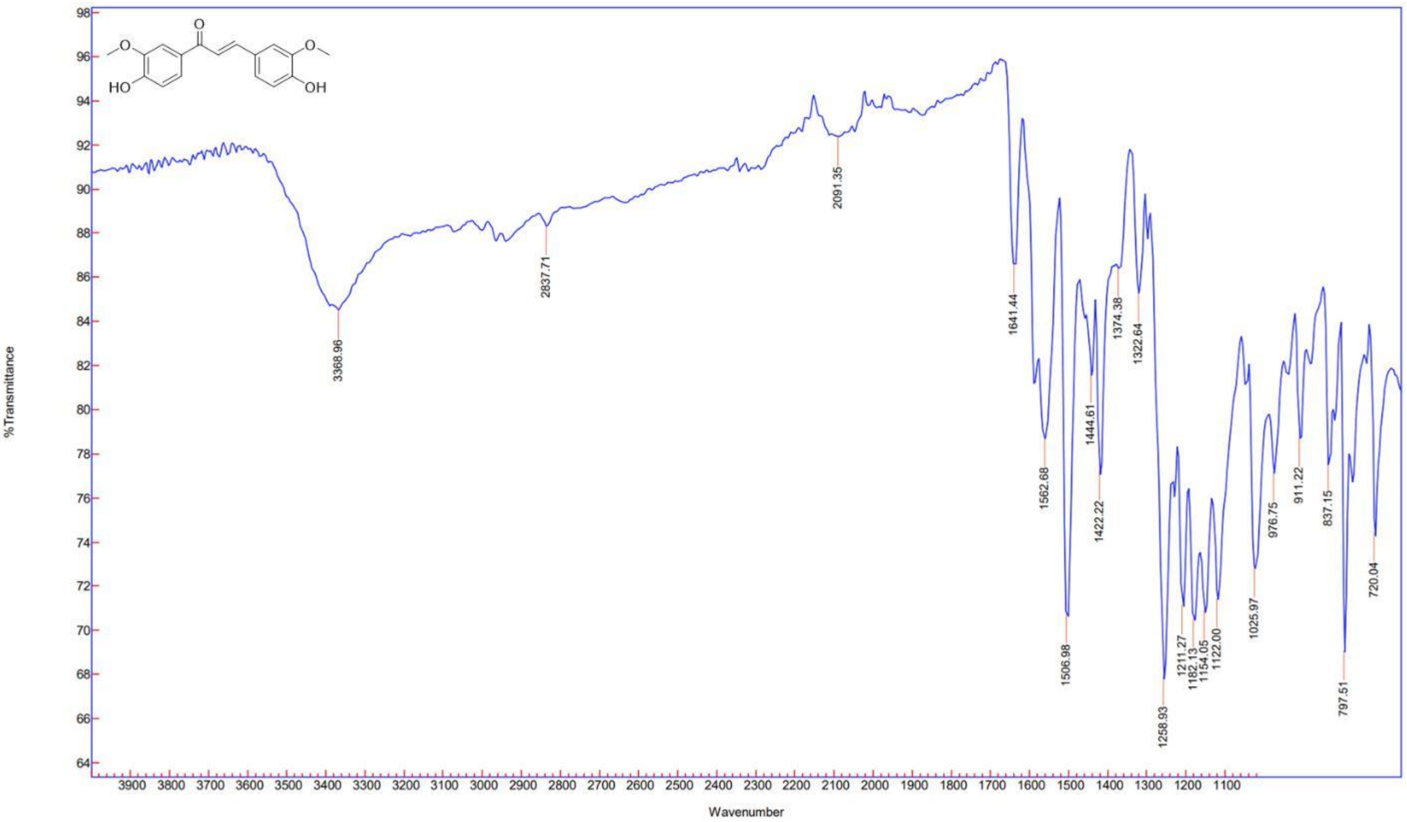
%Transmittance
Wavenumber
3368.96
2837.71
2091.35
1641.44
1562.68
1506.98
1444.61
1422.22
1374.38
1322.64
1258.93
1211.27
1182.13
1154.05
1122.00
1025.97
976.75
911.22
837.15
797.51
720.04
HO
OH

a11

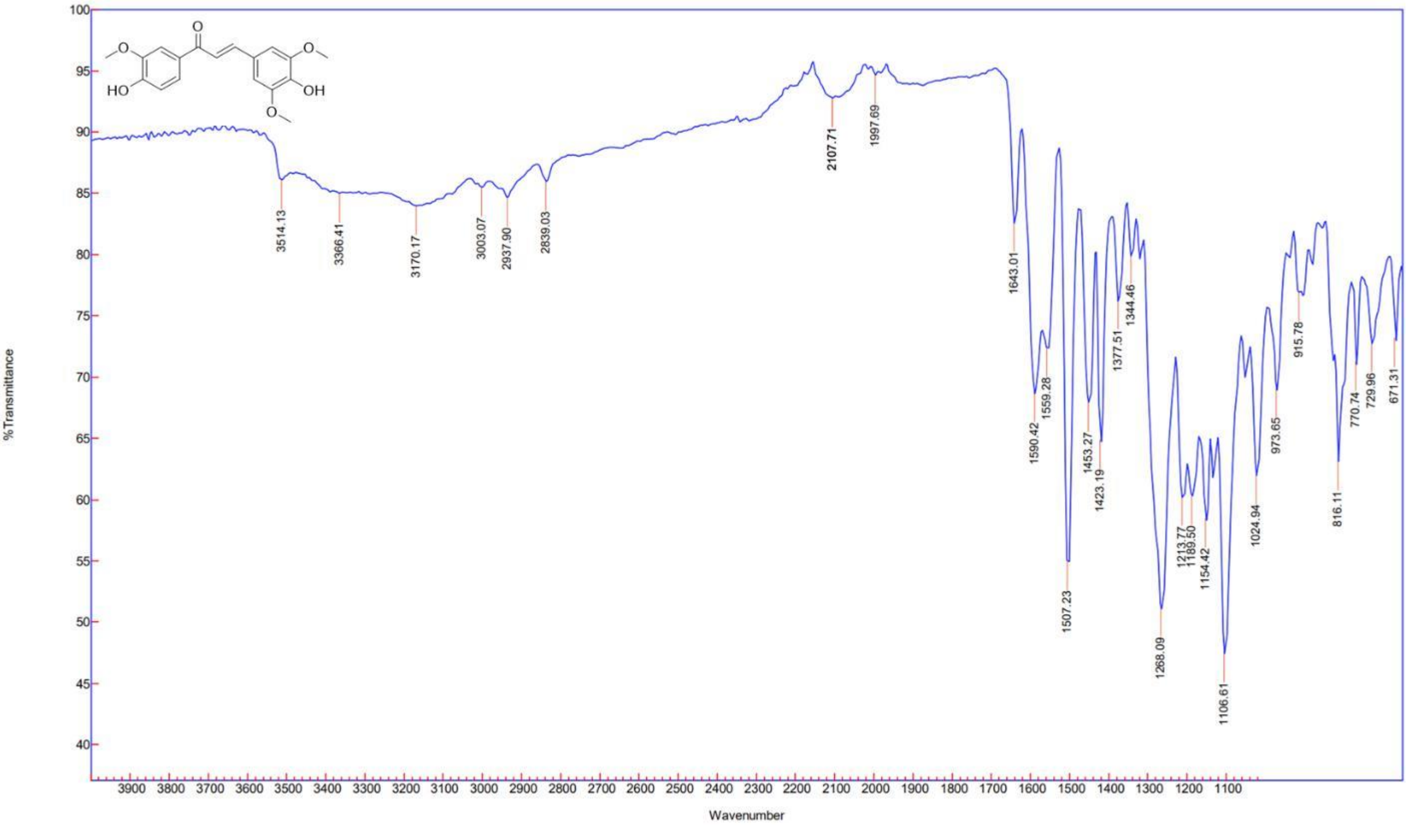
%Transmittance
Wavenumber
3514.13
3366.41
3170.17
3003.07
2937.90
2839.03
2107.71
1997.69
1643.01
1590.42
1559.28
1507.23
1453.27
1423.19
1377.51
1344.46
1268.09
1213.77
1189.50
1154.42
1106.61
1024.94
973.65
915.78
816.11
770.74
729.96
671.31
HO
OH

a12

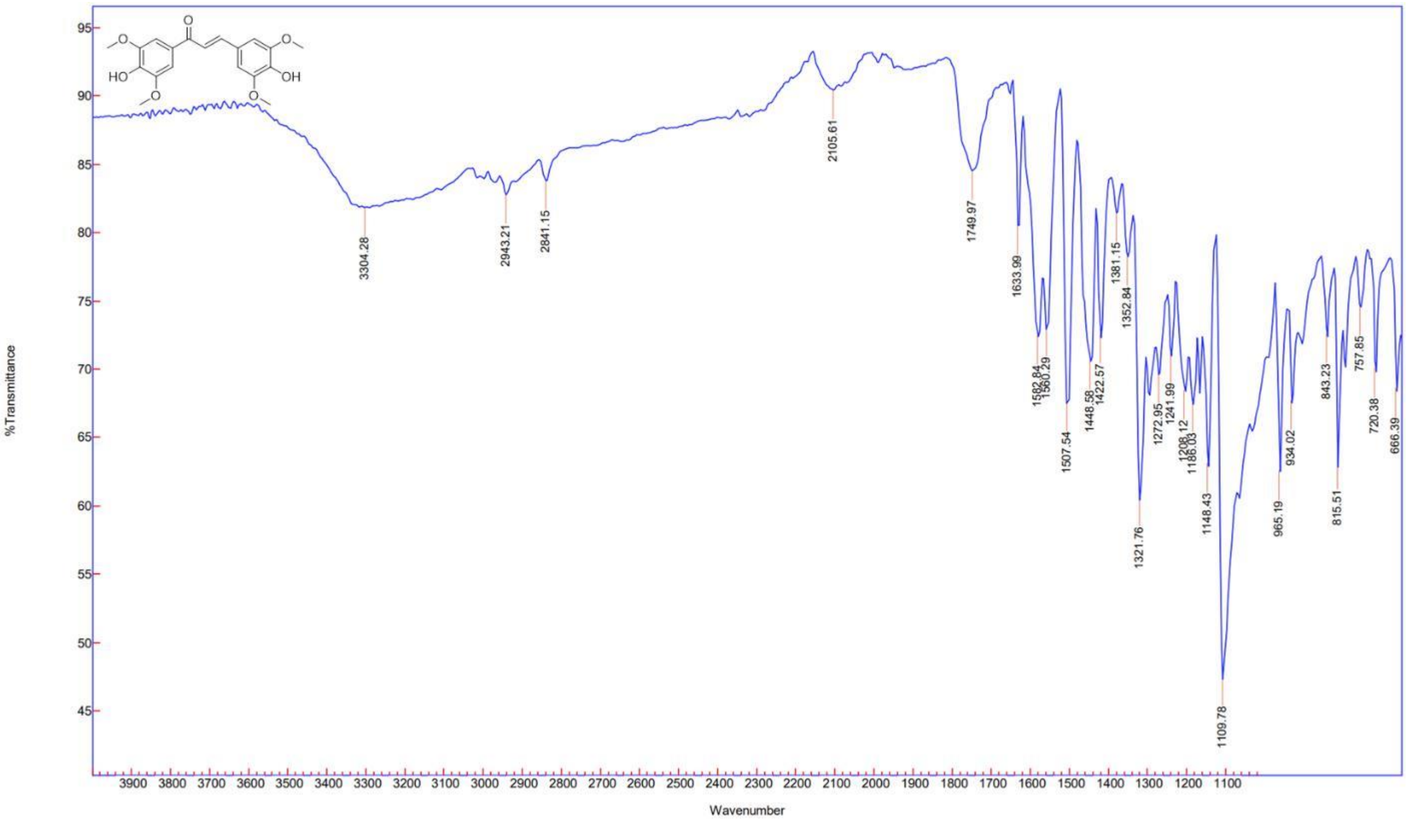
HO
OH
%Transmittance
Wavenumber
3304.28
2943.21
2841.15
2105.61
1749.97
1633.99
1582.84
1560.29
1507.54
1448.58
1422.57
1381.15
1352.84
1321.76
1272.95
1241.99
1208.12
1186.03
1148.43
1109.78
965.19
934.02
843.23
815.51
757.85
720.38
666.39

b1

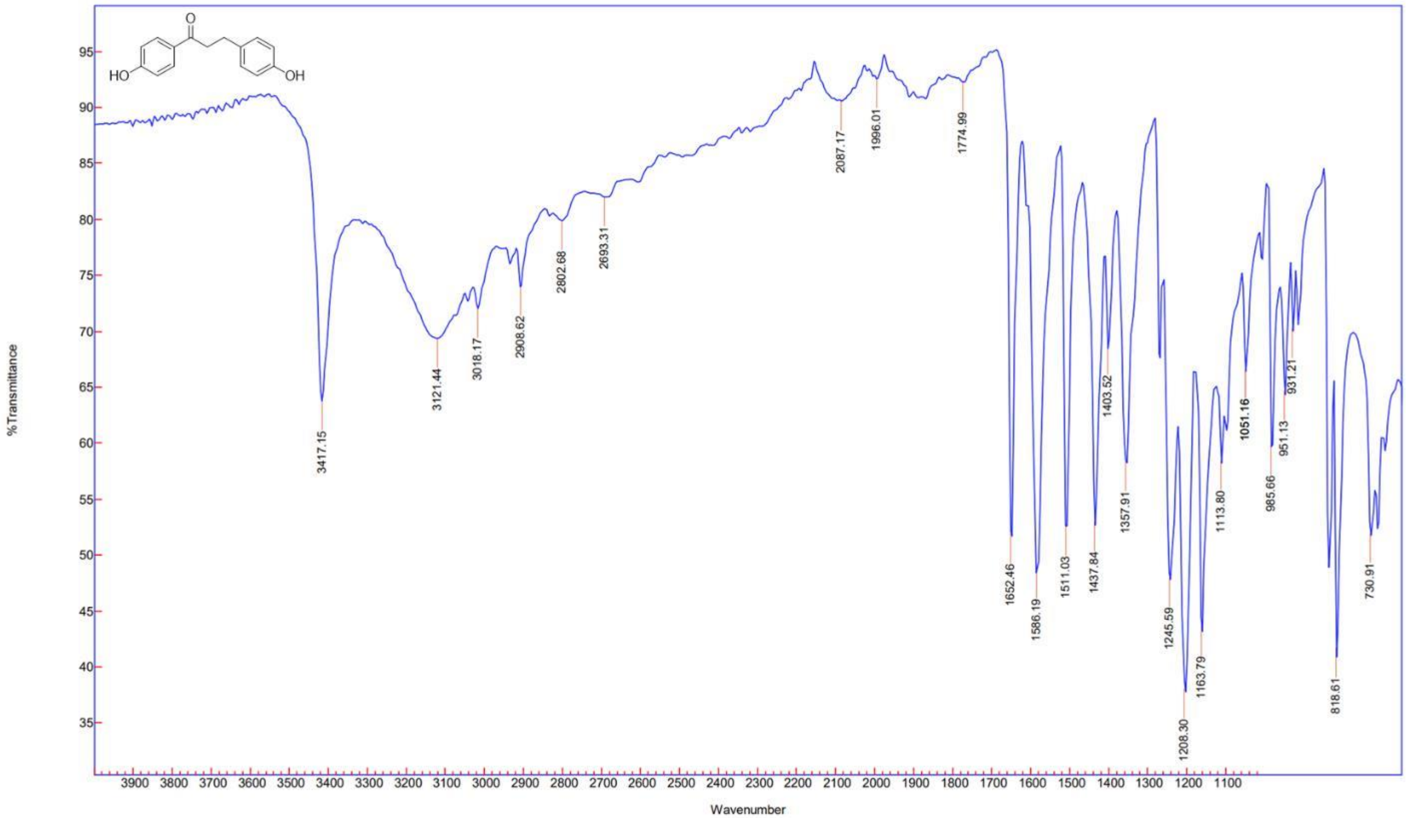
HO
OH
%Transmittance
Wavenumber
3417.15
3121.44
3018.17
2908.62
2802.68
2693.31
2087.17
1996.01
1774.99
1652.46
1586.19
1511.03
1437.84
1403.52
1357.91
1245.59
1208.30
1163.79
1113.80
1051.16
985.66
951.13
931.21
818.61
730.91

b2

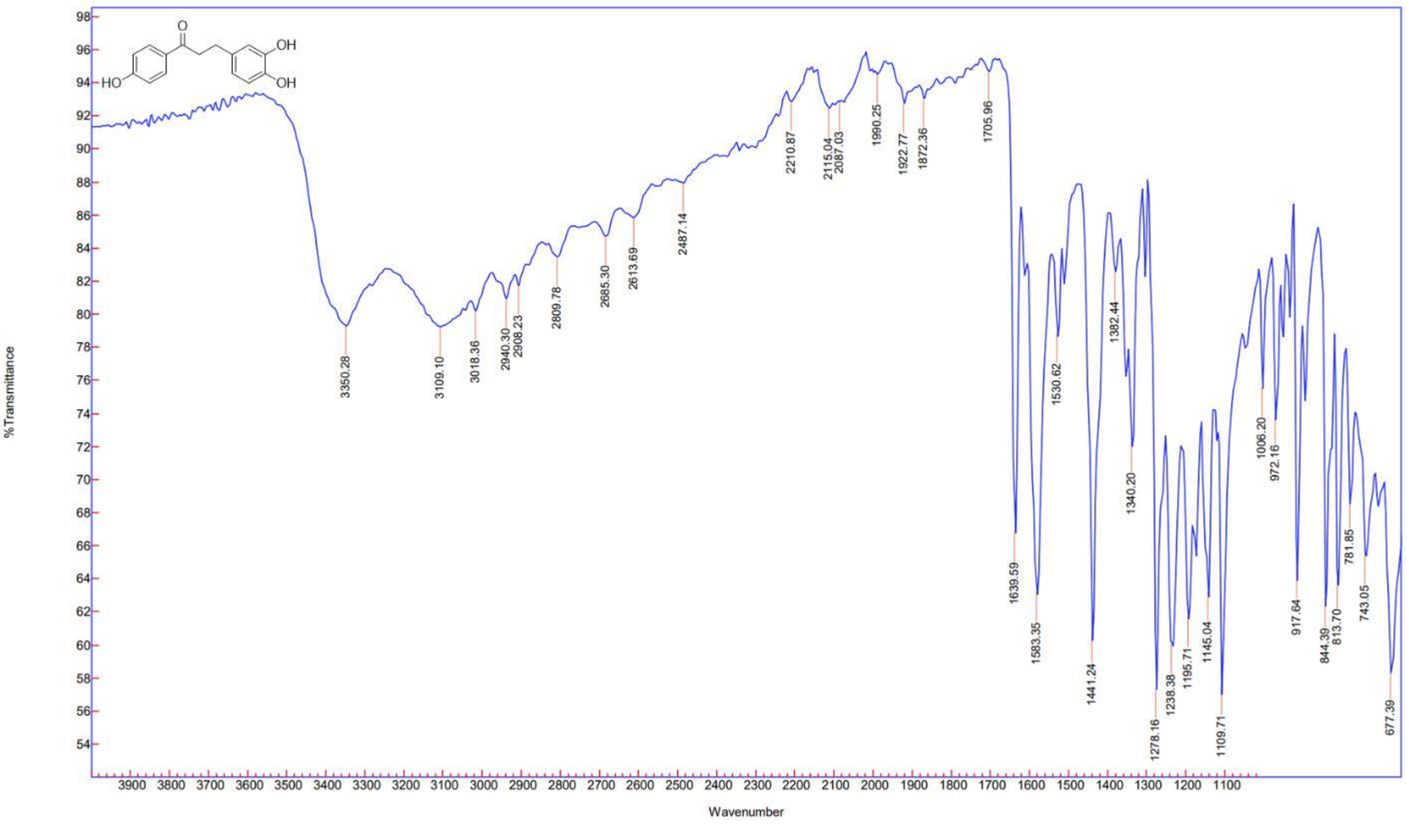

HO
OH
OH
% Transmittance
Wavenumber
3350.28
3109.10
3018.36
2940.30
2908.23
2809.78
2685.30
2613.69
2487.14
2210.87
2115.04
2087.03
1990.25
1922.77
1872.36
1705.96
1639.59
1583.35
1530.62
1441.24
1382.44
1340.20
1278.16
1238.38
1195.71
1145.04
1109.71
1006.20
972.16
917.64
844.39
813.70
781.85
743.05
677.39

b3

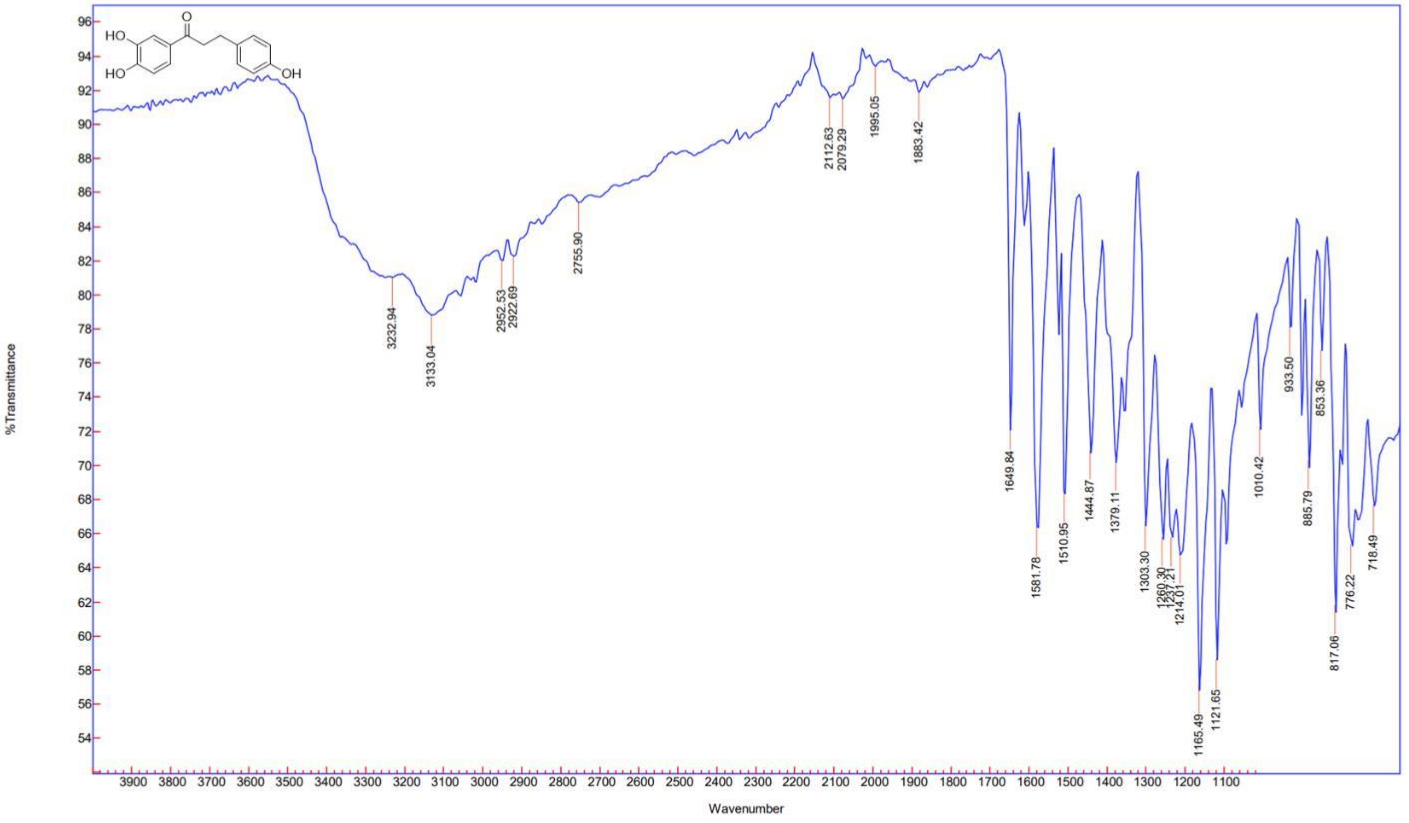

HO
HO
OH
% Transmittance
Wavenumber
3232.94
3133.04
2952.53
2922.69
2755.90
2112.63
2079.29
1995.05
1883.42
1649.84
1581.78
1510.95
1444.87
1379.11
1303.30
1260.30
1214.01
1165.49
1121.65
1010.42
933.50
885.79
853.36
817.06
776.22
718.49

b4

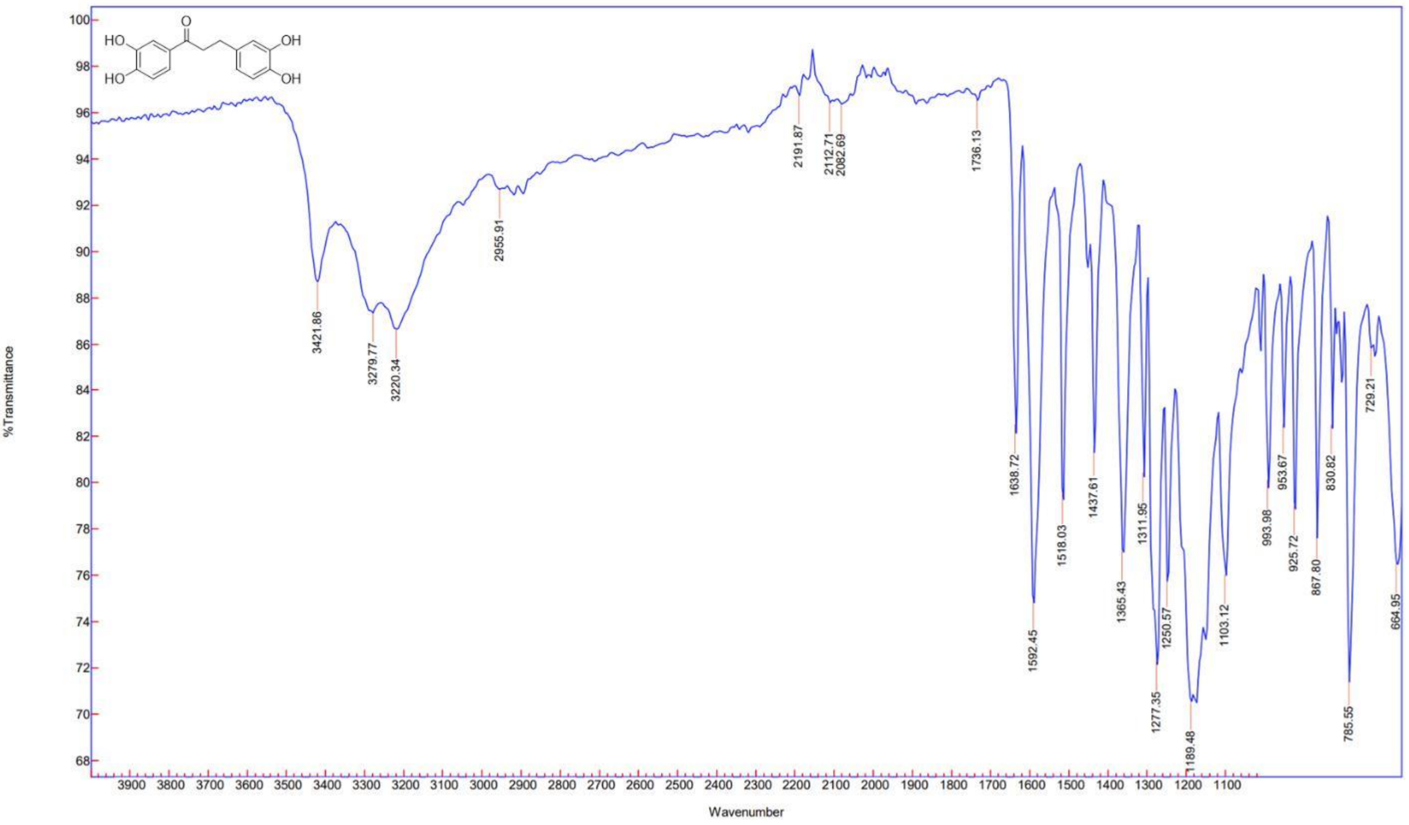
HO
HO
O
OH
OH
%Transmittance
Wavenumber
3421.86
3279.77
3220.34
2955.91
2191.87
2112.71
2082.69
1736.13
1638.72
1592.45
1518.03
1437.61
1365.43
1311.95
1277.35
1250.57
1189.48
1103.12
993.98
953.67
925.72
867.80
830.82
785.55
729.21
664.95

b5

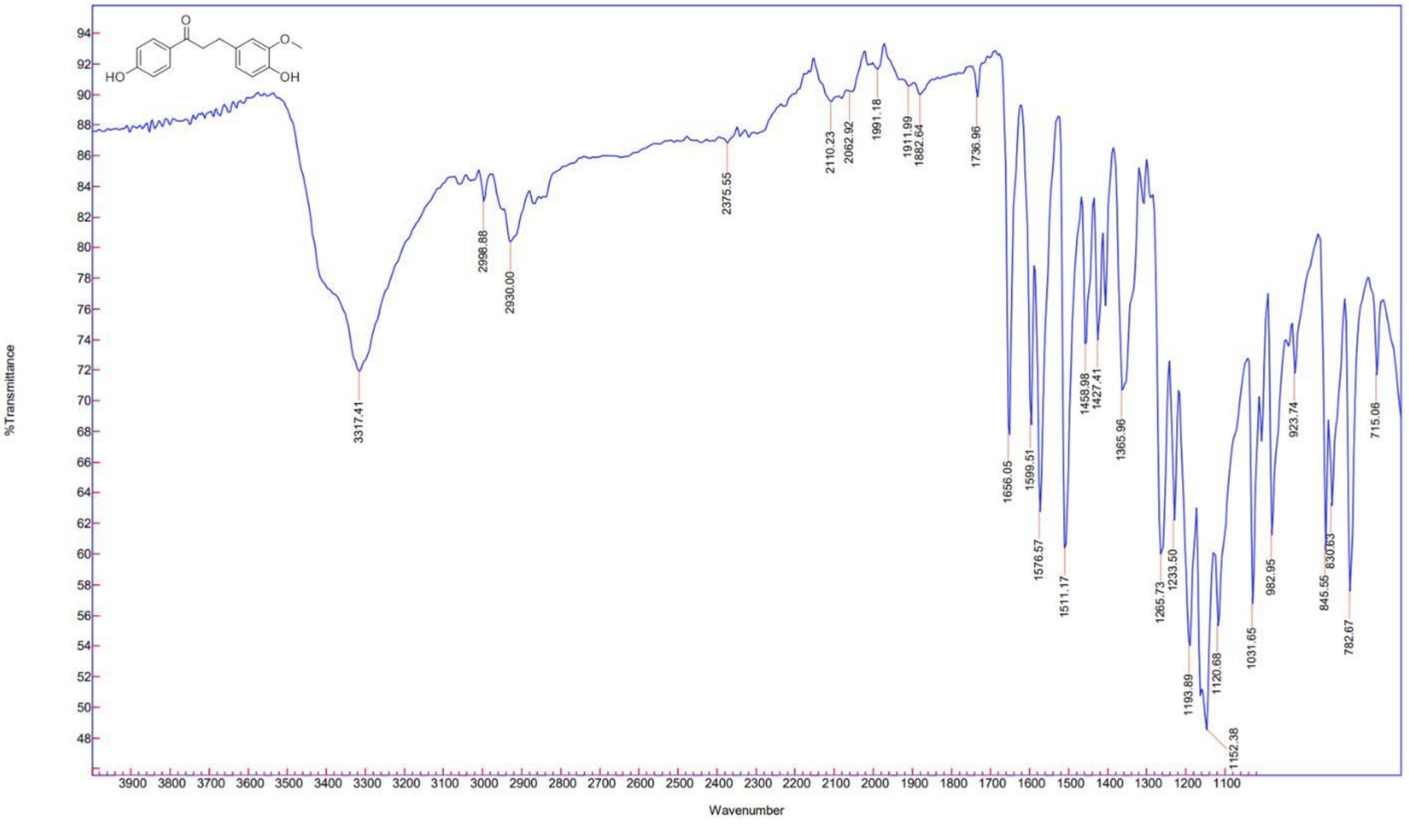
HO
O
O
OH
%Transmittance
Wavenumber
3317.41
2998.88
2930.00
2375.55
2110.23
2062.92
1991.18
1911.99
1882.64
1736.96
1656.05
1599.51
1576.57
1511.17
1458.98
1427.41
1365.96
1265.73
1233.50
1193.89
1152.38
1120.68
1031.65
982.95
923.74
845.55
830.63
782.67
715.06

b6

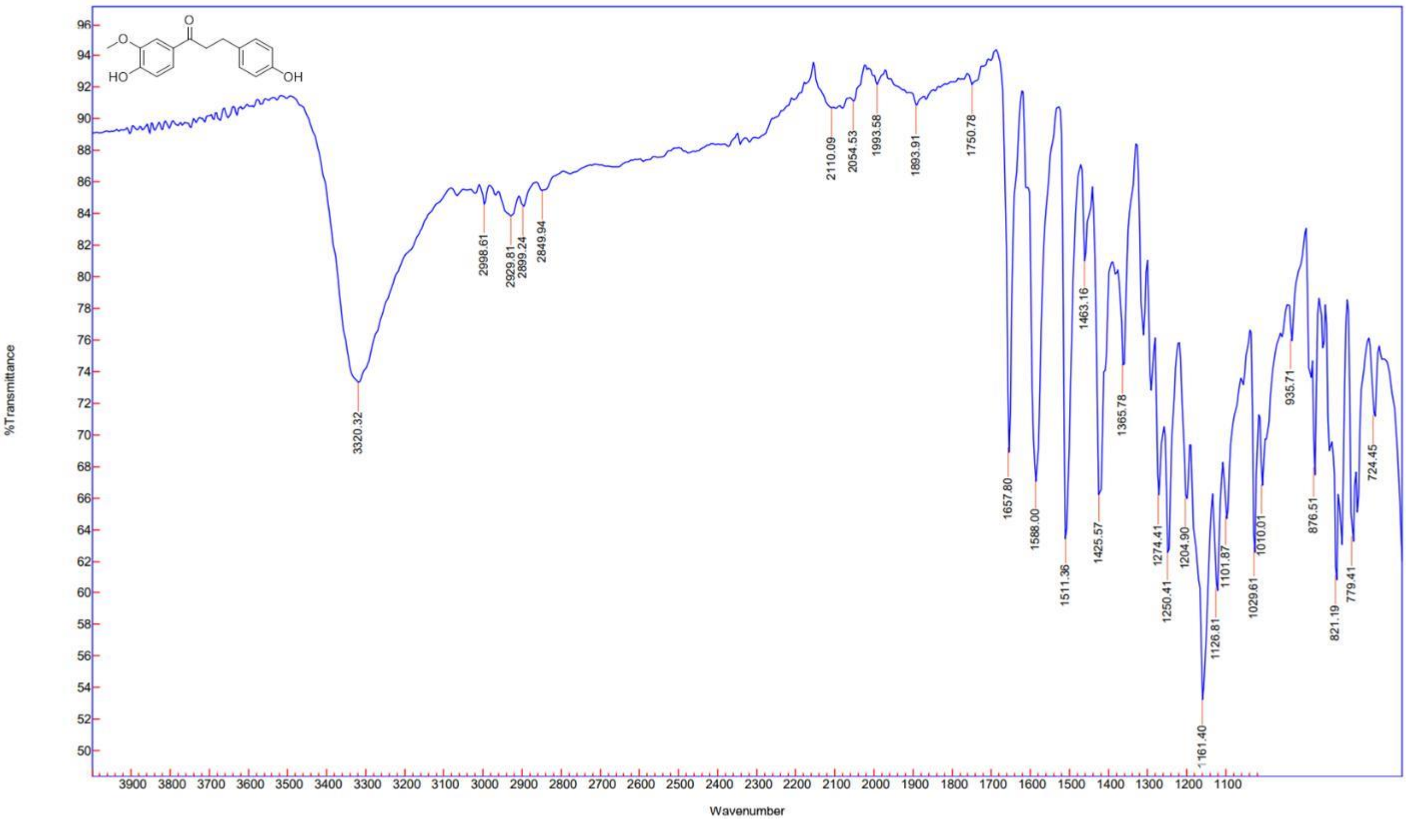
HO
OH
%Transmittance
Wavenumber
3320.32
2998.61
2929.81
2899.24
2849.94
2110.09
2054.53
1993.58
1893.91
1750.78
1657.80
1588.00
1511.36
1463.16
1425.57
1365.78
1274.41
1250.41
1204.90
1161.40
1126.81
1101.87
1029.61
1010.01
935.71
876.51
821.19
779.41
724.45

b7

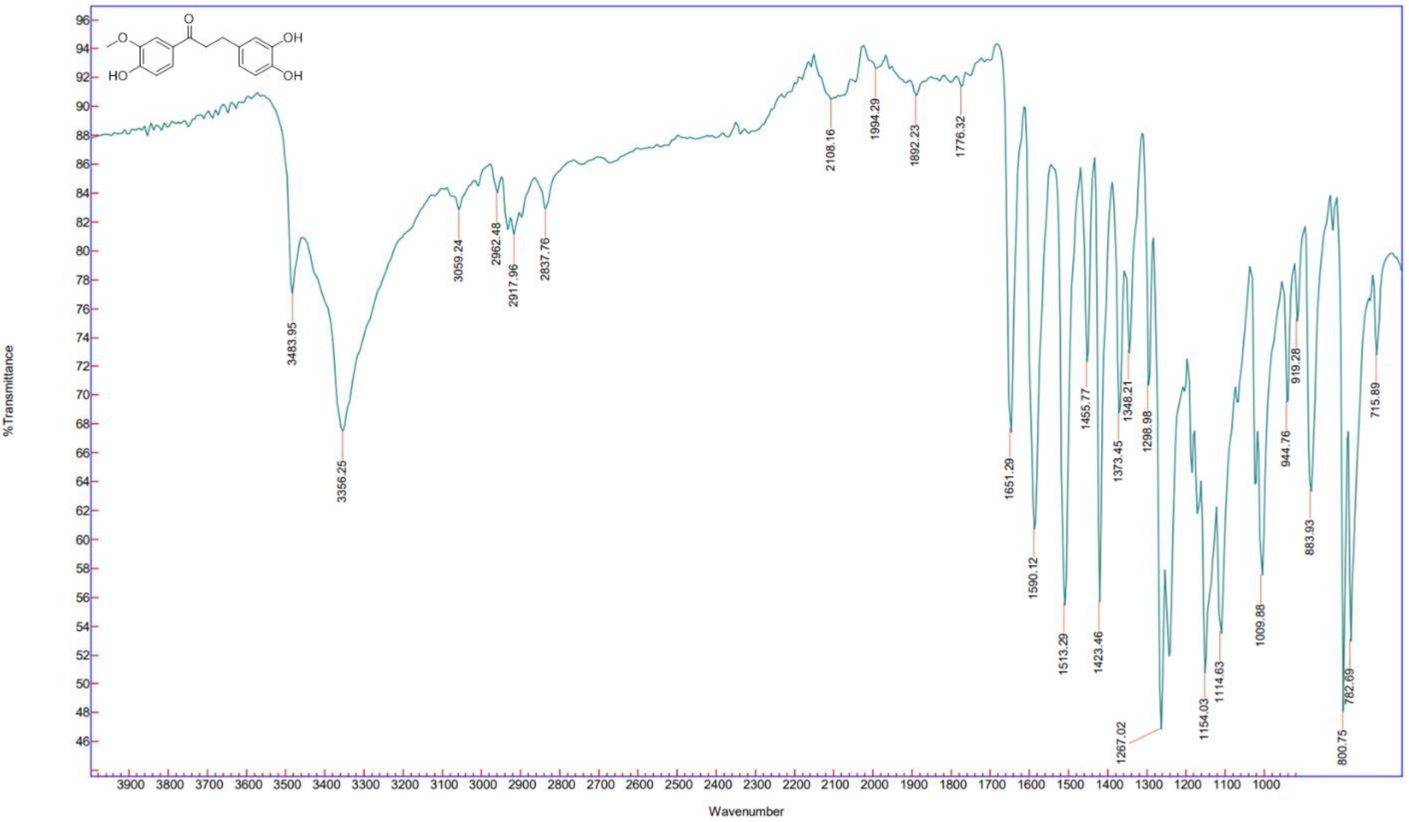
HO
OH
OH
%Transmittance
Wavenumber
3483.95
3356.25
3059.24
2962.48
2917.96
2837.76
2108.16
1994.29
1892.23
1776.32
1651.29
1590.12
1513.29
1455.77
1423.46
1373.45
1348.21
1298.98
1267.02
1154.03
1114.63
1009.88
944.76
919.28
883.93
800.75
782.69
715.89

b8

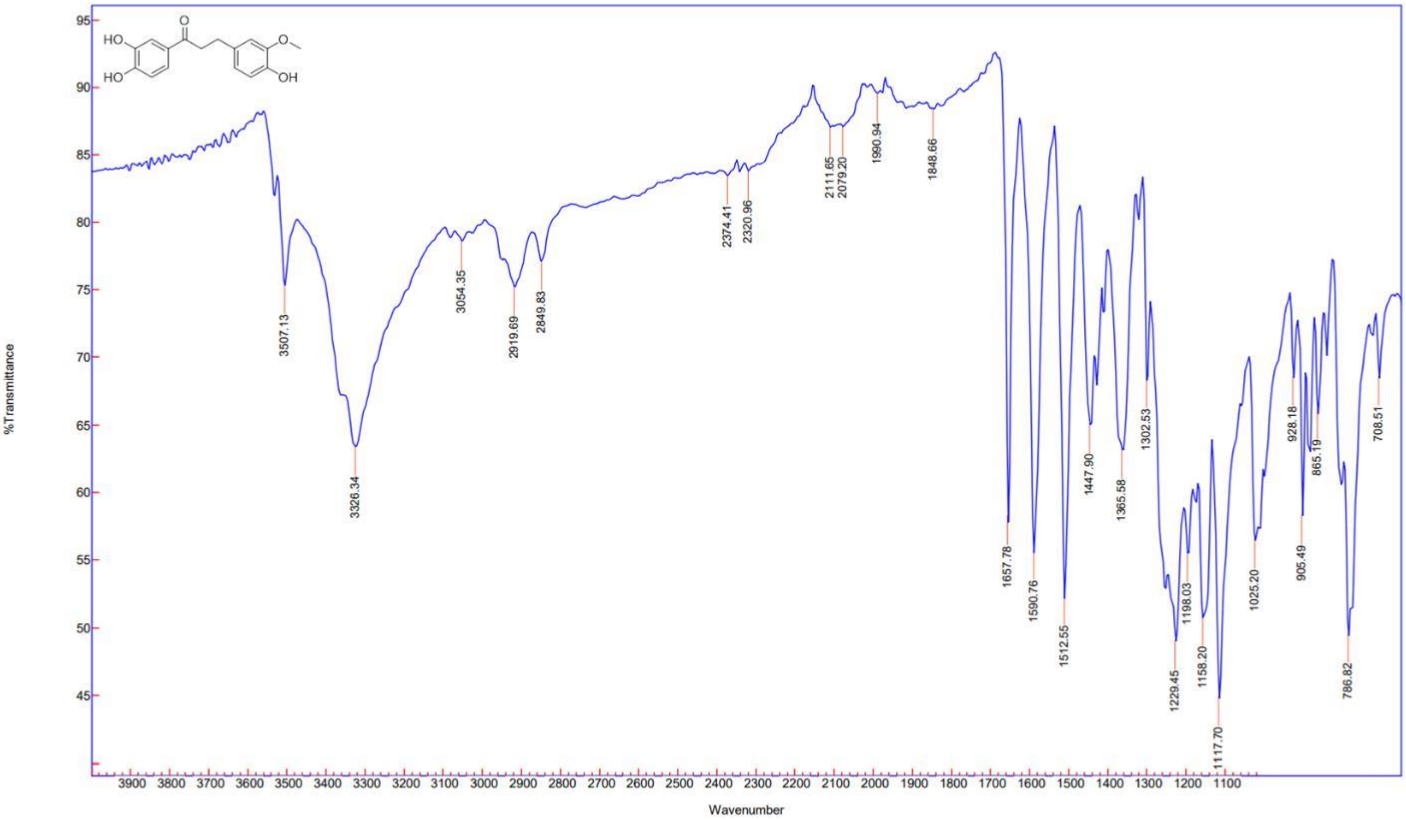
HO
HO
O
OH
%Transmittance
Wavenumber
3507.13
3326.34
3054.35
2919.69
2849.83
2374.41
2320.96
2111.65
2079.20
1990.94
1848.66
1657.78
1590.76
1512.55
1447.90
1365.58
1302.53
1229.45
1198.03
1158.20
1117.70
1025.20
928.18
905.49
865.19
786.82
708.51

b9

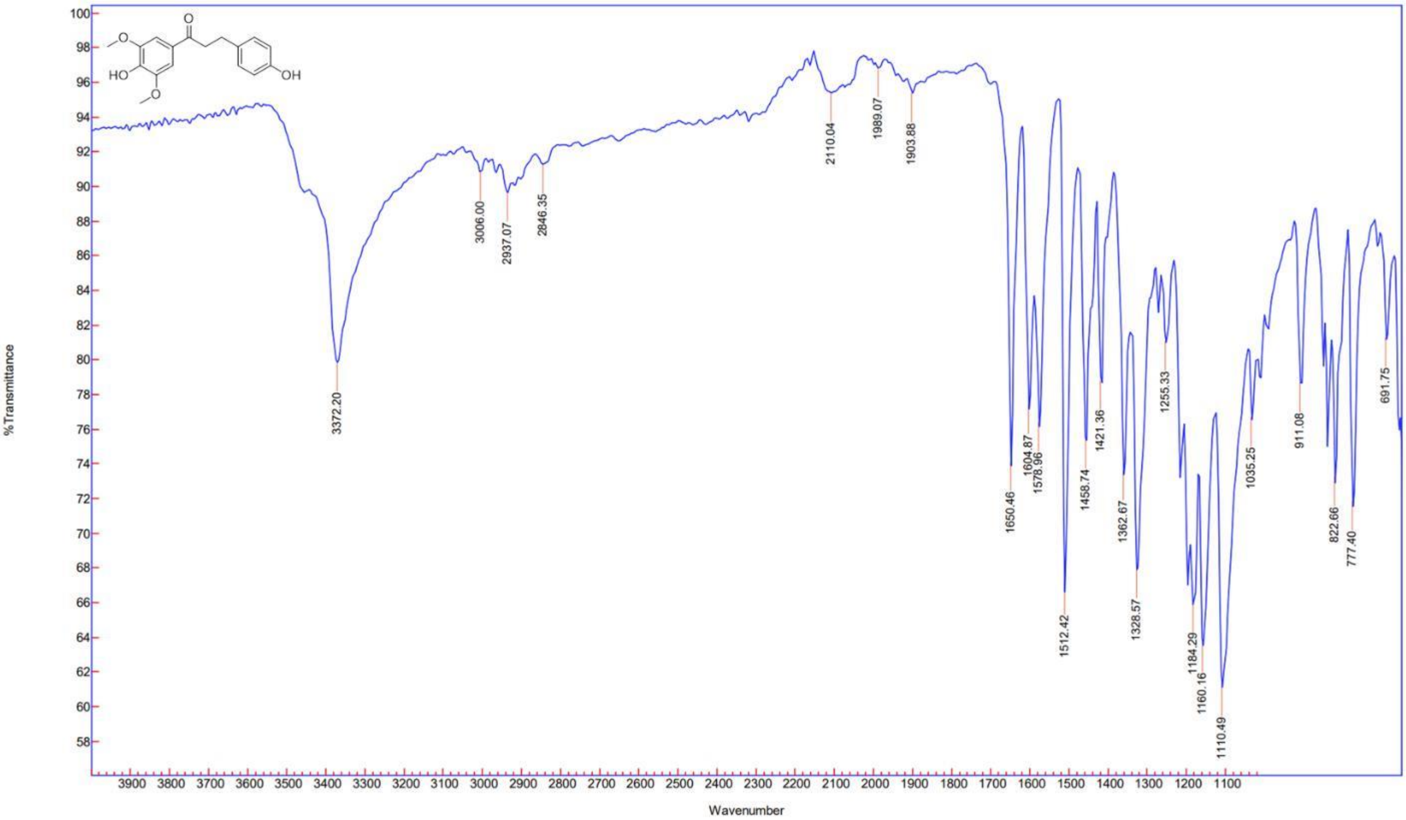
HO
O
OH
%Transmittance
Wavenumber
3372.20
3006.00
2937.07
2846.35
2110.04
1989.07
1903.88
1650.46
1604.87
1578.96
1512.42
1458.74
1421.36
1362.67
1328.57
1255.33
1184.29
1160.16
1110.49
1035.25
911.08
822.66
777.40
691.75

b10

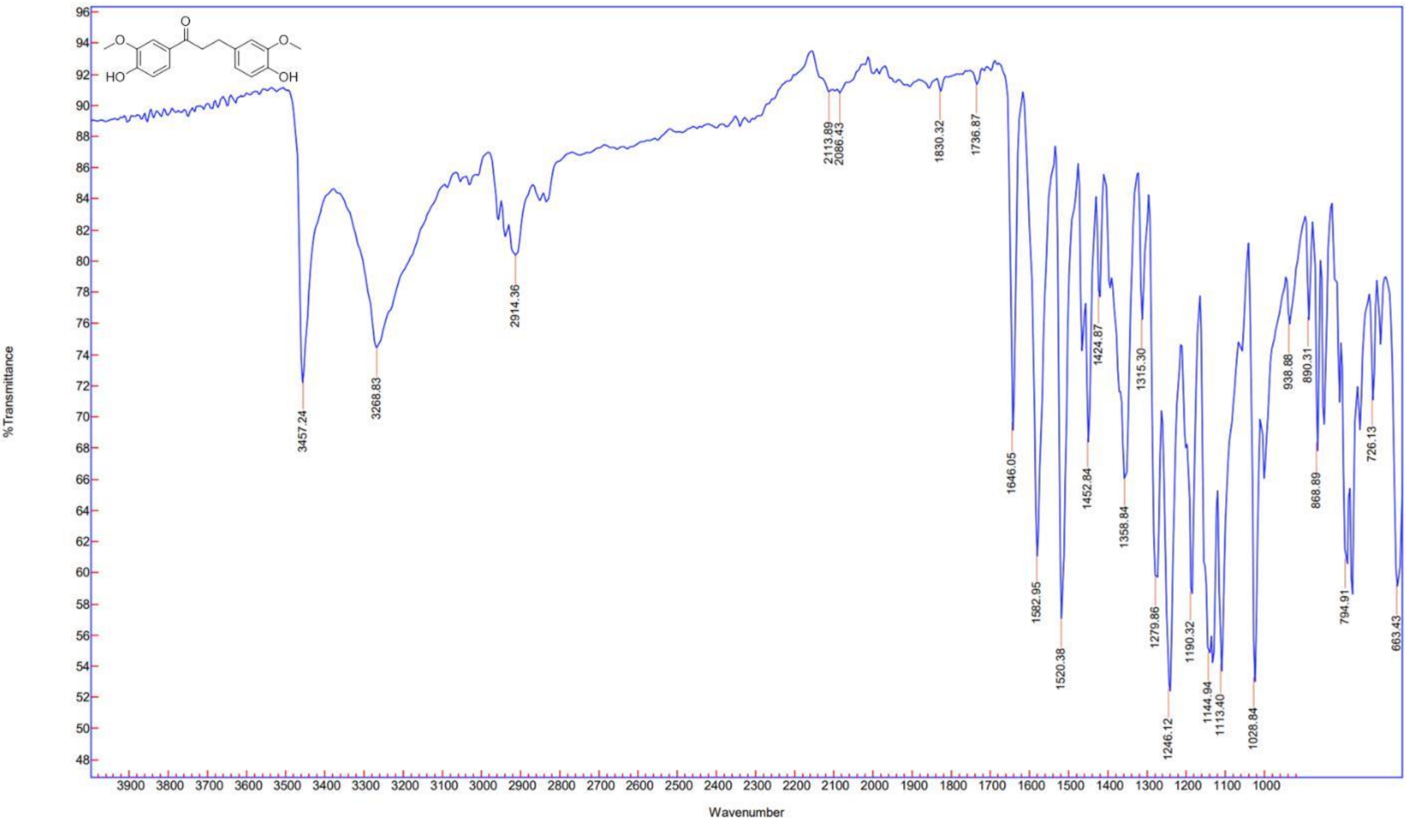
HO
OH
%Transmittance
Wavenumber
3457.24
3268.83
2914.36
2113.89
2086.43
1830.32
1736.87
1646.05
1582.95
1520.38
1452.84
1424.87
1358.84
1315.30
1279.86
1246.12
1190.32
1144.94
1113.40
1028.84
938.88
890.31
868.89
794.91
726.13
663.43

b11

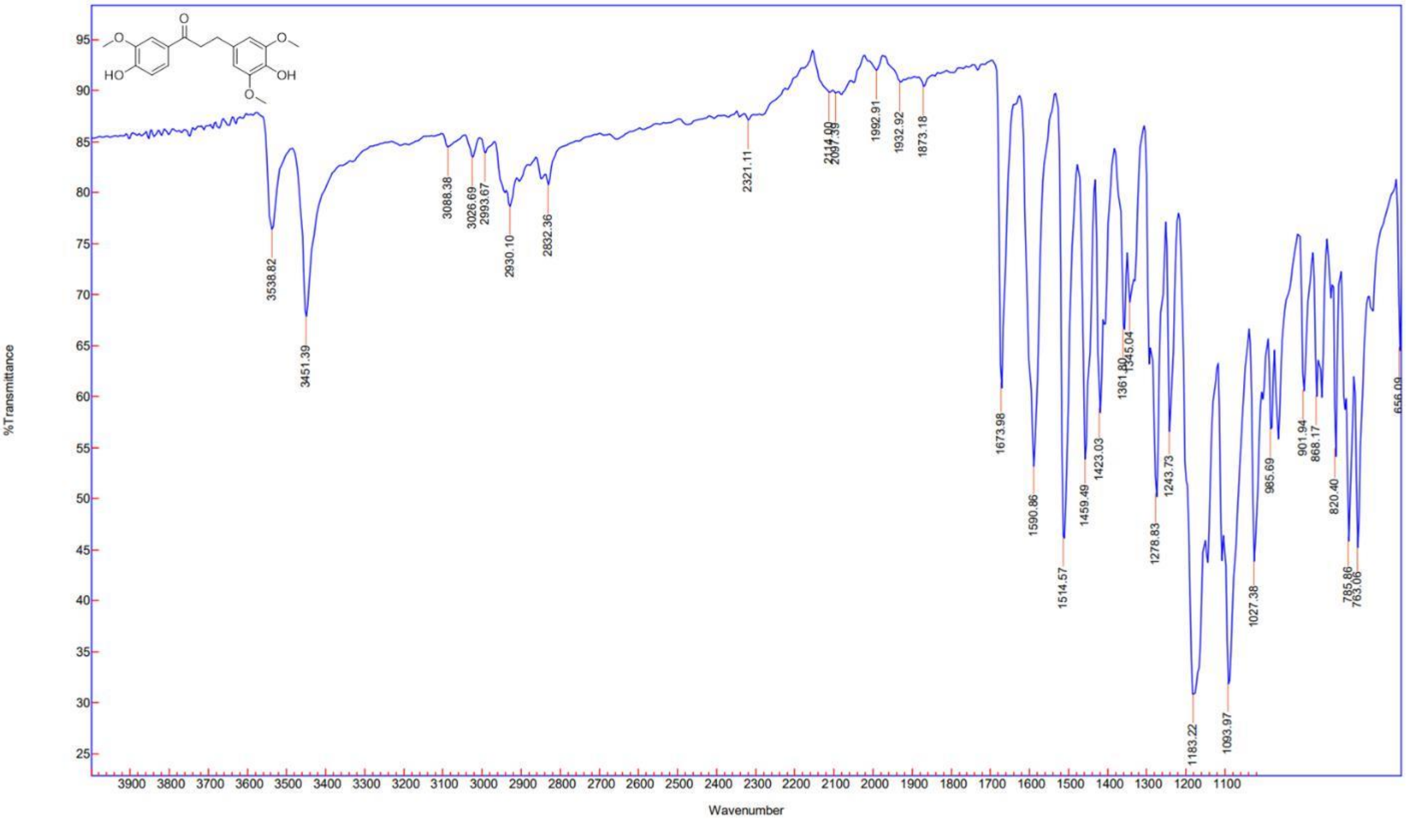
HO
OH
%Transmittance
Wavenumber
3538.82
3451.39
3088.38
3026.69
2993.67
2930.10
2832.36
2321.11
1992.91
1932.92
1873.18
1673.98
1590.86
1514.57
1459.49
1423.03
1278.83
1243.73
1183.22
1093.97
1027.38
985.69
901.94
868.17
820.40
785.86
763.06

b12

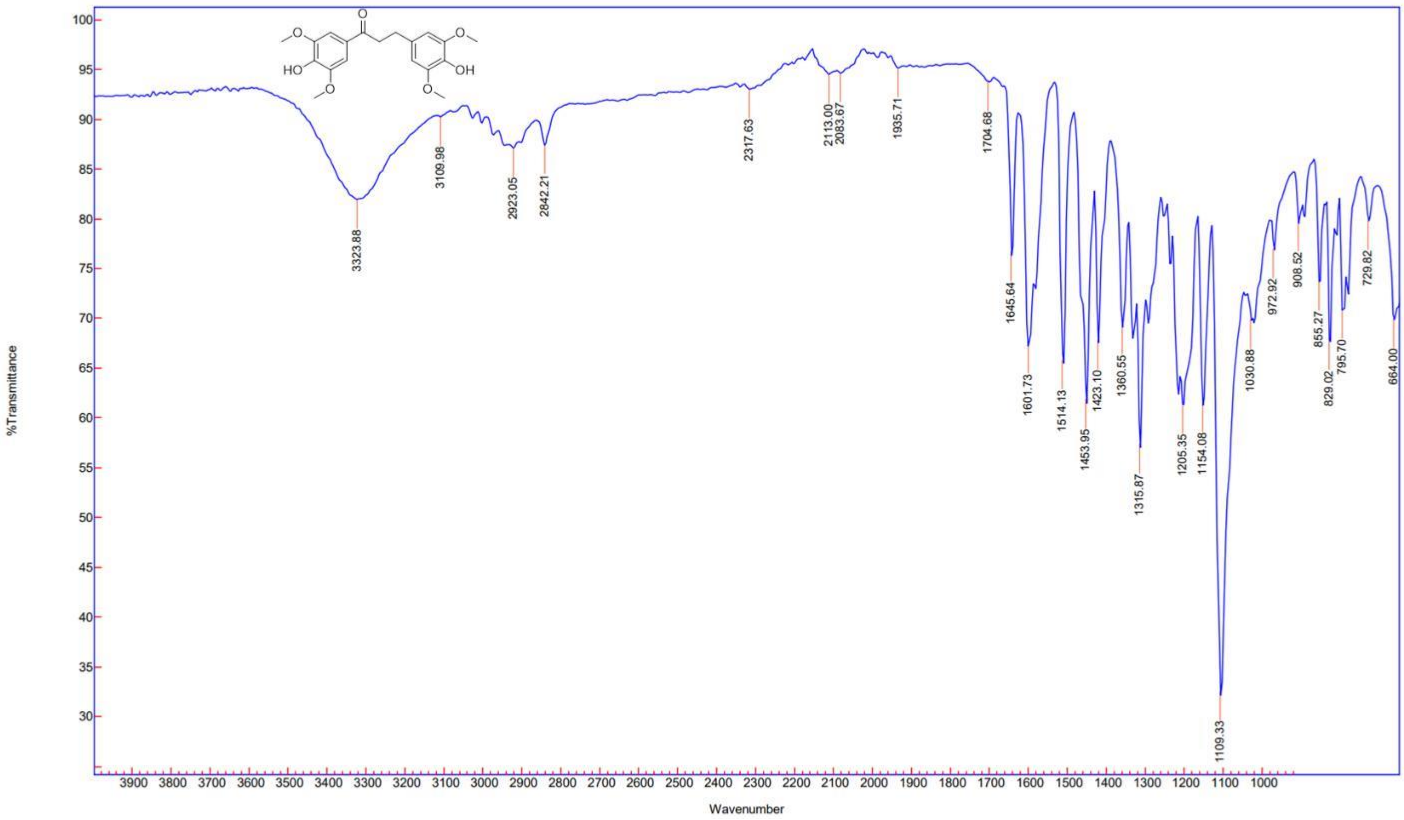
HO
OH
%Transmittance
Wavenumber
3323.88
3109.98
2923.05
2842.21
2317.63
2113.00
2083.67
1935.71
1704.68
1645.64
1601.73
1514.13
1453.95
1423.10
1360.55
1315.87
1205.35
1154.08
1109.33
1030.88
972.92
908.52
855.27
829.02
795.70
729.82
664.00
